# Twin mitochondrial sequence analysis

**DOI:** 10.1002/mgg3.20

**Published:** 2013-06-26

**Authors:** Yosr Bouhlal, Selena Martinez, Henry Gong, Kevin Dumas, Joseph T C Shieh

**Affiliations:** 1 Division of Medical Genetics, Department of Pediatrics, University of California San FranciscoSan Francisco, California; 2 Institute for Human Genetics, University of California San FranciscoSan Francisco, California

**Keywords:** Genome, heteroplasmy, mitochondrial, primer extension, sequencing, twins

## Abstract

When applying genome-wide sequencing technologies to disease investigation, it is increasingly important to resolve sequence variation in regions of the genome that may have homologous sequences. The human mitochondrial genome challenges interpretation given the potential for heteroplasmy, somatic variation, and homologous nuclear mitochondrial sequences (numts). Identical twins share the same mitochondrial DNA (mtDNA) from early life, but whether the mitochondrial sequence remains similar is unclear. We compared an adult monozygotic twin pair using high-throughput sequencing and evaluated variants with primer extension and mitochondrial preenrichment. Thirty-seven variants were shared between the twin individuals, and the variants were verified on the original genomic DNA. These studies support highly identical genetic sequence in this case. Certain low-level variant calls were of high quality and homology to the mtDNA, and they were further evaluated. When we assessed calls in preenriched mtDNA templates, we found that these may represent numts, which can be differentiated from mtDNA variation. We conclude that twin identity extends to mtDNA, and it is critical to differentiate between numts and mtDNA in genome sequencing, particularly as significant heteroplasmy could influence genome interpretation. Further studies on mtDNA and numts will aid in understanding how variation occurs and persists.

## Introduction

Mitochondria play important roles in cellular function and human disease (Poulton et al. 2010; Koopman et al. 2012) and harbor DNA that encodes for tRNAs, rRNAs, and for proteins that function in energy production. Unlike the nuclear genome, the mitochondrial DNA (mtDNA) is present in many copies within the same cell. The mtDNA sequence reflects the maternally inherited mitochondrial genome and could harbor variation that arises somatically (Park and Larsson 2011). mtDNA may be subject to a higher mutation rate due to apparent decreased replication fidelity (Song et al. 2003; Lee and Johnson 2006); however, it is unclear whether mutations accumulate (Kujoth et al. 2007; Ameur et al. 2011) particularly in humans or whether variation is preexisting. Variation within the mtDNA sequence in an individual's cells can be homoplasmic (the same sequence) or heteroplasmic (coexisting different mtDNA sequences), and heteroplasmy levels can be related to disease (Lightowlers et al. 1997). Due to the segregation of the mtDNA during cell division, mtDNA variation could differ between cells as they divide, potentially due to selection. Such variation has been assessed in maternal transmission but the variation has not been examined in depth in adults, where multiple influences over time could affect sequence.

Deep sequencing is increasingly being utilized to detect genomic variation, and as more human genome sequencing is performed, it is clearly important to accurately detect mitochondrial variation and annotate variant function (Ruiz-Pesini et al. 2007; Calvo et al. 2012). However, mitochondrial variation can be misinterpreted given the presence of nuclear mitochondrial sequences (numts), which are highly similar nuclear fragments of the mitochondrial genome located on different chromosomes (Hazkani-Covo et al. 2010). Indeed, some studies report the difficulties in estimation of variation due to coamplification of numts with the mtDNA (Hirano et al. 1997; Parfait et al. 1998; Parr et al. 2006); however, a combination of high-throughput sequencing and validation could aid in thoroughly characterizing mitochondrial variation.

In our study, we evaluate and compare the presence of variants in mtDNA of an identical twin pair. Resulting from an early split of the developing embryo, twins harbor genomes that would be identical initially. Such twins represent a model for the analysis of mtDNA variation using a variety of methods, as any genetic difference observed between twins derived from the same zygote could represent somatic variation (Dumanski and Piotrowski 2012), whereas concordant variations found in twins would support genetic similarity (Bruder et al. 2008; Baranzini et al. 2010; Hallmayer et al. 2011; Jakobsen et al. 2011). Low levels of variation between twins could be detected in mitochondrial sequence. Here, we analyzed an adult twin pair with complementary methods to investigate variation in the mitochondrial genome sequence, and we determine the potential origin of ambiguous sequence variants from deep sequencing.

## Materials and Methods

### DNA isolation and molecular analysis

Informed consent was obtained under the guidelines of the institutional review board. Genomic DNA was isolated from the peripheral blood of a self-reported 21-year-old female adult identical twin pair, using standard methods (Qiagen, Valencia, CA). The twins grew up together and lived in a similar environment.

Zygosity of the twins was determined by genotyping highly polymorphic DNA loci using the PowerPlex® short tandem repeat kit (Promega, Sunnyvale, CA). Amplified fragments were detected using the ABI (Life Technologies, Grand Island, NY) 3730xl DNA Analyzer. Data were analyzed with GeneMapper software (Life Technologies).

### Paired-end Illumina sequencing

DNA libraries were generated using Illumina Paired-End (Illumina Inc., San Diego, CA) library preparation kit according to the manufacturer's instructions. Libraries were quantified by qPCR using a KAPA (Kapa Biosystems Inc., Woburn, MA) library quantification kit and assessed on an Agilent Technologies (Santa Clara, CA) 2100 Bioanalyzer using a High Sensitivity DNA chip. Following an automatic cluster generation, paired-end sequencing was performed on Illumina HiSeq 2000 using the V1 flow cell Hiseq system version (Illumina Inc.). The libraries were subject to a second sequencing run using the V3 flow cell HiSeq system version.

### Analysis of high-throughput sequencing data

To check read quality, raw sequencing data were analyzed using the next generation tool Fastqc (http://www.bioinformatics.bbsrc.ac.uk/projects/fastqc/) executed in an internal Galaxy server (Giardine et al. 2005) allowing large data file upload and processing. Sequencing reads were mapped to the hg19 reference genome (NCBI GRCh37; AF347015.1 [16571 bp]/NC_012920.1 [16569 bp]) using the Bowtie alignment package (0.12.7). Alignment was performed using normal and high stringency parameters for each of the two runs. Mapped reads were made available in BAM file format using SAM tools. All BAM files were visualized and analyzed using the Integrative Genomic Viewer IGV 2.1 (Robinson et al. 2011). Mitochondrial sequence reads, produced from the second high stringency run, were filtered from the whole genome data and converted to small BAM files and analyzed with MITO-BAM annotator online tool (Zhidkov et al. 2011). Using “Generate pileup” from SAM tools, we created from the BAM files pileup files for the twins. The generated pileup files were used to call Single Nucleotide Polymorphisms (SNPs) via the “Filter pileup” tool. For each potential variant, we set a quality threshold *Q* ≥ 30 where *Q* is the Phred quality score, and asked the program to call any SNP present at least in one read. Using IGV tool, we manually verified the presence and the *Q* value of all the called SNPs. In addition, we used the MITOBAM annotator tool to detect and annotate any variant also present in at least one read and with a *Q* ≥ 30. We also verified all SNPs manually applying more stringent filters by not considering potential variants surrounded by more than 10 mismatches within the same read, and mismatches localized within the five first or last bases of the same read. Potential variants were identified as having RefSNP (rs) numbers, while some variants were only found in Mitomap mtDNA Sequence Polymorphism database (http://www.mitomap.org/MITOMAP), and others were previously not reported.

### Low-level variant evaluation

Variants detected at low frequency (<0.01%) were examined using the fluorescent primer extension assay SnapShot (Applied Biosystems, Life Technologies). We designed custom multiplex reactions according to the manufacturer's instruction and pooled up to five templates per reaction for scalability. As a first step we designed template primers and one extension primer for each variant and performed primer extension on 33 candidates. In order to verify if the detected variant nucleotides were from the mitochondrial or the nuclear genome, we amplified the entire mtDNA in two large fragments of more than 8 kb with an overlap of 183 bp (Voets et al. 2011). These fragments were used as templates in primer extension reactions using the same extension primers used in the first step. Postextension treatment was performed using 1 unit of calf intestinal phosphatase. Electrophoresis was run on an ABI 3730xl DNA Analyzer. Data were analyzed using PeakScanner software (Life Technologies).

## Results

### Molecular analysis of zygosity

Before assessing mitochondrial variation using high-throughput sequencing, we first verified zygosity of the self-reported identical adult twin pair studied. Short tandem repeat analysis was performed on genomic DNA from the blood of the twins. They shared identical alleles at 15 highly variable loci on their chromosomes, indicating monozygosity with high confidence (Table 1).

**Table 1:** Short Tandem Repeat (STR) marker analysis for the twin pair and a DNA control, showing that twin individuals share the same alleles at 16 different loci across the genome confirming their monozygosity at >0.99999 confidence

	Alleles		
	
Locus	Twin A	Twin B	Control
AMEL	XX	XX	XX
CSF1PO	12 12	12 12	10 12
D13S317	10 11	10 11	11 11
D16S539	10 10	10 10	11 12
D18S51	14 21	14 21	15 19
D21S11	31 31	31 31	30 30
D3S1358	17 17	17 17	14 15
D5S818	11 11	11 11	11 11
D7S820	10 11	10 11	10 11
D8S1179	13 13	13 13	13 13
FGA	25 27	25 27	23 24
Penta_D	9 10	9 10	12 12
Penta_E	12 14	12 14	12 13
TH01	9.3 9.3	9.3 9.3	8 9.3
TPOX	11 11	11 11	8 8
vWA	17 17	17 17	17 18

### Mitochondrial sequence analysis by high-throughput sequencing

**Figure 1:**
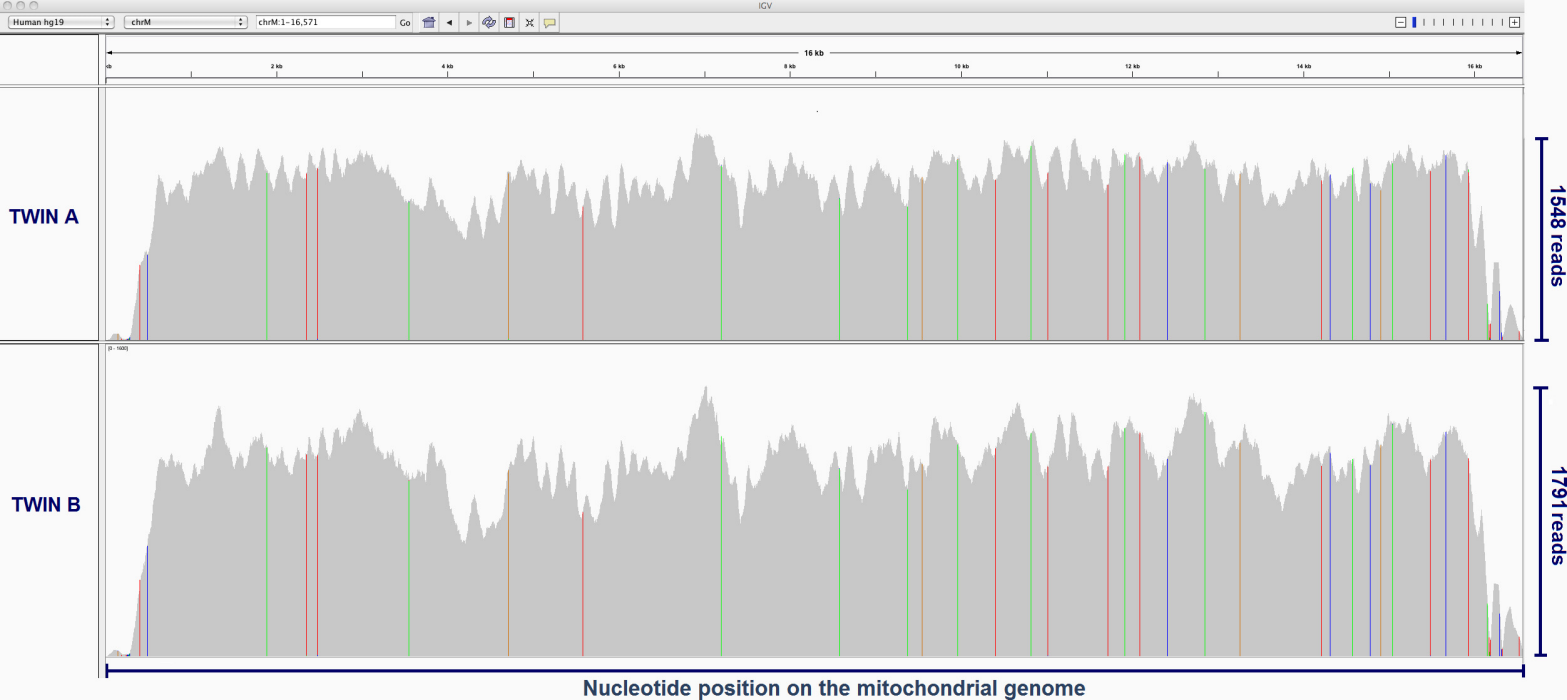
Mitochondrial DNA sequencing coverage and variant map for twin A (upper plot) and twin B (lower plot). The *x*-axis represents the nucleotide position on the mitochondrial genome and the *y*-axis shows the number of reads (depth of coverage) for each nucleotide position (maximum of 1084 reads for twin A and 1395 reads for twin B). Homoplasmic variants (colored vertical lines at specific genomic locations) in both twin A and twin B are concordant. Each variant is colored according to the base type (red for T, green for A, blue for C, and brown for G) compared to the hg19 reference base.

**Figure 2:**
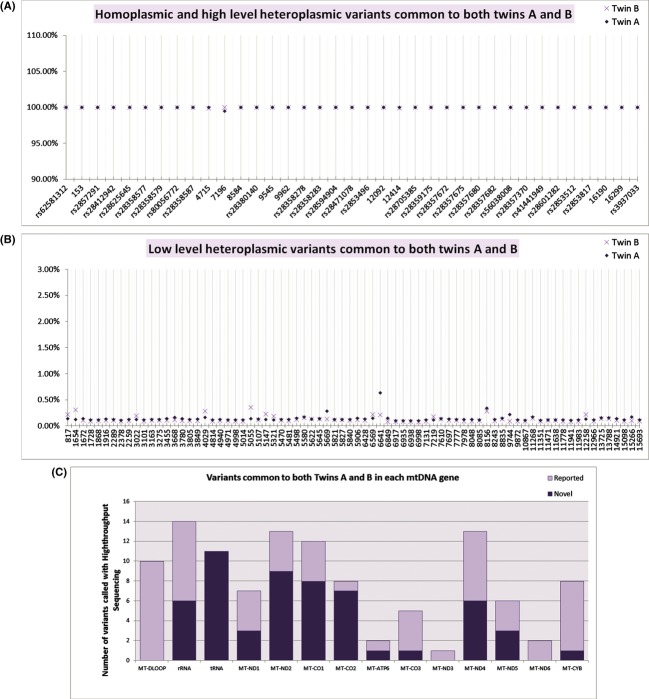
High-throughput called variants common to both twins. (A) Homoplasmic/nearly homoplasmic variants detected for twin A and twin B are concordant. The *y*-axis represents the ratio of variant to reference base. The *x*-axis represents the alignment position of the variant detected. (B) Low-level heteroplasmic variant calls detected in both twin A and twin B. (C) Distribution of novel and reported mitochondrial sequence variants detected in both twins A and B. The *y*-axis represents the number of variants. The *x*-axis represents the mitochondrial genes. Bars represent reported (light) and unreported variants (dark).

To assess twin mitochondrial genome sequencing performance in the context of genome sequencing without prior sequence capture, we prepared paired-end whole genome sequencing libraries and used the Illumina HiSeq platform, which yielded over 100 million reads for each twin individual (Run 1, Table 2). A second sequencing run was performed on a newer version of flow cell (Run 2, Table 2), which greatly increased the number of reads by producing 275 million reads for twin A and over 314 million reads for twin B. We aligned these reads to the hg19 reference genome using Bowtie. Initial alignment using default parameters resulted in alignment of ∼47% of the reads (Alignment 1, Table 2), and this was improved to over 90% of reads aligned (Alignment 2, Table 2) using the “-y/–tryhard” mode, although the alignment performed more slowly. On the mitochondrial genome the mean depth of coverage was 1151 for the twin A and 1279 for the twin B (Fig. 1). We verified variants using the Integrated Genomics Viewer manually and detected 37 high-confidence variants (>99% of the reads), and all these were common to both twin A and B (Fig. 1). These variants included 34 homoplasmic variants and three nearly homoplasmic variants (Fig. 2A). Among these 37 variants, 27 were distributed on 12 genes throughout the mitochondrial genome (Fig. 2C), and 10 were localized at the hypervariable segments HV1 (16024–16383) and HV2 (57–372), which had variable coverage even after remapping to account for the circular mitochondrial genome. Although six variants were nonsynonymous, all were at positions of previously reported mtDNA polymorphism (Table 3).

**Table 2:** Sequencing reads and mapping efficiency

	Twin A		Twin B	
Sequencing run 1: V1 flow cell HiSeq system				
Total number of reads	125,989,576		101,559,160	
Stringency	Alignment 1	Alignment 2	Alignment 1	Alignment 2
Number of mapped reads	59,402,944	113,836,436	47,883,234	91,638,246
Percentage of mapped reads	47.15	90.35	47.15	90.23
Sequencing run 2: V3 flow cell HiSeq system				
Total number of reads	275,033,292		314,499,024	
Stringency	Alignment 1	Alignment 2	Alignment 1	Alignment 2
Number of mapped reads	130,156,770	249,089,720	148,617,794	284,273,752
Percentage of mapped reads	47.32	90.57	47.26	90.39

**Table 3:** Positions and predicted effect of homoplasmic and nearly homoplasmic variants common to both twins A and B (*n* = 37 variants)

mtDNA position	Gene	Reference allele	Variant	Mutation type	First AA	Second AA	Variant identifier	Coverage twin A	Variant ratio twin A (%)	Coverage twin B	Variant ratio twin B (%)
150	MT-DLOOP	T	C	Transversion	NA	NA	rs62581312	30	100	28	100
153	MT-DLOOP	A	G	Transition	NA	NA	RP	34	100	28	100
195	MT-DLOOP	C	T	Transversion	NA	NA	rs2857291	8	100	9	100
408	MT-DLOOP	A	T	Transversion	NA	NA	rs28412942	372	100	382	100
489	MT-DLOOP	T	C	Transversion	NA	NA	rs28625645	440	100	570	100
1888	MT-RNR2	G	A	Transversion	NA	NA	rs28358577	860	100	1070	100
2353	MT-RNR2	C	T	Transversion	NA	NA	rs28358579	854	100	1035	100
2483	MT-RNR2	C	T	Transversion	NA	NA	rs80056772	881	100	1036	100
3552	MT-ND1	T	A	Transversion	A	A	rs28358587	713	100	907	100
4715	MT-ND2	A	G	Transition	G	G	RP	854	100	898	99.9
7196	MT-CO1	C	A	Transversion	L	L	RP	900	99.5	1127	100
8584	MT-ATP6	G	A	Transversion	A	T	rs55728079	733	100	963	100
9377	MT-CO3	G	A	Transition	W	W	rs28380140	684	100	855	100
9545	MT-CO3	A	G	Transition	G	G	RP	803	100	944	100
9962	MT-CO3	G	A	Transition	L	L	RP	929	100	1086	100
10400	MT-ND3	C	T	Transversion	T	T	rs28358278	822	100	1064	100
10819	MT-ND4	G	A	Transition	K	K	rs28358283	952	100	1090	100
11017	MT-ND4	C	T	Transversion	S	S	rs28594904	858	100	975	100
11722	MT-ND4	C	T	Transversion	H	Y	rs28471078	795	100	972	100
11914	MT-ND4	G	A	Transversion	T	T	rs2853496	951	100	1169	100
12092	MT-ND4	C	T	Transversion	L	F	RP	912	100	1097	100
12414	MT-ND5	T	C	Transition	P	P	RP	879	100	965	99.9
12850	MT-ND5	G	A	Transversion	V	I	rs28705385	881	100	1251	100
13263	MT-ND5	A	G	Transition	Q	Q	rs28359175	852	100	1098	100
14212	MT-ND6	C	T	Transversion	V	V	rs28357672	819	100	975	100
14318	MT-ND6	T	C	Transversion	N	S	rs28357675	847	100	1042	100
14783	MT-CYB	T	C	Transversion	L1	L2	rs28357680	804	100	981	100
14905	MT-CYB	A	G	Transversion	M	M	rs28357682	770	100	1084	100
15043	MT-CYB	G	A	Transversion	G	G	rs56038008	873	100	1128	100
15487	MT-CYB	A	T	Transversion	P	P	rs28357370	870	100	1007	100
15930	MT-TT	G	A	Transversion	NA	NA	rs41441949	836	100	947	100
15932	MT-TT	C	C	Transition	NA	NA	rs28601282	859	100	1013	100
16153	MT-DLOOP	G	A	Transversion	NA	NA	rs2853512	189	100	273	100
16172	MT-DLOOP	C	T	Transversion	NA	NA	rs2853817	80	100	100	100
16190	MT-DLOOP	C	T	Transversion	NA	NA	RP	79	100	82	100
16299	MT-DLOOP	T	C	Transversion	NA	NA	RP	251	100	221	100
16520	MT-DLOOP	C	T	Transversion	NA	NA	rs3937033	47	100	101	100

RP, reported polymorphism; AA, First amino acid is reference while Second amino acid is the effect of the variant.

### Low-level variation analysis

We noted the presence of potential single-base sequence differences present at very low level, one to three reads per locus, excluding duplicate reads. As these differences could represent a number of possibilities including sequencing errors, read mapping ambiguity due to numts, or true mitochondrial variation, we investigated these further. In analyzing potential low-level variants in the mitochondrial genome, we used stringent filters: a strict nucleotide quality score (Phred score *Q* ≥ 30), the presence of the variant in at least one sequencing read, and variant surrounded by nonvariant sequence (potential variant is at least five bases from a read end, and lack of other sequence differences in same read).

We detected many single-base candidate low-level variants in the twins (Tables S1, S2, and S3); the presence of more low-level potential variants was correlated with higher coverage. Importantly, we performed additional validation on the original genomic DNA to rule out error introduced by library preparation and complementary studies to evaluate their origin. We focused on 76 low-level variants that were shared between twins (Fig. 2B) given their potentially inherited nature. The low-level variant calls were distributed over the whole mitochondrial genome including the three genes coding for the respiratory chain complex IV and the six genes coding for the respiratory chain complex I (Fig. 2C). Twenty of these variants were previously reported (Mitomap database). From the 56 possibly novel variants, 17 mapped to rRNA or tRNA genes (10 on the RNR2 gene, one on the RNR1 gene, and six distributed on five tRNA genes). The other 39 variants were in coding regions of mitochondrial genes, and 30 were nonsynonymous (Table 4). We designed primers (primer sequences available upon request) to amplify the surrounding regions and primers for fluorescent primer extension for detection of these 30 as they could have functional consequence if confirmed. We also examined two low-level variants called in two different tRNA genes that have been implicated in potential disease, m.12258C>A in diabetes mellitus and Deafness syndrome, and m.3275C>A in Leber's hereditary optic neuropathy (Lynn et al. 1998; Garcia-Lozano et al. 2000). We also tested a variant that was detected in only one twin (twin B, m.4456C>T) for comparison.

**Table 4:** Novel and nonsynonymous variants common to both twins A and B (*n* = 30 variants)

mtDNA position	Gene	Major allele	Twin A					Twin B				
	
			Minor allele	Minor allele ratio (%)	First AA	Second AA	Mutation type	Minor allele	Minor allele ratio (%)	First AA	Second AA	Mutation type
3455	MT-ND1	C	T	0.11	A	V	Transition	A	0.13	A	D	Transversion
3668	MT-ND1	G	A	0.12	W	STOP	Transition	A	0.16	W	STOP	Transition
3805	MT-ND1	A	G	0.10	T	A	Transition	T	0.12	T	S	Transversion
4971	MT-ND2	G	T	0.10	G	C	Transversion	T	0.12	G	C	Transversion
4998	MT-ND2	A	T	0.09	K	STOP	Transversion	G	0.12	K	E	Transition
5014	MT-ND2	C	G	0.09	S	C	Transversion	A	0.12	S	Y	Transversion
5055	MT-ND2	T	C	0.35	Y	H	Transition	C	0.13	Y	H	Transition
5107	MT-ND2	C	T	0.12	T	I	Transition	A	0.13	T	N	Transversion
5470	MT-ND2	C	T	0.11	H	M	Transition	A	0.12	T	K	Transversion
5481	MT-ND2	C	A	0.10	P	T	Transversion	A	0.12	P	T	Transversion
5906	MT-CO1	G	A	0.11	M	M	Transition	T	0.14	M	M	Transition
6569	MT-CO1	C	A	0.22	P	P	Transversion	A	0.14	R	S	Transversion
6849	MT-CO1	A	C	0.08	T	P	Transversion	T	0.14	T	S	Transversion
6935	MT-CO1	C	T	0.08	H	Y	Transition	T/A	0.10	H	N	Transversion
6998	MT-CO1	C	A	0.08	I	M	Transversion	A	0.10	I	M	Transversion
7131	MT-CO1	G	T	0.09	A	S	Transition	T	0.11	A	S	Transition
7219	MT-CO1	G	T	0.18	R	L	Transversion	T	0.11	R	L	Transversion
7610	MT-CO2	C	A	0.13	L	M	Transversion	A	0.14	L	M	Transversion
7978	MT-CO2	G	T	0.09	G	V	Transition	T	0.12	G	V	Transition
8048	MT-CO2	A	T	0.09	T	S	Transversion	G	0.12	T	A	Transversion
8085	MT-CO2	C	G	0.10	T	STOP	Transversion	A	0.12	T	K	Transversion
8156	MT-CO2	G	C	0.28	V	L	Transversion	C	0.33	V	L	Transversion
8243	MT-CO2	G	T	0.10	E	STOP	Transversion	A/T	0.12	E	K/STOP	Transition
9744	MT-CO3	G	T	0.09	E	STOP	Transversion	T	0.22	E	STOP	Transversion
10867	MT-ND4	C	A	0.09	I	M	Transversion	G	0.11	I	M	Transversion
11351	MT-ND4	G	T	0.09	A	S	Transversion	T	0.11	A	S	Transversion
11638	MT-ND4	C	T	0.11	H	Y	Transition	A	0.11	T	N	Transversion
11941	MT-ND4	T	A	0.08	L	Q	Transversion	G	0.10	L	Q	Transversion
13725	MT-ND5	C	A	0.14	F	L	Transversion	A	0.15	F	L	Transversion
15266	MT-CYB	A	C	0.10	T	P	Transversion	G	0.17	T	A	Transversion

AA, amino acid effect of variant.

**Figure 3:**
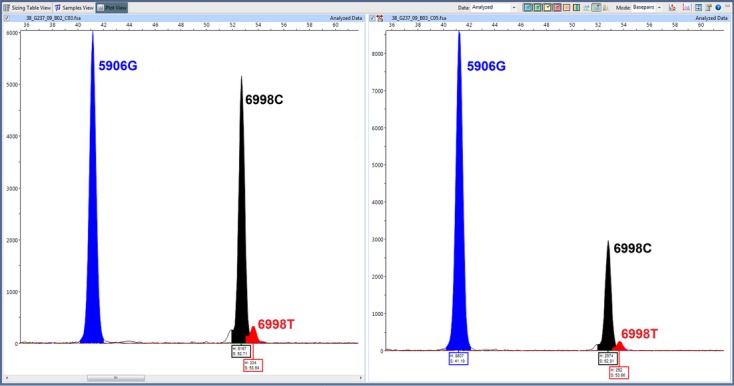
Electropherograms showing primer extension assay for two positions from either twin A (left) or twin B (right). The blue and black peaks correspond to the major alleles (G at position 9606, C at 6998), while the small red peaks indicate the presence of a T allele at position 6998 at a very low level.

Using the original genomic DNA, we found the major reference allele in 33/33 positions, which confirms the results from the high-throughput sequencing, and the low-level variant was seen in 9/33 (27.3%) (Table 5). An example is shown in Figure 3. In all cases, primer extension revealed concordant results between the twin individuals with or without variant sequence. Where low-level variants were not confirmed, these could represent sequencing false positives or variant detection could be limited by sensitivity even though the seven primer extension assays detected variants seen in <1% of high-throughput sequencing reads.

**Table 5:** Comparison of SNP detection using the HiSeq and the primer extension techniques

mtDNA position	Gene	Primer extension		HiSeq	HiSeq minor allele twin A				HiSeq minor allele twin B			
		
		Major allele	Minor allele	Major allele	Run2 HS	Run2 MS	Run1 HS	Run1 MS	Run2 HS	Run2 MS	Run1 HS	Run1 MS
3275	MT-TL1	**C**	–	C	T	A	–	–	G	G	–	–
3455	MT-ND1	C	A	C	A	–	–	–	T	T	–	–
3668	MT-ND1	G	–	G	A	A	–	–	A	A	–	–
3805	MT-ND1	A	–	A	T	T	–	–	G	G	–	–
4456	MT-TM	C	T	C	C	T	–	–	T	–	–	–
4971	MT-ND2	G	–	G	T	T	–	–	T	T	–	–
4998	MT-ND2	A	C	A	G	–	C	–	T	T	C	–
5014	MT-ND2	C	A	C	A	A	A	–	G	A	–	–
5055	MT-ND2	T	–	T	C	C	–	–	C	C	–	–
5107	MT-ND2	C	–	C	A	A	–	–	T	A	–	–
5470	MT-ND2	C	–	C	A	–	–	–	T	–	–	–
5481	MT-ND2	C	–	C	A	–	–	–	–	A	–	A
5906	MT-CO1	G	–	G	T	T	–	–	A	–	T	T
6569	MT-CO2	C	A	C	A	A	–	–	A	A	–	–
6849	MT-CO1	A	–	A	T	T/C	C	C	C	T	C	C
6935	MT-CO1	C	A	C	T/A	T	T	–	T	T	T	–
6998	MT-CO1	C	T	C	A	A	–	T	A	–	–	–
7131	MT-CO1	G	–	G	T	T	–	–	T	T	–	–
7219	MT-CO1	G	–	G	T	T	A	A	T	T	T	T
7610	MT-CO2	C	–	C	A	–	T	T	A	–	–	–
7978	MT-CO2	G	C	G	T	C	–	–	T	C	–	–
8048	MT-CO2	A	–	A	G	T	–	–	T	T	–	–
8085	MT-CO2	C	–	C	A	A	–	–	G	–	–	–
8156	MT-CO2	G	–	G	C	C	C	C	C	C	C	C
8243	MT-CO2	G	–	G	A/T	T	–	–	T	T	T/C	T/C
9744	MT-CO3	G	–	G	T	T	T	T	T	T	–	–
10867	MT-ND4	C		C	G	–	A	–	A	–	–	–
12258	MT-TS2	C	–	C	A	–	A	A	A	A	–	–
11351	MT-ND4	G	–	G	T	T	C	–	T	T	–	T
11638	MT-ND4	C	–	C	A	A	A	–	T	T	T	T
11941	MT-ND4	T	A	T	G	–	G	G	A	A	–	–
13725	MT-ND5	C	–	C	A	–	–	–	A	–	A	–
15266	MT-CYB	A	–	A	G	G	G	G	C	C	C	C

HiSeq, high-throughput sequencing; MS, aligned with moderate stringency; HS, aligned with high stringency. Dash indicates minor allele was not detected.

**Figure 4:**
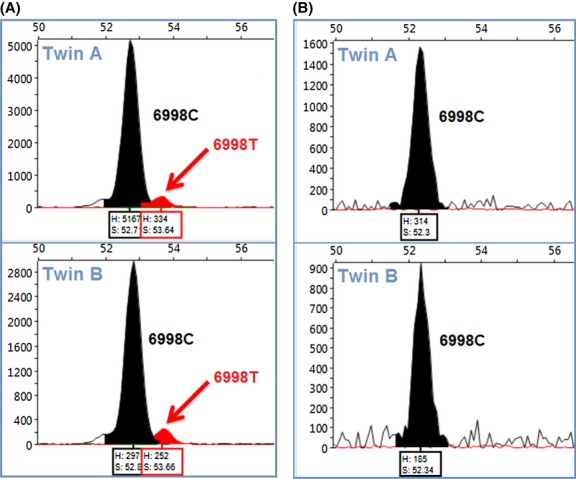
Electropherograms showing the genotype detected in each twin by primer extension on genomic DNA (A) or mitochondrial hemigenome templates (B). The black peak corresponds to the reference allele “C” at position 6998 while the red peak indicates the presence of a “T” allele at a low level. The low-level variant detected using primer extension from genomic DNA (B) was absent in assay with the mitochondrial hemigenome (B) suggesting the T allele signal may result from an extramitochondrial source (numt).

Given these findings, for the seven variants confirmed by primer extension, we considered the possibility that nuclear mtDNA fragments (numts) could be generating apparent mitochondrial sequence variants. To test this possibility, we generated hemigenomes of mtDNA using high-fidelity long PCR and used these as templates for the primer extension assays, as this would eliminate numts (Li et al. 2012). Accordingly, we did not find low-level variants from the preamplified mitochondrial hemigenomes (Fig. 4). The absence of low-level variant detection from the hemigenomes could suggest that they are not present in the mtDNA, but we cannot exclude the possibility that these variants are present in the mtDNA at such a low level that they were not amplified in the hemigenome reaction.

In order to determine if variants reflected numt sequences (from other regions of the genome), we directly compared variant-containing reads to the genome (Data S1). We found homologies to regions of chromosomes 1 and 17, where numts are known to exist. Reads containing position 4457 or 6569, for example, have complete read homology to corresponding regions on chromosome 1 and the primer extension could coamplify mitochondrial and nuclear DNA, explaining the apparent variation in those positions, whereas primer extension on mitochondrial hemigenome only detected the mitochondrial genome. There were, however, several low-level variants, 3455 for example, where sequencing read and primer extension assay precise homology to the nuclear genome were both not present, suggesting these variants may be present in mtDNA albeit at low levels that may not be detectable by analysis of long mitochondrial fragments.

**Figure 5:**
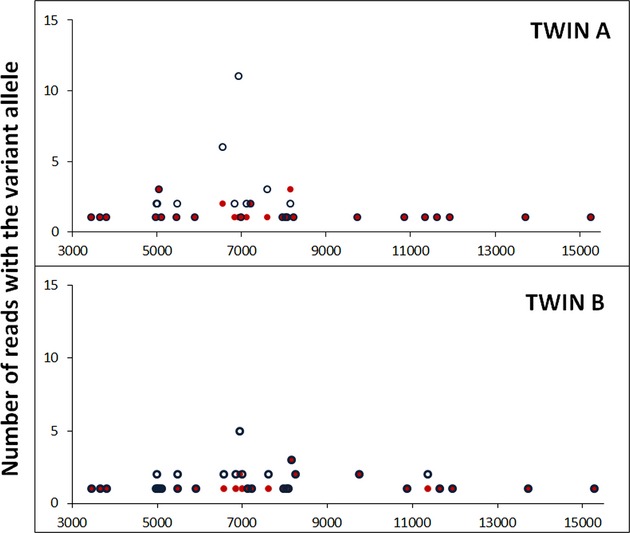
Mapping reads to test for nuclear mitochondrial sequences. Resulting low-level variant reads for twins when reads are mapped to the hg 19 reference genome (red dots) or to the mtDNA genome only (blue circles). The *y*-axis represents the number of reads with the variant allele for each position on the mitochondrial sequence, *x*-axis.

To detect numts that could be mapping to the mitochondrial genome and producing low-level apparent variants, we performed read mapping using whole genome reads from the twins to the mtDNA only as reference. Depth of coverage increased up to 600 reads higher than the initial mapping using the whole hg19 genomic reference, and almost all of these increased the only allele or major allele call. We examined the low-level variants previously analyzed using the initial mapping data and the primer extension assay in table 5. Many of low-level variants did not show any increase in reads and these could be due to errors. However, the number of variant reads increased in 7 of 33 positions in twin A and B (Fig. 5). This increase is likely due to the presence of numts that now were solely mapped to the mtDNA. Some of these variants were present in reads that show high similarity with regions in the assembled nuclear genome. For example, reads with the variant at position 6569 are highly similar to chr1:567077-567211 (Data S1). The number of reads containing the variant allele at this specific position increased for both twins after read mapping to mtDNA alone, suggesting that this variant is due to a numt.

These findings support mitochondrial similarity in twins and demonstrate the importance of distinguishing mitochondrial genome from homologous nuclear DNA using a combination of methods.

## Discussion

In this study, we analyzed mitochondrial sequence variants in a pair of adult twins using high-throughput sequencing and validation. These data also allowed us to ask whether these two independent individuals harbored mitochondrial somatic variation in their blood. Our results revealed identical homoplasmic variants in mtDNA sequence from blood-derived genomic DNA, which lend support to the idea that monozygotic twins may be highly similar in their mitochondrial genomic sequence, as they seem to be in nuclear genomic sequence (Baranzini et al. 2010; Jakobsen et al. 2011). The presence of shared variants in twins suggests that the variants were likely present since conception (Avital et al. 2012). As mtDNA variation impacts many biological processes and can affect disease, it presents important challenges to diagnostic capabilities and could inform us about mutation and selection. Any intertwin differences in variant accumulation may depend on individuals studied, tissues examined or on age of subjects.

Heteroplasmy may change with age due to the accumulation of mtDNA variation Wallace 2001 Krishnan et al. 2007; Larsson 2010), Lee and Wei 2012; (Bratic and Larsson 2013, but this may depend on several factors. Mitochondrial heteroplasmy in blood samples was increased in older aged individuals, with no obvious mutation type pattern (Sondheimer et al. 2011). Targeted studies of the mtDNA have demonstrated variation in grandmothers (do Rosário Marinho et al. 2011). Interestingly, studies that examined murine liver did not detect differences in mutation load with age (Ameur et al. 2011). In our analysis of two adult twin blood samples, we did not find accumulated mtDNA mutation above the 1% level. In addition, as our twin pair were young adults, it will be interesting to examine variants at different ages and environments. If numt and sequencing errors can be excluded, these low-level variants could possibly result from replication errors.

Our finding also revealed that low-level variation detected in the mitochondrial sequence from whole genome high-throughput sequencing can reflect homologous mitochondrial sequences. Low-level variants could represent sequencing errors, low frequency variation, numts, or a combination of these possibilities. The low-read calls considered in our studies only represented the top ∼0.5% of nucleotide quality score. Only with secondary sequencing assays do we see that the variants may not present in mitochondrial genome-enriched templates. Although we verified some of these nucleotides using analysis of original genomic DNA samples, some of these low-level called variants in mitochondrial sequence could not be detected by a secondary method. These variants could represent sequencing error or could reflect differences in sensitivity of primer extension. High-throughput sequencing errors that can be due to the sequencing signal dephasing that occur during the sequencing cycling (Metzker 2010) and might be due to DNA back folding (Allhoff et al. 2013). Additional studies are needed to develop algorithms to filter these out prior to variant call.

The detection of possible numts in our sequencing studies provides insights into the dynamic nature of the mitochondrial sequence over evolutionary time; however, it also poses challenges in mitochondrial genome interpretation. Our findings support that initial mtDNA enrichment can identify mtDNA signals separately from potential numts, and techniques such as primer extension may be considered as an adjunct to high-throughput sequencing.

In our analyses, we found that many of the low-level variants detected by sequencing, even those mapped with high homology to the mitochondrial sequence, were not detected by primer extension using mtDNA hemigenomes. In addition, the mapping parameters could affect the low-level variant calls, and these factors could potentially influence the rate of false positives. If low-level mtDNA variants exist, they may be present at very low levels of heteroplasmy (Payne et al. 2012). Although low-level variation may not substantially impact the interpretation of clinical laboratory results, they may be helpful for understanding variation origin and could exist at different frequencies in different tissues (Payne et al. 2012). Given selective pressure on a specific gene, these low-level variants could potentially accumulate over time. While low-level variants resulting from sequencing may represent mapping ambiguity or result from presequence processing of DNA, identification of the exact same variants in two adult monozygotic twins by primer extension from the original DNA samples suggests these variants could exist. If true variants arise that differ between monozygotic twins, these could be somatic mutations that accumulate due to the low replication fidelity of the mtDNA polymerase (Lee and Johnson 2006). While the probability of the human mtDNA polymerase to misincorporate bases is estimated to be ∼5.6 × 10^−7^ to 2.8 × 10^−8^ errors per incorporated base (Lee and Johnson 2006), overall somatic mutation rates in mitochondria are not well defined. Mitochondrial mutation rates using population and phylogenetic data have found ∼1.66 × 10^−8^ ± 1.48 × 10^−9^ substitutions per nucleotide per year (Soares et al. 2009). In our studies of adult twins, we found that the mitochondrial sequence was largely identical. For the variants that appeared different between twins, we calculated 1.2–1.6 × 10^−6^ events observed per site per year. If 1–1.4% of these calls were somatic events, the observed rate per individual would approximate the predicted error rate. A recent study reported an intratwin pair difference in monozygotic twins suggesting a posttwinning de novo copy number variant event (Ehli et al. 2012). Several other studies have reported CNVs in twin studies (Bruder et al. 2008; Maiti et al. 2011; Sasaki et al. 2011; Breckpot et al. 2012), and so there may be differences in somatic Copy Number Variant and SNP occurrence. Further studies should address the possibility of somatic events and selection.

Current read mapping to the genome insufficiently accounts for the homology between numts and the mtDNA. Variants from whole genome sequencing may be found in the mitochondrial genome or the corresponding numt, which could alter estimates of heteroplasmy. This is an important consideration in assays that measure mtDNA heteroplasmy especially for mitochondrial disorder diagnosis, where a threshold of variation may need to be surpassed to express disease. Our findings are salient given the increased application of genome sequencing and efforts to identify mosaic variation and to distinguish regions of genome homology. We suggest that combined methods of high-throughput sequencing, techniques like primer extension, and mtDNA enrichment may be useful in assessing mtDNA variants and numts.

Finally, we conclude the following: (1) these adult twins had highly similar mtDNA sequence from blood. (2) We did not find differential somatic heteroplasmy >1% to suggest accumulating mutation over time, although more twin pairs and tissues should be tested in the future. (3) Low-level variants, only some of which are numts, are detected by high-throughput sequencing and can be confirmed by primer extension.

## Supporting Information

**Data S1.** Summary of Blast, BLAT, and nuclear mitochondrial sequences (numts) analysis applied to the reads and extension PCR template fragment of eight variants.

**Table S1.** List of annotated Low level variants for Twin A.

**Table S2.** List of annotated Low level variants for Twin B.

**Table S3.** Sequencing SNP calling by Phred quality score.
