# Supplementary material for: Twin mitochondrial sequence analysis
Source: Mol Genet Genomic Med. 2013 Jun 26;1(3):174–86. doi: 10.1002/mgg3.20 (PMC3768015; doi:10.1002/mgg3.20)

| **Variant** | **Amplified Fragment Size** | **Similar fragments** | **Number of Mismatches** | **Fragments amplified with the same primers** | **Number of Primers Mismatches** | **Fragments Similar to the Read** | **Number of Mismatches** | **Numts at the Variant Position** |
| --- | --- | --- | --- | --- | --- | --- | --- | --- |
| m.3455 C>T/A | 215 | 0 | 0 | 0 | 0 | chr11:103278929-103279022 | 9 | 4T, 1G,26C |
|  |  |  |  |  |  | chr17:19505654-19505745 | 10 |  |
| m.4457C>T | 232 | chr1:564900-565131 | 1 | chr1 NT_004350.19 | 0F 0R | chr1:564966-565066 | 0 | 1T 1G 49C |
|  |  | chr17:22025105-22025336 | 30 | chr3 NT_005612.16 | 2F 2R | chr17:22025171-22025256 | 5 |  |
|  |  | chr2:140977828-140978055 | 40 |  |  | chr1:564921-565021 | 0 |  |
|  |  | chr17:19506612-19506828 | 62 |  |  |  |  |  |
| m.4998 A>T/G | 230 | chr1:565469-565698 | 3 | Chr9 NT_008470.19 | 0F 3R | chr1:565486-565585 | 4 | 35 A |
|  |  |  |  | chr8 NT_023678.16 | 1F 2R | chr1:565509-565604 | 3 |  |
|  |  |  |  | chr4 NT_016354.19 | 0F 4R | chr17:22025720-22025796 | 9 |  |
|  |  |  |  | chrX NT_011630.14 | 1F 3R |  |  |  |
|  |  |  |  | chr7 NT_007914.15 | 1F 3R |  |  |  |
|  |  |  |  | chr4 NT_006316.16 | 1F 3R |  |  |  |
|  |  |  |  | chr7 NT_007933.15 | 1F 4R |  |  |  |
|  |  |  |  | chr2 NT_022135.16 | 2F 3R |  |  |  |
|  |  |  |  | chr1 NT_167186.1 | 1F 4R |  |  |  |
| m.5014C>G/A | 230 | chr1:565469-565698 | 5 | 0 | 0 | chr1:565539-565639 | 4 | 34 C |
| m.5906 G>A/T | 178 | chr1:566365-566542 | 2 | chr1 NT_004350.19 | 1F 0R | chr1:566413-566513 | 1 | 2A 35G |
|  |  | chr14:32953918-32954094 | 11 | ch14 NT_026437.12 | 1F 1R | chr14:32953790-32954046 | 6 |  |
|  |  |  |  | chr17 NT_010783.15 | 2F 2R | chr1:566387-566487 | 2 |  |
|  |  |  |  | chr2 NT_05403.17 | 2F 2R | chr14:32953973-32954065 | 6 |  |
| m.6569C>A | 249 | chr1:566927-567175 | 7 | chr1 NT_004350.19 | 1F 0R | chr1:567111-567211 | 0 | 7G 14T 13A |
|  |  |  |  | chr9 NT_008470.19 | 3F 2R | chrX:125605695-125605795 | 8 |  |
|  |  |  |  | chr4 NT_016354.19 | 3F 3R | chr1:567077-567177 | 0 |  |
|  |  |  |  | chr7 NT_007914.15 | 3F 3R |  |  |  |
| m.6998C>A | 232 | chr1:567401-567632 | 2 | chr 1NT_004350.19 | 0F 0R | chr1:567460-567560 | 3 | 23T 7A |
|  |  | chr17:51183126-51183357 | 13 | chr17 NT_010783.15 | 0F 2R | chr17:51183185-51183285 | 8 |  |
|  |  | chrX:125605984-125606215 | 21 | chrX NT_011786.16 | 1F 3R | chr2:49456770-49456870 | 9 |  |
|  |  | chr5:99389754-99389979 | 23 | chr2 NT_034772.6 | 2F 3R | chr1:567467-567567 | 3 |  |
|  |  |  |  | chr10 NT_030059.13 | 3F 2R | chr17:51183192-51183292 | 8 |  |
|  |  |  |  | chr7 NT_007933.15 | 2F 3R |  |  |  |
|  |  |  |  | chr2 NT_022135.16 | 3F 2R |  |  |  |
|  |  |  |  | ch8 NT_008046.16 | 4F 2R |  |  |  |
| m.12258 C>A | 152 | chr5:134262153-134262304 | 7 | chr5 NT_034772.6 | 1F 0R  3F 0R | chr5:134262145-134262236 | 3 | 3T 1G 35A |
|  |  | chr5:99384493-99384644 | 9 |  |  | chr4:163342602-163342693 | 3 |  |
|  |  |  |  |  |  | chr5:99384485-99384576 | 4 |  |
|  |  |  |  |  |  | chr10:36723409-36723499 | 8 |  |
|  |  |  |  |  |  | chr2:202077807-202077897 | 9 |  |
|  |  |  |  |  |  | chr5:134262214-134262314 | 7 |  |
|  |  |  |  |  |  | chr5:99384554-99384654 | 9 |  |
|  |  |  |  |  |  | chr12:130800216-130800291 | 7 |  |

**Summary of Blast, BLAT and Numts analysis applied to the reads and extension PCR template fragment of 8 variants**

**Blast, BLAT and Numts analysis applied to the reads and extension PCR template fragment of 8 variants**

**Variant1: m.3455 C>T/A 215pb**

**Template Fragment** (Primers are represented in bold, variant in red and the extension primer is underlined)

**ATGGCCAACCTCCTACTCCT**CATTGTACCCATTCTAATCGCAATGGCATTCCTAATGCTTACCGAACGAAAAATTCTAGGCTATATACAACTACGCAAAGGCCCCAACGTTGTAGGCCCCTACGGGCTACTACAACCCTTCG**C**TGACGCCATAAAACTCTTCACCAAAGAGCCCCTAAAACCCGCCACATCTAC**CATCACCCTTTATATTATTG**

**Template BLAT results**

ACTIONS QUERY SCORE START END QSIZE IDENTITY CHRO STRAND START END SPAN

---------------------------------------------------------------------------------------------------

[browser](http://genome.ucsc.edu/cgi-bin/hgTracks?position=chrM:3314-3528&db=hg19&ss=../trash/hgSs/hgSs_genome_53b7_747bd0.pslx+../trash/hgSs/hgSs_genome_53b7_747bd0.fa&hgsid=256713665) [details](http://genome.ucsc.edu/cgi-bin/hgc?o=3313&g=htcUserAli&i=../trash/hgSs/hgSs_genome_53b7_747bd0.pslx+..%2Ftrash%2FhgSs%2FhgSs_genome_53b7_747bd0.fa+YourSeq&c=chrM&l=3313&r=3528&db=hg19&hgsid=256713665) YourSeq 215 1 215 215 100.0% M + 3314 3528 215

[browser](http://genome.ucsc.edu/cgi-bin/hgTracks?position=chr17:19505654-19505745&db=hg19&ss=../trash/hgSs/hgSs_genome_53b7_747bd0.pslx+../trash/hgSs/hgSs_genome_53b7_747bd0.fa&hgsid=256713665) [details](http://genome.ucsc.edu/cgi-bin/hgc?o=19505653&g=htcUserAli&i=../trash/hgSs/hgSs_genome_53b7_747bd0.pslx+..%2Ftrash%2FhgSs%2FhgSs_genome_53b7_747bd0.fa+YourSeq&c=chr17&l=19505653&r=19505745&db=hg19&hgsid=256713665) YourSeq 63 77 170 215 80.5% 17 + 19505654 19505745 92

[browser](http://genome.ucsc.edu/cgi-bin/hgTracks?position=chr6:156868970-156869037&db=hg19&ss=../trash/hgSs/hgSs_genome_53b7_747bd0.pslx+../trash/hgSs/hgSs_genome_53b7_747bd0.fa&hgsid=256713665) [details](http://genome.ucsc.edu/cgi-bin/hgc?o=156868969&g=htcUserAli&i=../trash/hgSs/hgSs_genome_53b7_747bd0.pslx+..%2Ftrash%2FhgSs%2FhgSs_genome_53b7_747bd0.fa+YourSeq&c=chr6&l=156868969&r=156869037&db=hg19&hgsid=256713665) YourSeq 52 1 68 215 88.3% 6 - 156868970 156869037 68

[browser](http://genome.ucsc.edu/cgi-bin/hgTracks?position=chr7:141504344-141504412&db=hg19&ss=../trash/hgSs/hgSs_genome_53b7_747bd0.pslx+../trash/hgSs/hgSs_genome_53b7_747bd0.fa&hgsid=256713665) [details](http://genome.ucsc.edu/cgi-bin/hgc?o=141504343&g=htcUserAli&i=../trash/hgSs/hgSs_genome_53b7_747bd0.pslx+..%2Ftrash%2FhgSs%2FhgSs_genome_53b7_747bd0.fa+YourSeq&c=chr7&l=141504343&r=141504412&db=hg19&hgsid=256713665) YourSeq 45 33 101 215 82.7% 7 - 141504344 141504412 69

[browser](http://genome.ucsc.edu/cgi-bin/hgTracks?position=chr4:93623566-93623620&db=hg19&ss=../trash/hgSs/hgSs_genome_53b7_747bd0.pslx+../trash/hgSs/hgSs_genome_53b7_747bd0.fa&hgsid=256713665) [details](http://genome.ucsc.edu/cgi-bin/hgc?o=93623565&g=htcUserAli&i=../trash/hgSs/hgSs_genome_53b7_747bd0.pslx+..%2Ftrash%2FhgSs%2FhgSs_genome_53b7_747bd0.fa+YourSeq&c=chr4&l=93623565&r=93623620&db=hg19&hgsid=256713665) YourSeq 41 68 122 215 87.3% 4 + 93623566 93623620 55

[browser](http://genome.ucsc.edu/cgi-bin/hgTracks?position=chr11:103278998-103279053&db=hg19&ss=../trash/hgSs/hgSs_genome_53b7_747bd0.pslx+../trash/hgSs/hgSs_genome_53b7_747bd0.fa&hgsid=256713665) [details](http://genome.ucsc.edu/cgi-bin/hgc?o=103278997&g=htcUserAli&i=../trash/hgSs/hgSs_genome_53b7_747bd0.pslx+..%2Ftrash%2FhgSs%2FhgSs_genome_53b7_747bd0.fa+YourSeq&c=chr11&l=103278997&r=103279053&db=hg19&hgsid=256713665) YourSeq 36 46 101 215 82.2% 11 - 103278998 103279053 56

[browser](http://genome.ucsc.edu/cgi-bin/hgTracks?position=chr2:117781564-117781600&db=hg19&ss=../trash/hgSs/hgSs_genome_53b7_747bd0.pslx+../trash/hgSs/hgSs_genome_53b7_747bd0.fa&hgsid=256713665) [details](http://genome.ucsc.edu/cgi-bin/hgc?o=117781563&g=htcUserAli&i=../trash/hgSs/hgSs_genome_53b7_747bd0.pslx+..%2Ftrash%2FhgSs%2FhgSs_genome_53b7_747bd0.fa+YourSeq&c=chr2&l=117781563&r=117781600&db=hg19&hgsid=256713665) YourSeq 31 65 101 215 91.9% 2 + 117781564 117781600 37

[browser](http://genome.ucsc.edu/cgi-bin/hgTracks?position=chr8:47739932-47739969&db=hg19&ss=../trash/hgSs/hgSs_genome_53b7_747bd0.pslx+../trash/hgSs/hgSs_genome_53b7_747bd0.fa&hgsid=256713665) [details](http://genome.ucsc.edu/cgi-bin/hgc?o=47739931&g=htcUserAli&i=../trash/hgSs/hgSs_genome_53b7_747bd0.pslx+..%2Ftrash%2FhgSs%2FhgSs_genome_53b7_747bd0.fa+YourSeq&c=chr8&l=47739931&r=47739969&db=hg19&hgsid=256713665) YourSeq 30 64 101 215 89.5% 8 - 47739932 47739969 38

[browser](http://genome.ucsc.edu/cgi-bin/hgTracks?position=chr2:140978948-140978981&db=hg19&ss=../trash/hgSs/hgSs_genome_53b7_747bd0.pslx+../trash/hgSs/hgSs_genome_53b7_747bd0.fa&hgsid=256713665) [details](http://genome.ucsc.edu/cgi-bin/hgc?o=140978947&g=htcUserAli&i=../trash/hgSs/hgSs_genome_53b7_747bd0.pslx+..%2Ftrash%2FhgSs%2FhgSs_genome_53b7_747bd0.fa+YourSeq&c=chr2&l=140978947&r=140978981&db=hg19&hgsid=256713665) YourSeq 30 68 101 215 94.2% 2 - 140978948 140978981 34

[browser](http://genome.ucsc.edu/cgi-bin/hgTracks?position=chr8:32871673-32871705&db=hg19&ss=../trash/hgSs/hgSs_genome_53b7_747bd0.pslx+../trash/hgSs/hgSs_genome_53b7_747bd0.fa&hgsid=256713665) [details](http://genome.ucsc.edu/cgi-bin/hgc?o=32871672&g=htcUserAli&i=../trash/hgSs/hgSs_genome_53b7_747bd0.pslx+..%2Ftrash%2FhgSs%2FhgSs_genome_53b7_747bd0.fa+YourSeq&c=chr8&l=32871672&r=32871705&db=hg19&hgsid=256713665) YourSeq 27 69 101 215 91.0% 8 + 32871673 32871705 33

[browser](http://genome.ucsc.edu/cgi-bin/hgTracks?position=chr11:89669140-89669163&db=hg19&ss=../trash/hgSs/hgSs_genome_53b7_747bd0.pslx+../trash/hgSs/hgSs_genome_53b7_747bd0.fa&hgsid=256713665) [details](http://genome.ucsc.edu/cgi-bin/hgc?o=89669139&g=htcUserAli&i=../trash/hgSs/hgSs_genome_53b7_747bd0.pslx+..%2Ftrash%2FhgSs%2FhgSs_genome_53b7_747bd0.fa+YourSeq&c=chr11&l=89669139&r=89669163&db=hg19&hgsid=256713665) YourSeq 22 78 101 215 95.9% 11 - 89669140 89669163 24

[browser](http://genome.ucsc.edu/cgi-bin/hgTracks?position=chr1:217116661-217116684&db=hg19&ss=../trash/hgSs/hgSs_genome_53b7_747bd0.pslx+../trash/hgSs/hgSs_genome_53b7_747bd0.fa&hgsid=256713665) [details](http://genome.ucsc.edu/cgi-bin/hgc?o=217116660&g=htcUserAli&i=../trash/hgSs/hgSs_genome_53b7_747bd0.pslx+..%2Ftrash%2FhgSs%2FhgSs_genome_53b7_747bd0.fa+YourSeq&c=chr1&l=217116660&r=217116684&db=hg19&hgsid=256713665) YourSeq 22 191 213 215 100.0% 1 + 217116661 217116684 24

[browser](http://genome.ucsc.edu/cgi-bin/hgTracks?position=chrX:17134718-17134737&db=hg19&ss=../trash/hgSs/hgSs_genome_53b7_747bd0.pslx+../trash/hgSs/hgSs_genome_53b7_747bd0.fa&hgsid=256713665) [details](http://genome.ucsc.edu/cgi-bin/hgc?o=17134717&g=htcUserAli&i=../trash/hgSs/hgSs_genome_53b7_747bd0.pslx+..%2Ftrash%2FhgSs%2FhgSs_genome_53b7_747bd0.fa+YourSeq&c=chrX&l=17134717&r=17134737&db=hg19&hgsid=256713665) YourSeq 20 68 87 215 100.0% X - 17134718 17134737 20

**Reads BLAT results**

7: chrM:3383-3483 (-)

AAAATTCTAGGCTATATACAACTACGCAAAGGCCCCAACGTTGTAGGCCCCTACGGGCTACTACAACCCTTCG**A**TGACGCCATAAAACTCTTCACCAAAGA

## chr11:103278929-103279022

TAGGCTATATACAACTACGCAAAGGatCtAACacTGTAGGCtCCTACGGaCTgCTtCAACCaTTtG**c**TGAtGCaATAAAACTtTTCACCAAAGAaccctt

## chr17:19505654-19505745

TAGGCTATATACAACTACGCAAAGGaCCtgACaTTGTAGGtCCCTAtGGaCTgCTtCAACCaTTCa**c**TGAtGCagTAAAACTTCACCAAAGA

8: chrM:3377-3477 (+)

GAACGAAAAATTCTAGGCTATATACAACTACGCAAAGGCCCCAACGTTGTAGGCCCCTACGGGCTACTACAACCCTTCG**T**TGACGCCATAAAACTCTTCAC

**Numts aligned to the variant on the UCSC genome browser**


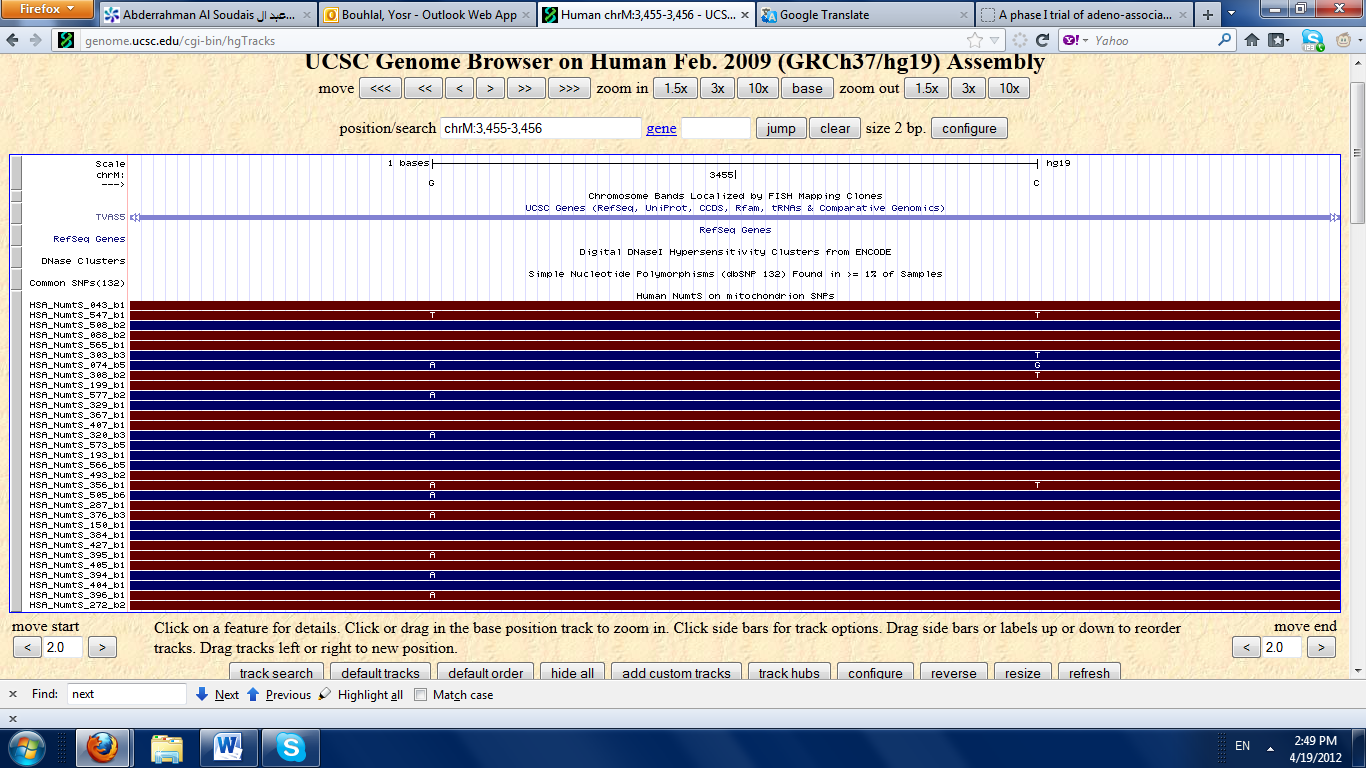


Numts : 31 numts 4T 1G

**Variant2 : m.4457C>T**

**Template Fragment** **CCATCCCTGAGAATCCAAAA**TTCTCCGTGCCACCTATCACACCCCATCCTAAAGTAAGGTCAGCTAAATAAGCTATCGGGCCCATACCCCGAAAATGTTGGTTATA**C**CCTTCCCGTACTAATTAATCCCCTGGCCCAACCCGTCATCTACTCTACCATCTTTGCAGGCACACTCATCACAGCGCTAAGCTCGCACTGATTTTTTACCT**GAGTAGGCCTAGAAATAAACATG**

**Template BLAT results**

ACTIONS QUERY SCORE START END QSIZE IDENTITY CHRO STRAND START END SPAN

---------------------------------------------------------------------------------------------------

[browser](http://genome.ucsc.edu/cgi-bin/hgTracks?position=chrM:4351-4582&db=hg19&ss=../trash/hgSs/hgSs_genome_2f5f_daef20.pslx+../trash/hgSs/hgSs_genome_2f5f_daef20.fa&hgsid=259405959) [details](http://genome.ucsc.edu/cgi-bin/hgc?o=4350&g=htcUserAli&i=../trash/hgSs/hgSs_genome_2f5f_daef20.pslx+..%2Ftrash%2FhgSs%2FhgSs_genome_2f5f_daef20.fa+YourSeq&c=chrM&l=4350&r=4582&db=hg19&hgsid=259405959) YourSeq 232 1 232 232 100.0% M + 4351 4582 232

[browser](http://genome.ucsc.edu/cgi-bin/hgTracks?position=chr1:564900-565131&db=hg19&ss=../trash/hgSs/hgSs_genome_2f5f_daef20.pslx+../trash/hgSs/hgSs_genome_2f5f_daef20.fa&hgsid=259405959) [details](http://genome.ucsc.edu/cgi-bin/hgc?o=564899&g=htcUserAli&i=../trash/hgSs/hgSs_genome_2f5f_daef20.pslx+..%2Ftrash%2FhgSs%2FhgSs_genome_2f5f_daef20.fa+YourSeq&c=chr1&l=564899&r=565131&db=hg19&hgsid=259405959) YourSeq 230 1 232 232 99.6% 1 + 564900 565131 232

[browser](http://genome.ucsc.edu/cgi-bin/hgTracks?position=chr17:22025105-22025336&db=hg19&ss=../trash/hgSs/hgSs_genome_2f5f_daef20.pslx+../trash/hgSs/hgSs_genome_2f5f_daef20.fa&hgsid=259405959) [details](http://genome.ucsc.edu/cgi-bin/hgc?o=22025104&g=htcUserAli&i=../trash/hgSs/hgSs_genome_2f5f_daef20.pslx+..%2Ftrash%2FhgSs%2FhgSs_genome_2f5f_daef20.fa+YourSeq&c=chr17&l=22025104&r=22025336&db=hg19&hgsid=259405959) YourSeq 168 1 232 232 86.3% 17 + 22025105 22025336 232

[browser](http://genome.ucsc.edu/cgi-bin/hgTracks?position=chrX:55206485-55206712&db=hg19&ss=../trash/hgSs/hgSs_genome_2f5f_daef20.pslx+../trash/hgSs/hgSs_genome_2f5f_daef20.fa&hgsid=259405959) [details](http://genome.ucsc.edu/cgi-bin/hgc?o=55206484&g=htcUserAli&i=../trash/hgSs/hgSs_genome_2f5f_daef20.pslx+..%2Ftrash%2FhgSs%2FhgSs_genome_2f5f_daef20.fa+YourSeq&c=chrX&l=55206484&r=55206712&db=hg19&hgsid=259405959) YourSeq 148 5 232 232 82.5% X - 55206485 55206712 228

[browser](http://genome.ucsc.edu/cgi-bin/hgTracks?position=chr2:140977828-140978055&db=hg19&ss=../trash/hgSs/hgSs_genome_2f5f_daef20.pslx+../trash/hgSs/hgSs_genome_2f5f_daef20.fa&hgsid=259405959) [details](http://genome.ucsc.edu/cgi-bin/hgc?o=140977827&g=htcUserAli&i=../trash/hgSs/hgSs_genome_2f5f_daef20.pslx+..%2Ftrash%2FhgSs%2FhgSs_genome_2f5f_daef20.fa+YourSeq&c=chr2&l=140977827&r=140978055&db=hg19&hgsid=259405959) YourSeq 147 1 230 232 80.8% 2 - 140977828 140978055 228

[browser](http://genome.ucsc.edu/cgi-bin/hgTracks?position=chr17:19506612-19506828&db=hg19&ss=../trash/hgSs/hgSs_genome_2f5f_daef20.pslx+../trash/hgSs/hgSs_genome_2f5f_daef20.fa&hgsid=259405959) [details](http://genome.ucsc.edu/cgi-bin/hgc?o=19506611&g=htcUserAli&i=../trash/hgSs/hgSs_genome_2f5f_daef20.pslx+..%2Ftrash%2FhgSs%2FhgSs_genome_2f5f_daef20.fa+YourSeq&c=chr17&l=19506611&r=19506828&db=hg19&hgsid=259405959) YourSeq 139 5 232 232 87.0% 17 + 19506612 19506828 217

[browser](http://genome.ucsc.edu/cgi-bin/hgTracks?position=chr7:63571824-63572040&db=hg19&ss=../trash/hgSs/hgSs_genome_2f5f_daef20.pslx+../trash/hgSs/hgSs_genome_2f5f_daef20.fa&hgsid=259405959) [details](http://genome.ucsc.edu/cgi-bin/hgc?o=63571823&g=htcUserAli&i=../trash/hgSs/hgSs_genome_2f5f_daef20.pslx+..%2Ftrash%2FhgSs%2FhgSs_genome_2f5f_daef20.fa+YourSeq&c=chr7&l=63571823&r=63572040&db=hg19&hgsid=259405959) YourSeq 134 9 227 232 87.7% 7 - 63571824 63572040 217

[browser](http://genome.ucsc.edu/cgi-bin/hgTracks?position=chr11:103277860-103278082&db=hg19&ss=../trash/hgSs/hgSs_genome_2f5f_daef20.pslx+../trash/hgSs/hgSs_genome_2f5f_daef20.fa&hgsid=259405959) [details](http://genome.ucsc.edu/cgi-bin/hgc?o=103277859&g=htcUserAli&i=../trash/hgSs/hgSs_genome_2f5f_daef20.pslx+..%2Ftrash%2FhgSs%2FhgSs_genome_2f5f_daef20.fa+YourSeq&c=chr11&l=103277859&r=103278082&db=hg19&hgsid=259405959) YourSeq 129 8 230 232 79.0% 11 - 103277860 103278082 223

[browser](http://genome.ucsc.edu/cgi-bin/hgTracks?position=chr16:3419632-3419863&db=hg19&ss=../trash/hgSs/hgSs_genome_2f5f_daef20.pslx+../trash/hgSs/hgSs_genome_2f5f_daef20.fa&hgsid=259405959) [details](http://genome.ucsc.edu/cgi-bin/hgc?o=3419631&g=htcUserAli&i=../trash/hgSs/hgSs_genome_2f5f_daef20.pslx+..%2Ftrash%2FhgSs%2FhgSs_genome_2f5f_daef20.fa+YourSeq&c=chr16&l=3419631&r=3419863&db=hg19&hgsid=259405959) YourSeq 126 1 232 232 77.2% 16 - 3419632 3419863 232

[browser](http://genome.ucsc.edu/cgi-bin/hgTracks?position=chr8:32872671-32872894&db=hg19&ss=../trash/hgSs/hgSs_genome_2f5f_daef20.pslx+../trash/hgSs/hgSs_genome_2f5f_daef20.fa&hgsid=259405959) [details](http://genome.ucsc.edu/cgi-bin/hgc?o=32872670&g=htcUserAli&i=../trash/hgSs/hgSs_genome_2f5f_daef20.pslx+..%2Ftrash%2FhgSs%2FhgSs_genome_2f5f_daef20.fa+YourSeq&c=chr8&l=32872670&r=32872894&db=hg19&hgsid=259405959) YourSeq 125 5 232 232 81.5% 8 + 32872671 32872894 224

[browser](http://genome.ucsc.edu/cgi-bin/hgTracks?position=chr2:132143059-132143183&db=hg19&ss=../trash/hgSs/hgSs_genome_2f5f_daef20.pslx+../trash/hgSs/hgSs_genome_2f5f_daef20.fa&hgsid=259405959) [details](http://genome.ucsc.edu/cgi-bin/hgc?o=132143058&g=htcUserAli&i=../trash/hgSs/hgSs_genome_2f5f_daef20.pslx+..%2Ftrash%2FhgSs%2FhgSs_genome_2f5f_daef20.fa+YourSeq&c=chr2&l=132143058&r=132143183&db=hg19&hgsid=259405959) YourSeq 107 1 125 232 92.8% 2 - 132143059 132143183 125

[browser](http://genome.ucsc.edu/cgi-bin/hgTracks?position=chr7:57254001-57254125&db=hg19&ss=../trash/hgSs/hgSs_genome_2f5f_daef20.pslx+../trash/hgSs/hgSs_genome_2f5f_daef20.fa&hgsid=259405959) [details](http://genome.ucsc.edu/cgi-bin/hgc?o=57254000&g=htcUserAli&i=../trash/hgSs/hgSs_genome_2f5f_daef20.pslx+..%2Ftrash%2FhgSs%2FhgSs_genome_2f5f_daef20.fa+YourSeq&c=chr7&l=57254000&r=57254125&db=hg19&hgsid=259405959) YourSeq 107 1 125 232 92.8% 7 + 57254001 57254125 125

[browser](http://genome.ucsc.edu/cgi-bin/hgTracks?position=chr2:131029932-131030056&db=hg19&ss=../trash/hgSs/hgSs_genome_2f5f_daef20.pslx+../trash/hgSs/hgSs_genome_2f5f_daef20.fa&hgsid=259405959) [details](http://genome.ucsc.edu/cgi-bin/hgc?o=131029931&g=htcUserAli&i=../trash/hgSs/hgSs_genome_2f5f_daef20.pslx+..%2Ftrash%2FhgSs%2FhgSs_genome_2f5f_daef20.fa+YourSeq&c=chr2&l=131029931&r=131030056&db=hg19&hgsid=259405959) YourSeq 105 1 125 232 92.0% 2 + 131029932 131030056 125

[browser](http://genome.ucsc.edu/cgi-bin/hgTracks?position=chr1:238105774-238105908&db=hg19&ss=../trash/hgSs/hgSs_genome_2f5f_daef20.pslx+../trash/hgSs/hgSs_genome_2f5f_daef20.fa&hgsid=259405959) [details](http://genome.ucsc.edu/cgi-bin/hgc?o=238105773&g=htcUserAli&i=../trash/hgSs/hgSs_genome_2f5f_daef20.pslx+..%2Ftrash%2FhgSs%2FhgSs_genome_2f5f_daef20.fa+YourSeq&c=chr1&l=238105773&r=238105908&db=hg19&hgsid=259405959) YourSeq 104 5 139 232 88.9% 1 - 238105774 238105908 135

[browser](http://genome.ucsc.edu/cgi-bin/hgTracks?position=chr4:156383810-156383934&db=hg19&ss=../trash/hgSs/hgSs_genome_2f5f_daef20.pslx+../trash/hgSs/hgSs_genome_2f5f_daef20.fa&hgsid=259405959) [details](http://genome.ucsc.edu/cgi-bin/hgc?o=156383809&g=htcUserAli&i=../trash/hgSs/hgSs_genome_2f5f_daef20.pslx+..%2Ftrash%2FhgSs%2FhgSs_genome_2f5f_daef20.fa+YourSeq&c=chr4&l=156383809&r=156383934&db=hg19&hgsid=259405959) YourSeq 101 5 129 232 90.4% 4 - 156383810 156383934 125

[browser](http://genome.ucsc.edu/cgi-bin/hgTracks?position=chr22:24349588-24349718&db=hg19&ss=../trash/hgSs/hgSs_genome_2f5f_daef20.pslx+../trash/hgSs/hgSs_genome_2f5f_daef20.fa&hgsid=259405959) [details](http://genome.ucsc.edu/cgi-bin/hgc?o=24349587&g=htcUserAli&i=../trash/hgSs/hgSs_genome_2f5f_daef20.pslx+..%2Ftrash%2FhgSs%2FhgSs_genome_2f5f_daef20.fa+YourSeq&c=chr22&l=24349587&r=24349718&db=hg19&hgsid=259405959) YourSeq 98 8 128 232 93.1% 22 + 24349588 24349718 131

[browser](http://genome.ucsc.edu/cgi-bin/hgTracks?position=chr3:106620903-106621131&db=hg19&ss=../trash/hgSs/hgSs_genome_2f5f_daef20.pslx+../trash/hgSs/hgSs_genome_2f5f_daef20.fa&hgsid=259405959) [details](http://genome.ucsc.edu/cgi-bin/hgc?o=106620902&g=htcUserAli&i=../trash/hgSs/hgSs_genome_2f5f_daef20.pslx+..%2Ftrash%2FhgSs%2FhgSs_genome_2f5f_daef20.fa+YourSeq&c=chr3&l=106620902&r=106621131&db=hg19&hgsid=259405959) YourSeq 96 5 232 232 89.4% 3 + 106620903 106621131 229

[browser](http://genome.ucsc.edu/cgi-bin/hgTracks?position=chrY:8240167-8240289&db=hg19&ss=../trash/hgSs/hgSs_genome_2f5f_daef20.pslx+../trash/hgSs/hgSs_genome_2f5f_daef20.fa&hgsid=259405959) [details](http://genome.ucsc.edu/cgi-bin/hgc?o=8240166&g=htcUserAli&i=../trash/hgSs/hgSs_genome_2f5f_daef20.pslx+..%2Ftrash%2FhgSs%2FhgSs_genome_2f5f_daef20.fa+YourSeq&c=chrY&l=8240166&r=8240289&db=hg19&hgsid=259405959) YourSeq 95 6 128 232 88.7% Y + 8240167 8240289 123

[browser](http://genome.ucsc.edu/cgi-bin/hgTracks?position=chr10:71355025-71355149&db=hg19&ss=../trash/hgSs/hgSs_genome_2f5f_daef20.pslx+../trash/hgSs/hgSs_genome_2f5f_daef20.fa&hgsid=259405959) [details](http://genome.ucsc.edu/cgi-bin/hgc?o=71355024&g=htcUserAli&i=../trash/hgSs/hgSs_genome_2f5f_daef20.pslx+..%2Ftrash%2FhgSs%2FhgSs_genome_2f5f_daef20.fa+YourSeq&c=chr10&l=71355024&r=71355149&db=hg19&hgsid=259405959) YourSeq 90 5 128 232 87.5% 10 - 71355025 71355149 125

[browser](http://genome.ucsc.edu/cgi-bin/hgTracks?position=chr7:141503180-141503361&db=hg19&ss=../trash/hgSs/hgSs_genome_2f5f_daef20.pslx+../trash/hgSs/hgSs_genome_2f5f_daef20.fa&hgsid=259405959) [details](http://genome.ucsc.edu/cgi-bin/hgc?o=141503179&g=htcUserAli&i=../trash/hgSs/hgSs_genome_2f5f_daef20.pslx+..%2Ftrash%2FhgSs%2FhgSs_genome_2f5f_daef20.fa+YourSeq&c=chr7&l=141503179&r=141503361&db=hg19&hgsid=259405959) YourSeq 78 47 232 232 83.4% 7 - 141503180 141503361 182

[browser](http://genome.ucsc.edu/cgi-bin/hgTracks?position=chr9:81357642-81357734&db=hg19&ss=../trash/hgSs/hgSs_genome_2f5f_daef20.pslx+../trash/hgSs/hgSs_genome_2f5f_daef20.fa&hgsid=259405959) [details](http://genome.ucsc.edu/cgi-bin/hgc?o=81357641&g=htcUserAli&i=../trash/hgSs/hgSs_genome_2f5f_daef20.pslx+..%2Ftrash%2FhgSs%2FhgSs_genome_2f5f_daef20.fa+YourSeq&c=chr9&l=81357641&r=81357734&db=hg19&hgsid=259405959) YourSeq 75 47 139 232 90.4% 9 - 81357642 81357734 93

[browser](http://genome.ucsc.edu/cgi-bin/hgTracks?position=chr6:95156836-95156916&db=hg19&ss=../trash/hgSs/hgSs_genome_2f5f_daef20.pslx+../trash/hgSs/hgSs_genome_2f5f_daef20.fa&hgsid=259405959) [details](http://genome.ucsc.edu/cgi-bin/hgc?o=95156835&g=htcUserAli&i=../trash/hgSs/hgSs_genome_2f5f_daef20.pslx+..%2Ftrash%2FhgSs%2FhgSs_genome_2f5f_daef20.fa+YourSeq&c=chr6&l=95156835&r=95156916&db=hg19&hgsid=259405959) YourSeq 71 45 125 232 93.9% 6 - 95156836 95156916 81

[browser](http://genome.ucsc.edu/cgi-bin/hgTracks?position=chr12:127067855-127068923&db=hg19&ss=../trash/hgSs/hgSs_genome_2f5f_daef20.pslx+../trash/hgSs/hgSs_genome_2f5f_daef20.fa&hgsid=259405959) [details](http://genome.ucsc.edu/cgi-bin/hgc?o=127067854&g=htcUserAli&i=../trash/hgSs/hgSs_genome_2f5f_daef20.pslx+..%2Ftrash%2FhgSs%2FhgSs_genome_2f5f_daef20.fa+YourSeq&c=chr12&l=127067854&r=127068923&db=hg19&hgsid=259405959) YourSeq 71 47 128 232 94.0% 12 - 127067855 127068923 1069

[browser](http://genome.ucsc.edu/cgi-bin/hgTracks?position=chr9:5095151-5095234&db=hg19&ss=../trash/hgSs/hgSs_genome_2f5f_daef20.pslx+../trash/hgSs/hgSs_genome_2f5f_daef20.fa&hgsid=259405959) [details](http://genome.ucsc.edu/cgi-bin/hgc?o=5095150&g=htcUserAli&i=../trash/hgSs/hgSs_genome_2f5f_daef20.pslx+..%2Ftrash%2FhgSs%2FhgSs_genome_2f5f_daef20.fa+YourSeq&c=chr9&l=5095150&r=5095234&db=hg19&hgsid=259405959) YourSeq 70 46 128 232 94.9% 9 + 5095151 5095234 84

[browser](http://genome.ucsc.edu/cgi-bin/hgTracks?position=chr3:68708207-68708282&db=hg19&ss=../trash/hgSs/hgSs_genome_2f5f_daef20.pslx+../trash/hgSs/hgSs_genome_2f5f_daef20.fa&hgsid=259405959) [details](http://genome.ucsc.edu/cgi-bin/hgc?o=68708206&g=htcUserAli&i=../trash/hgSs/hgSs_genome_2f5f_daef20.pslx+..%2Ftrash%2FhgSs%2FhgSs_genome_2f5f_daef20.fa+YourSeq&c=chr3&l=68708206&r=68708282&db=hg19&hgsid=259405959) YourSeq 70 5 80 232 96.1% 3 + 68708207 68708282 76

[browser](http://genome.ucsc.edu/cgi-bin/hgTracks?position=chr10:20036686-20036759&db=hg19&ss=../trash/hgSs/hgSs_genome_2f5f_daef20.pslx+../trash/hgSs/hgSs_genome_2f5f_daef20.fa&hgsid=259405959) [details](http://genome.ucsc.edu/cgi-bin/hgc?o=20036685&g=htcUserAli&i=../trash/hgSs/hgSs_genome_2f5f_daef20.pslx+..%2Ftrash%2FhgSs%2FhgSs_genome_2f5f_daef20.fa+YourSeq&c=chr10&l=20036685&r=20036759&db=hg19&hgsid=259405959) YourSeq 64 56 129 232 93.3% 10 + 20036686 20036759 74

[browser](http://genome.ucsc.edu/cgi-bin/hgTracks?position=chr8:47739510-47739588&db=hg19&ss=../trash/hgSs/hgSs_genome_2f5f_daef20.pslx+../trash/hgSs/hgSs_genome_2f5f_daef20.fa&hgsid=259405959) [details](http://genome.ucsc.edu/cgi-bin/hgc?o=47739509&g=htcUserAli&i=../trash/hgSs/hgSs_genome_2f5f_daef20.pslx+..%2Ftrash%2FhgSs%2FhgSs_genome_2f5f_daef20.fa+YourSeq&c=chr8&l=47739509&r=47739588&db=hg19&hgsid=259405959) YourSeq 63 50 128 232 89.9% 8 - 47739510 47739588 79

[browser](http://genome.ucsc.edu/cgi-bin/hgTracks?position=chr12:50211216-50211285&db=hg19&ss=../trash/hgSs/hgSs_genome_2f5f_daef20.pslx+../trash/hgSs/hgSs_genome_2f5f_daef20.fa&hgsid=259405959) [details](http://genome.ucsc.edu/cgi-bin/hgc?o=50211215&g=htcUserAli&i=../trash/hgSs/hgSs_genome_2f5f_daef20.pslx+..%2Ftrash%2FhgSs%2FhgSs_genome_2f5f_daef20.fa+YourSeq&c=chr12&l=50211215&r=50211285&db=hg19&hgsid=259405959) YourSeq 56 5 74 232 90.0% 12 + 50211216 50211285 70

[browser](http://genome.ucsc.edu/cgi-bin/hgTracks?position=chr12:42093031-42093227&db=hg19&ss=../trash/hgSs/hgSs_genome_2f5f_daef20.pslx+../trash/hgSs/hgSs_genome_2f5f_daef20.fa&hgsid=259405959) [details](http://genome.ucsc.edu/cgi-bin/hgc?o=42093030&g=htcUserAli&i=../trash/hgSs/hgSs_genome_2f5f_daef20.pslx+..%2Ftrash%2FhgSs%2FhgSs_genome_2f5f_daef20.fa+YourSeq&c=chr12&l=42093030&r=42093227&db=hg19&hgsid=259405959) YourSeq 55 1 76 232 86.9% 12 - 42093031 42093227 197

[browser](http://genome.ucsc.edu/cgi-bin/hgTracks?position=chr13:36639772-36639833&db=hg19&ss=../trash/hgSs/hgSs_genome_2f5f_daef20.pslx+../trash/hgSs/hgSs_genome_2f5f_daef20.fa&hgsid=259405959) [details](http://genome.ucsc.edu/cgi-bin/hgc?o=36639771&g=htcUserAli&i=../trash/hgSs/hgSs_genome_2f5f_daef20.pslx+..%2Ftrash%2FhgSs%2FhgSs_genome_2f5f_daef20.fa+YourSeq&c=chr13&l=36639771&r=36639833&db=hg19&hgsid=259405959) YourSeq 52 5 66 232 92.0% 13 + 36639772 36639833 62

[browser](http://genome.ucsc.edu/cgi-bin/hgTracks?position=chr10:131929406-131929468&db=hg19&ss=../trash/hgSs/hgSs_genome_2f5f_daef20.pslx+../trash/hgSs/hgSs_genome_2f5f_daef20.fa&hgsid=259405959) [details](http://genome.ucsc.edu/cgi-bin/hgc?o=131929405&g=htcUserAli&i=../trash/hgSs/hgSs_genome_2f5f_daef20.pslx+..%2Ftrash%2FhgSs%2FhgSs_genome_2f5f_daef20.fa+YourSeq&c=chr10&l=131929405&r=131929468&db=hg19&hgsid=259405959) YourSeq 49 12 74 232 88.9% 10 + 131929406 131929468 63

[browser](http://genome.ucsc.edu/cgi-bin/hgTracks?position=chr8:104099268-104099319&db=hg19&ss=../trash/hgSs/hgSs_genome_2f5f_daef20.pslx+../trash/hgSs/hgSs_genome_2f5f_daef20.fa&hgsid=259405959) [details](http://genome.ucsc.edu/cgi-bin/hgc?o=104099267&g=htcUserAli&i=../trash/hgSs/hgSs_genome_2f5f_daef20.pslx+..%2Ftrash%2FhgSs%2FhgSs_genome_2f5f_daef20.fa+YourSeq&c=chr8&l=104099267&r=104099319&db=hg19&hgsid=259405959) YourSeq 40 53 103 232 90.2% 8 + 104099268 104099319 52

[browser](http://genome.ucsc.edu/cgi-bin/hgTracks?position=chr12:127067916-127067962&db=hg19&ss=../trash/hgSs/hgSs_genome_2f5f_daef20.pslx+../trash/hgSs/hgSs_genome_2f5f_daef20.fa&hgsid=259405959) [details](http://genome.ucsc.edu/cgi-bin/hgc?o=127067915&g=htcUserAli&i=../trash/hgSs/hgSs_genome_2f5f_daef20.pslx+..%2Ftrash%2FhgSs%2FhgSs_genome_2f5f_daef20.fa+YourSeq&c=chr12&l=127067915&r=127067962&db=hg19&hgsid=259405959) YourSeq 39 68 114 232 91.5% 12 - 127067916 127067962 47

[browser](http://genome.ucsc.edu/cgi-bin/hgTracks?position=chr12:127068057-127068103&db=hg19&ss=../trash/hgSs/hgSs_genome_2f5f_daef20.pslx+../trash/hgSs/hgSs_genome_2f5f_daef20.fa&hgsid=259405959) [details](http://genome.ucsc.edu/cgi-bin/hgc?o=127068056&g=htcUserAli&i=../trash/hgSs/hgSs_genome_2f5f_daef20.pslx+..%2Ftrash%2FhgSs%2FhgSs_genome_2f5f_daef20.fa+YourSeq&c=chr12&l=127068056&r=127068103&db=hg19&hgsid=259405959) YourSeq 39 68 114 232 91.5% 12 - 127068057 127068103 47

[browser](http://genome.ucsc.edu/cgi-bin/hgTracks?position=chr12:127068114-127068150&db=hg19&ss=../trash/hgSs/hgSs_genome_2f5f_daef20.pslx+../trash/hgSs/hgSs_genome_2f5f_daef20.fa&hgsid=259405959) [details](http://genome.ucsc.edu/cgi-bin/hgc?o=127068113&g=htcUserAli&i=../trash/hgSs/hgSs_genome_2f5f_daef20.pslx+..%2Ftrash%2FhgSs%2FhgSs_genome_2f5f_daef20.fa+YourSeq&c=chr12&l=127068113&r=127068150&db=hg19&hgsid=259405959) YourSeq 33 68 104 232 94.6% 12 - 127068114 127068150 37

[browser](http://genome.ucsc.edu/cgi-bin/hgTracks?position=chr12:127068208-127068244&db=hg19&ss=../trash/hgSs/hgSs_genome_2f5f_daef20.pslx+../trash/hgSs/hgSs_genome_2f5f_daef20.fa&hgsid=259405959) [details](http://genome.ucsc.edu/cgi-bin/hgc?o=127068207&g=htcUserAli&i=../trash/hgSs/hgSs_genome_2f5f_daef20.pslx+..%2Ftrash%2FhgSs%2FhgSs_genome_2f5f_daef20.fa+YourSeq&c=chr12&l=127068207&r=127068244&db=hg19&hgsid=259405959) YourSeq 31 68 104 232 91.9% 12 - 127068208 127068244 37

[browser](http://genome.ucsc.edu/cgi-bin/hgTracks?position=chr12:127068302-127068338&db=hg19&ss=../trash/hgSs/hgSs_genome_2f5f_daef20.pslx+../trash/hgSs/hgSs_genome_2f5f_daef20.fa&hgsid=259405959) [details](http://genome.ucsc.edu/cgi-bin/hgc?o=127068301&g=htcUserAli&i=../trash/hgSs/hgSs_genome_2f5f_daef20.pslx+..%2Ftrash%2FhgSs%2FhgSs_genome_2f5f_daef20.fa+YourSeq&c=chr12&l=127068301&r=127068338&db=hg19&hgsid=259405959) YourSeq 31 68 104 232 91.9% 12 - 127068302 127068338 37

[browser](http://genome.ucsc.edu/cgi-bin/hgTracks?position=chr12:127068349-127068385&db=hg19&ss=../trash/hgSs/hgSs_genome_2f5f_daef20.pslx+../trash/hgSs/hgSs_genome_2f5f_daef20.fa&hgsid=259405959) [details](http://genome.ucsc.edu/cgi-bin/hgc?o=127068348&g=htcUserAli&i=../trash/hgSs/hgSs_genome_2f5f_daef20.pslx+..%2Ftrash%2FhgSs%2FhgSs_genome_2f5f_daef20.fa+YourSeq&c=chr12&l=127068348&r=127068385&db=hg19&hgsid=259405959) YourSeq 31 68 104 232 91.9% 12 - 127068349 127068385 37

[browser](http://genome.ucsc.edu/cgi-bin/hgTracks?position=chr12:19948592-19948620&db=hg19&ss=../trash/hgSs/hgSs_genome_2f5f_daef20.pslx+../trash/hgSs/hgSs_genome_2f5f_daef20.fa&hgsid=259405959) [details](http://genome.ucsc.edu/cgi-bin/hgc?o=19948591&g=htcUserAli&i=../trash/hgSs/hgSs_genome_2f5f_daef20.pslx+..%2Ftrash%2FhgSs%2FhgSs_genome_2f5f_daef20.fa+YourSeq&c=chr12&l=19948591&r=19948620&db=hg19&hgsid=259405959) YourSeq 27 40 68 232 96.6% 12 - 19948592 19948620 29

[browser](http://genome.ucsc.edu/cgi-bin/hgTracks?position=chr1:206082680-206082702&db=hg19&ss=../trash/hgSs/hgSs_genome_2f5f_daef20.pslx+../trash/hgSs/hgSs_genome_2f5f_daef20.fa&hgsid=259405959) [details](http://genome.ucsc.edu/cgi-bin/hgc?o=206082679&g=htcUserAli&i=../trash/hgSs/hgSs_genome_2f5f_daef20.pslx+..%2Ftrash%2FhgSs%2FhgSs_genome_2f5f_daef20.fa+YourSeq&c=chr1&l=206082679&r=206082702&db=hg19&hgsid=259405959) YourSeq 22 185 208 232 87.0% 1 + 206082680 206082702 23

[browser](http://genome.ucsc.edu/cgi-bin/hgTracks?position=chr6_ssto_hap7:3972955-3972974&db=hg19&ss=../trash/hgSs/hgSs_genome_2f5f_daef20.pslx+../trash/hgSs/hgSs_genome_2f5f_daef20.fa&hgsid=259405959) [details](http://genome.ucsc.edu/cgi-bin/hgc?o=3972954&g=htcUserAli&i=../trash/hgSs/hgSs_genome_2f5f_daef20.pslx+..%2Ftrash%2FhgSs%2FhgSs_genome_2f5f_daef20.fa+YourSeq&c=chr6_ssto_hap7&l=3972954&r=3972974&db=hg19&hgsid=259405959) YourSeq 20 7 26 232 100.0% 6_ssto_hap7 + 3972955 3972974 20

[browser](http://genome.ucsc.edu/cgi-bin/hgTracks?position=chr6_mann_hap4:3989087-3989106&db=hg19&ss=../trash/hgSs/hgSs_genome_2f5f_daef20.pslx+../trash/hgSs/hgSs_genome_2f5f_daef20.fa&hgsid=259405959) [details](http://genome.ucsc.edu/cgi-bin/hgc?o=3989086&g=htcUserAli&i=../trash/hgSs/hgSs_genome_2f5f_daef20.pslx+..%2Ftrash%2FhgSs%2FhgSs_genome_2f5f_daef20.fa+YourSeq&c=chr6_mann_hap4&l=3989086&r=3989106&db=hg19&hgsid=259405959) YourSeq 20 7 26 232 100.0% 6_mann_hap4 + 3989087 3989106 20

[browser](http://genome.ucsc.edu/cgi-bin/hgTracks?position=chr6_dbb_hap3:3813464-3813483&db=hg19&ss=../trash/hgSs/hgSs_genome_2f5f_daef20.pslx+../trash/hgSs/hgSs_genome_2f5f_daef20.fa&hgsid=259405959) [details](http://genome.ucsc.edu/cgi-bin/hgc?o=3813463&g=htcUserAli&i=../trash/hgSs/hgSs_genome_2f5f_daef20.pslx+..%2Ftrash%2FhgSs%2FhgSs_genome_2f5f_daef20.fa+YourSeq&c=chr6_dbb_hap3&l=3813463&r=3813483&db=hg19&hgsid=259405959) YourSeq 20 7 26 232 100.0% 6_dbb_hap3 + 3813464 3813483 20

[browser](http://genome.ucsc.edu/cgi-bin/hgTracks?position=chr10:46073554-46073573&db=hg19&ss=../trash/hgSs/hgSs_genome_2f5f_daef20.pslx+../trash/hgSs/hgSs_genome_2f5f_daef20.fa&hgsid=259405959) [details](http://genome.ucsc.edu/cgi-bin/hgc?o=46073553&g=htcUserAli&i=../trash/hgSs/hgSs_genome_2f5f_daef20.pslx+..%2Ftrash%2FhgSs%2FhgSs_genome_2f5f_daef20.fa+YourSeq&c=chr10&l=46073553&r=46073573&db=hg19&hgsid=259405959) YourSeq 20 207 226 232 100.0% 10 + 46073554 46073573 20

**Reads BLAT results**

7: chrM:4417-4517(+)

AATAAGCTATCGGGCCCATACCCCGAAAATGTTGGTTATA**T**CCTTCCCGTACTAATTAATCCCCTGGCCCAACCCGTCATCTACTCTACCATCTTTGCAGG

## chr1:564966-565066

AATAAGCTATCGGGCCCATACCCCGAAAATGTTGGTTATATCCTTCCCGTACTAATTAATCCCCTGGCCCAACCCGTCATCTACTCTACCATCTTTGCAGG

## chr17:22025171-22025256

AATAAGCTATCaGGCCCATACCCaGAAAATGTTGGTTATATCCTTCCCGTACTAATcAATCCaCTGGCCCAACCtGTCATCTACTC

chrM:4421-4521(+)

AGCTATCGGGCCCATACCCCGAAAATGTTGGTTATA**T**CCTTCCCGTACTAATTAATCCCCTGGCCCAACCCGTCATCTACTCTACCATCTTTGCAGGCACA

## chr1:564970-565070

AGCTATCGGGCCCATACCCCGAAAATGTTGGTTATATCCTTCCCGTACTAATTAATCCCCTGGCCCAACCCGTCATCTACTCTACCATCTTTGCAGGCACA

chrM:4372-4472(+)

TCTCCGTGCCACCTATCACACCCCATCCTAAAGTAAGGTCAGCTAAATAAGCTATCGGGCCCATACCCCGAAAATGTTGGTTATA**T**CCTTCCCGTACTAAT

## chr1:564921-565021

TCTCCGTGCCACCTATCACACCCCATCCTAAAGTAAGGTCAGCTAAATAAGCTATCGGGCCCATACCCCGAAAATGTTGGTTATATCCTTCCCGTACTAAT

chrM:4370-44470(+)

ATTCTCCGTGCCACCTATCACACCCCATCCTAAAGTAAGGTCAGCTAAATAAGCTATCGGGCCCATACCCCGAAAATGTTGGTTATA**T**CCTTCCCGTACTA

## chr1:564919-565019

ATTCTCCGTGCCACCTATCACACCCCATCCTAAAGTAAGGTCAGCTAAATAAGCTATCGGGCCCATACCCCGAAAATGTTGGTTATATCCTTCCCGTACTA

8: no variant detected with HiSeq at this position

**Primer BLAST results**

>[NT_004350.19](http://www.ncbi.nlm.nih.gov/entrez/viewer.fcgi?db=nucleotide&id=224514624) Homo sapiens chromosome 1 genomic contig, GRCh37.p5 Primary Assembly

product length = 232

Forward primer 1 CCATCCCTGAGAATCCAAAA 20

Template 43532 .................... 43551

Reverse primer 1 GCATGTTTATTTCTAGGCCTACTC 24

Template 43763 ........................ 43740

>[NT_005612.16](http://www.ncbi.nlm.nih.gov/entrez/viewer.fcgi?db=nucleotide&id=224514994) Homo sapiens chromosome 3 genomic contig, GRCh37.p5 Primary Assembly

product length = 233

Forward primer 1 CCATCCCTGAGAATCCAAAA 20

Template 13116045 .T.C................ 13116064

Reverse primer 1 GCATGTTTATTTCTAGGCCTACTC 24

Template 13116277 .T...................T.. 13116254

>[NT_007933.15](http://www.ncbi.nlm.nih.gov/entrez/viewer.fcgi?db=nucleotide&id=224514692) Homo sapiens chromosome 7 genomic contig, GRCh37.p5 Primary Assembly

product length = 308

Forward primer 1 CCATCCCTGAGAATCCAAAA 20

Template 14486605 ..T..T........T....G 14486624

Forward primer 1 CCATCCCTGAGAATCCAAAA 20

Template 14486912 ..T..T........T....G 14486893

**Numts aligned to the variant on the UCSC genome browser**


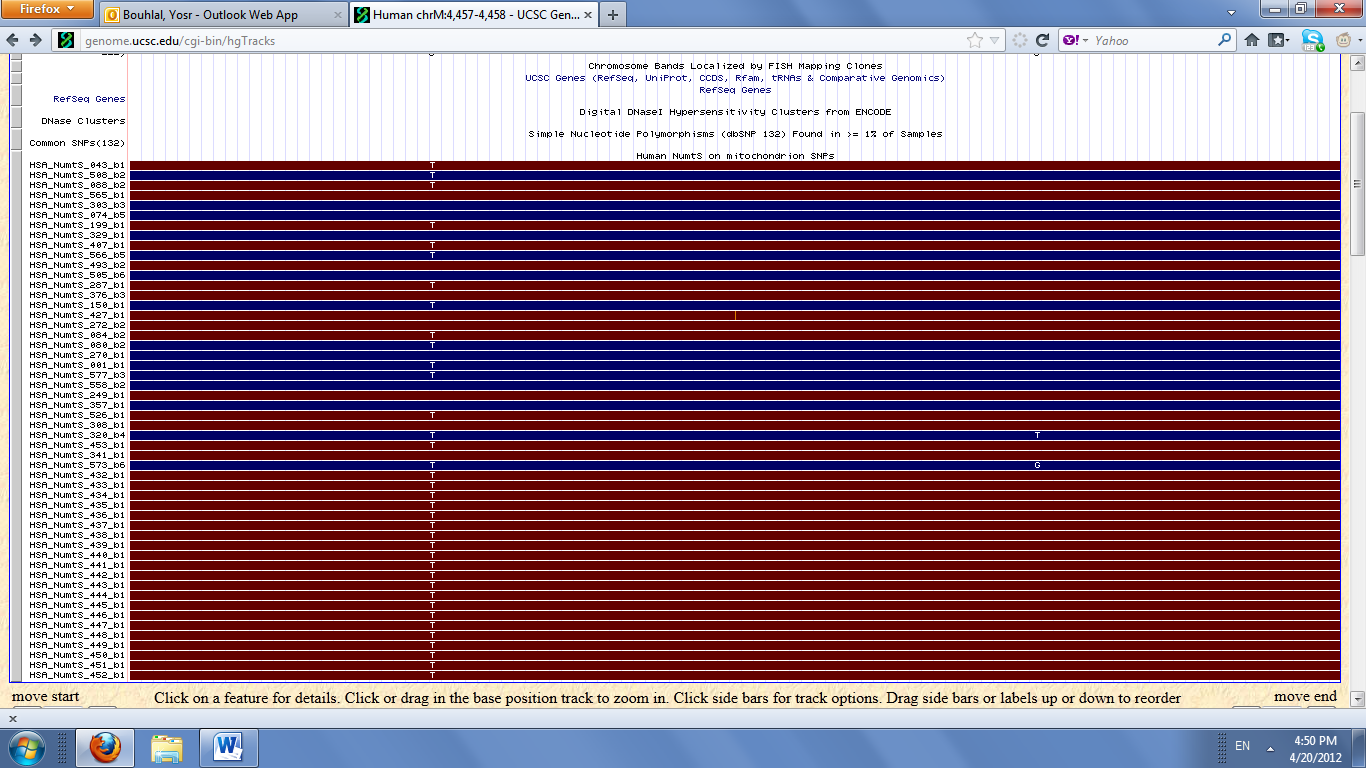


Numts 52: 1T, 1G

**Variant3: m.4998 A>T/G**

**Template Fragment**

**CGTAAGCCTTCTCCTCACTCTC**TCAATCTTATCCATCATAGCAGGCAGTTGAGGTGGATTAAACCAAACCCAGCTACGC**A**AAATCTTAGCATACTCCTCAATTACCCACATAGGATGAATAATAGCAGTTCTACCGTACAACCCTAACATAACCATTCTTAATTTAACTATTTATATTATCCTAACTACTACCGCATTCCTACTACTCAA**CTTAAACTCCAGCACCACGA**

**Template BLAT results**

ACTIONS QUERY SCORE START END QSIZE IDENTITY CHRO STRAND START END SPAN

---------------------------------------------------------------------------------------------------

[browser](http://genome.ucsc.edu/cgi-bin/hgTracks?position=chrM:4920-5149&db=hg19&ss=../trash/hgSs/hgSs_genome_3572_766d50.pslx+../trash/hgSs/hgSs_genome_3572_766d50.fa&hgsid=256713665) [details](http://genome.ucsc.edu/cgi-bin/hgc?o=4919&g=htcUserAli&i=../trash/hgSs/hgSs_genome_3572_766d50.pslx+..%2Ftrash%2FhgSs%2FhgSs_genome_3572_766d50.fa+YourSeq&c=chrM&l=4919&r=5149&db=hg19&hgsid=256713665) YourSeq 230 1 230 230 100.0% M + 4920 5149 230

[browser](http://genome.ucsc.edu/cgi-bin/hgTracks?position=chr1:565469-565698&db=hg19&ss=../trash/hgSs/hgSs_genome_3572_766d50.pslx+../trash/hgSs/hgSs_genome_3572_766d50.fa&hgsid=256713665) [details](http://genome.ucsc.edu/cgi-bin/hgc?o=565468&g=htcUserAli&i=../trash/hgSs/hgSs_genome_3572_766d50.pslx+..%2Ftrash%2FhgSs%2FhgSs_genome_3572_766d50.fa+YourSeq&c=chr1&l=565468&r=565698&db=hg19&hgsid=256713665) YourSeq 220 1 230 230 97.9% 1 + 565469 565698 230

[browser](http://genome.ucsc.edu/cgi-bin/hgTracks?position=chr2:156121031-156121182&db=hg19&ss=../trash/hgSs/hgSs_genome_3572_766d50.pslx+../trash/hgSs/hgSs_genome_3572_766d50.fa&hgsid=256713665) [details](http://genome.ucsc.edu/cgi-bin/hgc?o=156121030&g=htcUserAli&i=../trash/hgSs/hgSs_genome_3572_766d50.pslx+..%2Ftrash%2FhgSs%2FhgSs_genome_3572_766d50.fa+YourSeq&c=chr2&l=156121030&r=156121182&db=hg19&hgsid=256713665) YourSeq 96 74 225 230 81.6% 2 - 156121031 156121182 152

[browser](http://genome.ucsc.edu/cgi-bin/hgTracks?position=chr17:22025676-22025811&db=hg19&ss=../trash/hgSs/hgSs_genome_3572_766d50.pslx+../trash/hgSs/hgSs_genome_3572_766d50.fa&hgsid=256713665) [details](http://genome.ucsc.edu/cgi-bin/hgc?o=22025675&g=htcUserAli&i=../trash/hgSs/hgSs_genome_3572_766d50.pslx+..%2Ftrash%2FhgSs%2FhgSs_genome_3572_766d50.fa+YourSeq&c=chr17&l=22025675&r=22025811&db=hg19&hgsid=256713665) YourSeq 94 9 144 230 84.6% 17 + 22025676 22025811 136

[browser](http://genome.ucsc.edu/cgi-bin/hgTracks?position=chr1:81546496-81546563&db=hg19&ss=../trash/hgSs/hgSs_genome_3572_766d50.pslx+../trash/hgSs/hgSs_genome_3572_766d50.fa&hgsid=256713665) [details](http://genome.ucsc.edu/cgi-bin/hgc?o=81546495&g=htcUserAli&i=../trash/hgSs/hgSs_genome_3572_766d50.pslx+..%2Ftrash%2FhgSs%2FhgSs_genome_3572_766d50.fa+YourSeq&c=chr1&l=81546495&r=81546563&db=hg19&hgsid=256713665) YourSeq 54 62 129 230 89.8% 1 + 81546496 81546563 68

[browser](http://genome.ucsc.edu/cgi-bin/hgTracks?position=chr17:19507799-19507897&db=hg19&ss=../trash/hgSs/hgSs_genome_3572_766d50.pslx+../trash/hgSs/hgSs_genome_3572_766d50.fa&hgsid=256713665) [details](http://genome.ucsc.edu/cgi-bin/hgc?o=19507798&g=htcUserAli&i=../trash/hgSs/hgSs_genome_3572_766d50.pslx+..%2Ftrash%2FhgSs%2FhgSs_genome_3572_766d50.fa+YourSeq&c=chr17&l=19507798&r=19507897&db=hg19&hgsid=256713665) YourSeq 51 24 129 230 81.0% 17 + 19507799 19507897 99

[browser](http://genome.ucsc.edu/cgi-bin/hgTracks?position=chr13:56545850-56545890&db=hg19&ss=../trash/hgSs/hgSs_genome_3572_766d50.pslx+../trash/hgSs/hgSs_genome_3572_766d50.fa&hgsid=256713665) [details](http://genome.ucsc.edu/cgi-bin/hgc?o=56545849&g=htcUserAli&i=../trash/hgSs/hgSs_genome_3572_766d50.pslx+..%2Ftrash%2FhgSs%2FhgSs_genome_3572_766d50.fa+YourSeq&c=chr13&l=56545849&r=56545890&db=hg19&hgsid=256713665) YourSeq 35 190 230 230 92.7% 13 - 56545850 56545890 41

[browser](http://genome.ucsc.edu/cgi-bin/hgTracks?position=chr21:43826954-43826987&db=hg19&ss=../trash/hgSs/hgSs_genome_3572_766d50.pslx+../trash/hgSs/hgSs_genome_3572_766d50.fa&hgsid=256713665) [details](http://genome.ucsc.edu/cgi-bin/hgc?o=43826953&g=htcUserAli&i=../trash/hgSs/hgSs_genome_3572_766d50.pslx+..%2Ftrash%2FhgSs%2FhgSs_genome_3572_766d50.fa+YourSeq&c=chr21&l=43826953&r=43826987&db=hg19&hgsid=256713665) YourSeq 30 21 54 230 94.2% 21 - 43826954 43826987 34

[browser](http://genome.ucsc.edu/cgi-bin/hgTracks?position=chr5:132391361-132391660&db=hg19&ss=../trash/hgSs/hgSs_genome_3572_766d50.pslx+../trash/hgSs/hgSs_genome_3572_766d50.fa&hgsid=256713665) [details](http://genome.ucsc.edu/cgi-bin/hgc?o=132391360&g=htcUserAli&i=../trash/hgSs/hgSs_genome_3572_766d50.pslx+..%2Ftrash%2FhgSs%2FhgSs_genome_3572_766d50.fa+YourSeq&c=chr5&l=132391360&r=132391660&db=hg19&hgsid=256713665) YourSeq 24 53 77 230 100.0% 5 + 132391361 132391660 300

[browser](http://genome.ucsc.edu/cgi-bin/hgTracks?position=chr6_ssto_hap7:762545-762565&db=hg19&ss=../trash/hgSs/hgSs_genome_3572_766d50.pslx+../trash/hgSs/hgSs_genome_3572_766d50.fa&hgsid=256713665) [details](http://genome.ucsc.edu/cgi-bin/hgc?o=762544&g=htcUserAli&i=../trash/hgSs/hgSs_genome_3572_766d50.pslx+..%2Ftrash%2FhgSs%2FhgSs_genome_3572_766d50.fa+YourSeq&c=chr6_ssto_hap7&l=762544&r=762565&db=hg19&hgsid=256713665) YourSeq 21 159 179 230 100.0% 6_ssto_hap7 - 762545 762565 21

[browser](http://genome.ucsc.edu/cgi-bin/hgTracks?position=chr6_qbl_hap6:725301-725321&db=hg19&ss=../trash/hgSs/hgSs_genome_3572_766d50.pslx+../trash/hgSs/hgSs_genome_3572_766d50.fa&hgsid=256713665) [details](http://genome.ucsc.edu/cgi-bin/hgc?o=725300&g=htcUserAli&i=../trash/hgSs/hgSs_genome_3572_766d50.pslx+..%2Ftrash%2FhgSs%2FhgSs_genome_3572_766d50.fa+YourSeq&c=chr6_qbl_hap6&l=725300&r=725321&db=hg19&hgsid=256713665) YourSeq 21 159 179 230 100.0% 6_qbl_hap6 - 725301 725321 21

[browser](http://genome.ucsc.edu/cgi-bin/hgTracks?position=chr6_mcf_hap5:725191-725211&db=hg19&ss=../trash/hgSs/hgSs_genome_3572_766d50.pslx+../trash/hgSs/hgSs_genome_3572_766d50.fa&hgsid=256713665) [details](http://genome.ucsc.edu/cgi-bin/hgc?o=725190&g=htcUserAli&i=../trash/hgSs/hgSs_genome_3572_766d50.pslx+..%2Ftrash%2FhgSs%2FhgSs_genome_3572_766d50.fa+YourSeq&c=chr6_mcf_hap5&l=725190&r=725211&db=hg19&hgsid=256713665) YourSeq 21 159 179 230 100.0% 6_mcf_hap5 - 725191 725211 21

[browser](http://genome.ucsc.edu/cgi-bin/hgTracks?position=chr6_mann_hap4:724984-725004&db=hg19&ss=../trash/hgSs/hgSs_genome_3572_766d50.pslx+../trash/hgSs/hgSs_genome_3572_766d50.fa&hgsid=256713665) [details](http://genome.ucsc.edu/cgi-bin/hgc?o=724983&g=htcUserAli&i=../trash/hgSs/hgSs_genome_3572_766d50.pslx+..%2Ftrash%2FhgSs%2FhgSs_genome_3572_766d50.fa+YourSeq&c=chr6_mann_hap4&l=724983&r=725004&db=hg19&hgsid=256713665) YourSeq 21 159 179 230 100.0% 6_mann_hap4 - 724984 725004 21

[browser](http://genome.ucsc.edu/cgi-bin/hgTracks?position=chr6_dbb_hap3:725342-725362&db=hg19&ss=../trash/hgSs/hgSs_genome_3572_766d50.pslx+../trash/hgSs/hgSs_genome_3572_766d50.fa&hgsid=256713665) [details](http://genome.ucsc.edu/cgi-bin/hgc?o=725341&g=htcUserAli&i=../trash/hgSs/hgSs_genome_3572_766d50.pslx+..%2Ftrash%2FhgSs%2FhgSs_genome_3572_766d50.fa+YourSeq&c=chr6_dbb_hap3&l=725341&r=725362&db=hg19&hgsid=256713665) YourSeq 21 159 179 230 100.0% 6_dbb_hap3 - 725342 725362 21

[browser](http://genome.ucsc.edu/cgi-bin/hgTracks?position=chr6_cox_hap2:940792-940812&db=hg19&ss=../trash/hgSs/hgSs_genome_3572_766d50.pslx+../trash/hgSs/hgSs_genome_3572_766d50.fa&hgsid=256713665) [details](http://genome.ucsc.edu/cgi-bin/hgc?o=940791&g=htcUserAli&i=../trash/hgSs/hgSs_genome_3572_766d50.pslx+..%2Ftrash%2FhgSs%2FhgSs_genome_3572_766d50.fa+YourSeq&c=chr6_cox_hap2&l=940791&r=940812&db=hg19&hgsid=256713665) YourSeq 21 159 179 230 100.0% 6_cox_hap2 - 940792 940812 21

[browser](http://genome.ucsc.edu/cgi-bin/hgTracks?position=chr6_apd_hap1:725494-725514&db=hg19&ss=../trash/hgSs/hgSs_genome_3572_766d50.pslx+../trash/hgSs/hgSs_genome_3572_766d50.fa&hgsid=256713665) [details](http://genome.ucsc.edu/cgi-bin/hgc?o=725493&g=htcUserAli&i=../trash/hgSs/hgSs_genome_3572_766d50.pslx+..%2Ftrash%2FhgSs%2FhgSs_genome_3572_766d50.fa+YourSeq&c=chr6_apd_hap1&l=725493&r=725514&db=hg19&hgsid=256713665) YourSeq 21 159 179 230 100.0% 6_apd_hap1 - 725494 725514 21

[browser](http://genome.ucsc.edu/cgi-bin/hgTracks?position=chr6:29422012-29422032&db=hg19&ss=../trash/hgSs/hgSs_genome_3572_766d50.pslx+../trash/hgSs/hgSs_genome_3572_766d50.fa&hgsid=256713665) [details](http://genome.ucsc.edu/cgi-bin/hgc?o=29422011&g=htcUserAli&i=../trash/hgSs/hgSs_genome_3572_766d50.pslx+..%2Ftrash%2FhgSs%2FhgSs_genome_3572_766d50.fa+YourSeq&c=chr6&l=29422011&r=29422032&db=hg19&hgsid=256713665) YourSeq 21 159 179 230 100.0% 6 - 29422012 29422032 21

[browser](http://genome.ucsc.edu/cgi-bin/hgTracks?position=chr1:158842085-158842104&db=hg19&ss=../trash/hgSs/hgSs_genome_3572_766d50.pslx+../trash/hgSs/hgSs_genome_3572_766d50.fa&hgsid=256713665) [details](http://genome.ucsc.edu/cgi-bin/hgc?o=158842084&g=htcUserAli&i=../trash/hgSs/hgSs_genome_3572_766d50.pslx+..%2Ftrash%2FhgSs%2FhgSs_genome_3572_766d50.fa+YourSeq&c=chr1&l=158842084&r=158842104&db=hg19&hgsid=256713665) YourSeq 20 159 178 230 100.0% 1 - 158842085 158842104 20

**Reads BLAT results**

7: chrM:4936-5036 (-)

CCTCTCTCAATCTTATCCATCATAGCAGGCAGTTGAGGTGGATTAAACCAAACCCAGCTACGC**G**AAATCTTAGCATACTCCTCAATTACCCACATAGGATG

**chr1:565486-565585**

CTCTtTCAATCTTATCCATCATgGCAGGCAGTTGAGGTGGATTAAACCAAACCCAaCTACGC**a**AAATCTTAGCATACTCCTCAATTACCCACATAGGATG

8: chrM:4955-5055 (+)

TCATAGCAGGCAGTTGAGGTGGATTAAACCAAACCCAGCTACGC**T**AAATCTTAGCATACTCCTCAATTACCCACATAGGATGAATAATAGCAGTTCTACCG

**chr1:565509-565604**

GCAGGCAGTTGAGGTGGATTAAACCAAACCCAaCTACGC**a**AAATCTTAGCATACTCCTCAATTACCCACATAGGATGAATAAcAGCAGTTCTACCG

**chr17:22025720-22025796**

GGTGGAcTgAACCAAACCCAaCTAtGt**a**AAATCcTAGCATACTCCTCAATcACCCAtATAGGcTGAATAATAGCAGT

**Primers BLAST results**

>[NT_008470.19](http://www.ncbi.nlm.nih.gov/entrez/viewer.fcgi?db=nucleotide&id=224514751) Homo sapiens chromosome 9 genomic contig, GRCh37.p5 Primary Assembly

product length = 237

Forward primer 1 TAGCCCCCTTTCACTTCTGA 20

Template 12345194 .................... 12345175

Reverse primer 1 TGTGGGTAATTGAGGAGTATGC 22

Template 12344958 ....A..G...........A.. 12344979

>[NT_023678.16](http://www.ncbi.nlm.nih.gov/entrez/viewer.fcgi?db=nucleotide&id=224514816) Homo sapiens chromosome 8 genomic contig, GRCh37.p5 Primary Assembly

product length = 236

Forward primer 1 TAGCCCCCTTTCACTTCTGA 20

Template 900309 .......T............ 900290

Reverse primer 1 TGTGGGTAATTGAGGAGTATGC 22

Template 900074 ....A..............G.. 900095

>[NT_016354.19](http://www.ncbi.nlm.nih.gov/entrez/viewer.fcgi?db=nucleotide&id=224514665) Homo sapiens chromosome 4 genomic contig, GRCh37.p5 Primary Assembly

product length = 230

Forward primer 1 TAGCCCCCTTTCACTTCTGA 20

Template 80931219 .................... 80931200

Reverse primer 1 TGTGGGTAATTGAGGAGTATGC 22

Template 80930990 ....A..G.C.........G.. 80931011

>[NT_011630.14](http://www.ncbi.nlm.nih.gov/entrez/viewer.fcgi?db=nucleotide&id=37546193) Homo sapiens chromosome X genomic contig, GRCh37.p5 Primary Assembly

product length = 234

Forward primer 1 TAGCCCCCTTTCACTTCTGA 20

Template 2760355 .............A...... 2760336

Reverse primer 1 TGTGGGTAATTGAGGAGTATGC 22

Template 2760122 ....A..G...........G.. 2760143

>[NT_007914.15](http://www.ncbi.nlm.nih.gov/entrez/viewer.fcgi?db=nucleotide&id=224514812) Homo sapiens chromosome 7 genomic contig, GRCh37.p5 Primary Assembly

product length = 237

Forward primer 1 TAGCCCCCTTTCACTTCTGA 20

Template 2098595 ......G............. 2098576

Reverse primer 1 TGTGGGTAATTGAGGAGTATGC 22

Template 2098359 G...A..............G.. 2098380

>[NT_006316.16](http://www.ncbi.nlm.nih.gov/entrez/viewer.fcgi?db=nucleotide&id=224514620) Homo sapiens chromosome 4 genomic contig, GRCh37.p5 Primary Assembly

product length = 229

Forward primer 1 TAGCCCCCTTTCACTTCTGA 20

Template 5689722 ..........C......... 5689703

Reverse primer 1 TGTGGGTAATTGAGGAGTATGC 22

Template 5689494 ....A..G....G......... 5689515

>[NT_007933.15](http://www.ncbi.nlm.nih.gov/entrez/viewer.fcgi?db=nucleotide&id=224514692) Homo sapiens chromosome 7 genomic contig, GRCh37.p5 Primary Assembly

product length = 236

Forward primer 1 TAGCCCCCTTTCACTTCTGA 20

Template 1604461 .......T............ 1604442

Reverse primer 1 TGTGGGTAATTGAGGAGTATGC 22

Template 1604226 .A..AA.............G.. 1604247

>[NT_022135.16](http://www.ncbi.nlm.nih.gov/entrez/viewer.fcgi?db=nucleotide&id=224514673) Homo sapiens chromosome 2 genomic contig, GRCh37.p5 Primary Assembly

product length = 238

Forward primer 1 TAGCCCCCTTTCACTTCTGA 20

Template 30726284 ..........C........G 30726265

Reverse primer 1 TGTGGGTAATTGAGGAGTATGC 22

Template 30726047 ....A...G..........G.. 30726068

>[NT_167186.1](http://www.ncbi.nlm.nih.gov/entrez/viewer.fcgi?db=nucleotide&id=224514622) Homo sapiens chromosome 1 genomic contig, GRCh37.p5 Primary Assembly

product length = 237

Forward primer 1 TAGCCCCCTTTCACTTCTGA 20

Template 31623249 .....A.............. 31623230

Reverse primer 1 TGTGGGTAATTGAGGAGTATGC 22

Template 31623013 ....A...C.C........G.. 31623034

**Numts aligned to the variant on the UCSC genome browser**


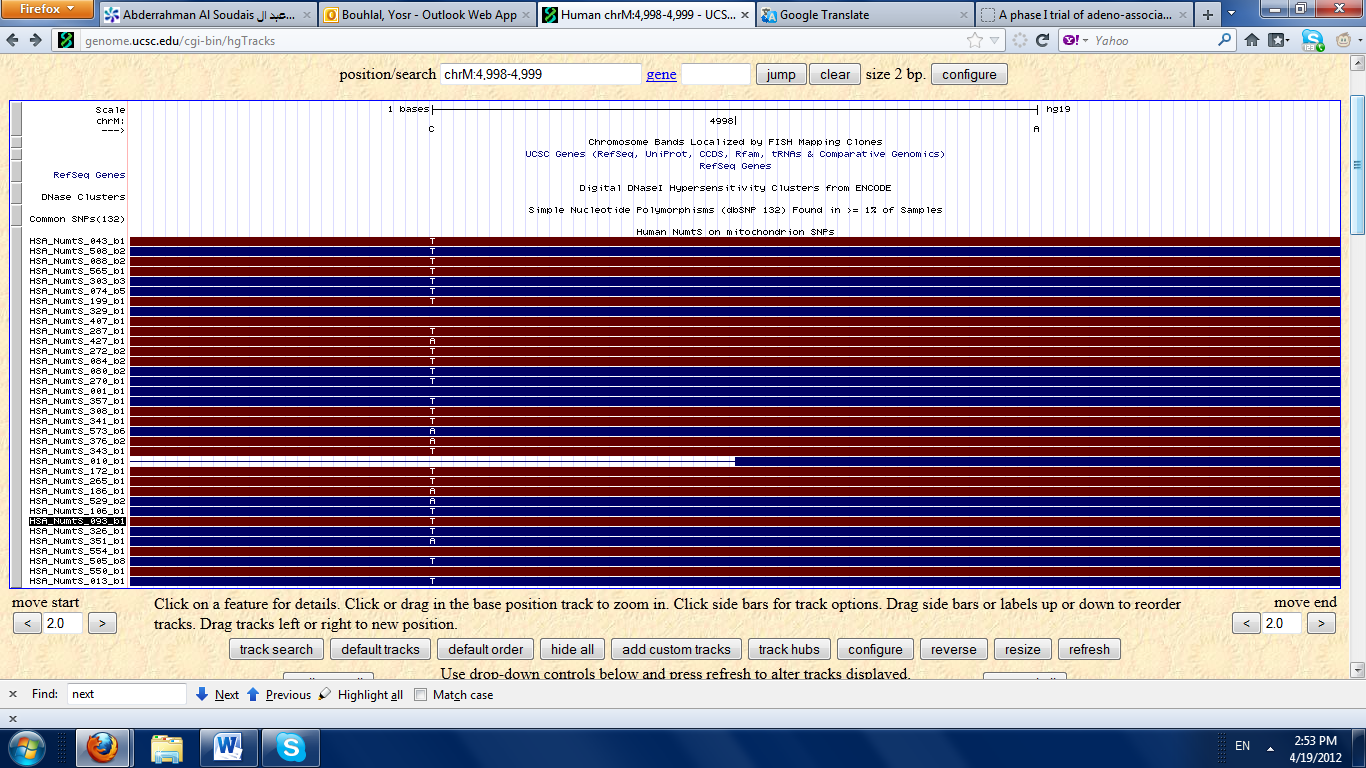


Numts 35 A

**Variant4: m.5014C>G/A**

**Template Fragment**

**CGTAAGCCTTCTCCTCACTCTC**TCAATCTTATCCATCATAGCAGGCAGTTGAGGTGGATTAAACCAAACCCAGCTACGCAAAATCTTAGCATACT**C**CTCAATTACCCACATAGGATGAATAATAGCAGTTCTACCGTACAACCCTAACATAACCATTCTTAATTTAACTATTTATATTATCCTAACTACTACCGCATTCCTACTACTCAA**CTTAAACTCCAGCACCACGA**

**Template BLAT results**

ACTIONS QUERY SCORE START END QSIZE IDENTITY CHRO STRAND START END SPAN

---------------------------------------------------------------------------------------------------

[browser](http://genome.ucsc.edu/cgi-bin/hgTracks?position=chrM:4920-5149&db=hg19&ss=../trash/hgSs/hgSs_genome_3318_c49b90.pslx+../trash/hgSs/hgSs_genome_3318_c49b90.fa&hgsid=258719997) [details](http://genome.ucsc.edu/cgi-bin/hgc?o=4919&g=htcUserAli&i=../trash/hgSs/hgSs_genome_3318_c49b90.pslx+..%2Ftrash%2FhgSs%2FhgSs_genome_3318_c49b90.fa+YourSeq&c=chrM&l=4919&r=5149&db=hg19&hgsid=258719997) YourSeq 230 1 230 230 100.0% M + 4920 5149 230

[browser](http://genome.ucsc.edu/cgi-bin/hgTracks?position=chr1:565469-565698&db=hg19&ss=../trash/hgSs/hgSs_genome_3318_c49b90.pslx+../trash/hgSs/hgSs_genome_3318_c49b90.fa&hgsid=258719997) [details](http://genome.ucsc.edu/cgi-bin/hgc?o=565468&g=htcUserAli&i=../trash/hgSs/hgSs_genome_3318_c49b90.pslx+..%2Ftrash%2FhgSs%2FhgSs_genome_3318_c49b90.fa+YourSeq&c=chr1&l=565468&r=565698&db=hg19&hgsid=258719997) YourSeq 220 1 230 230 97.9% 1 + 565469 565698 230

[browser](http://genome.ucsc.edu/cgi-bin/hgTracks?position=chr2:156121031-156121182&db=hg19&ss=../trash/hgSs/hgSs_genome_3318_c49b90.pslx+../trash/hgSs/hgSs_genome_3318_c49b90.fa&hgsid=258719997) [details](http://genome.ucsc.edu/cgi-bin/hgc?o=156121030&g=htcUserAli&i=../trash/hgSs/hgSs_genome_3318_c49b90.pslx+..%2Ftrash%2FhgSs%2FhgSs_genome_3318_c49b90.fa+YourSeq&c=chr2&l=156121030&r=156121182&db=hg19&hgsid=258719997) YourSeq 96 74 225 230 81.6% 2 - 156121031 156121182 152

[browser](http://genome.ucsc.edu/cgi-bin/hgTracks?position=chr17:22025676-22025811&db=hg19&ss=../trash/hgSs/hgSs_genome_3318_c49b90.pslx+../trash/hgSs/hgSs_genome_3318_c49b90.fa&hgsid=258719997) [details](http://genome.ucsc.edu/cgi-bin/hgc?o=22025675&g=htcUserAli&i=../trash/hgSs/hgSs_genome_3318_c49b90.pslx+..%2Ftrash%2FhgSs%2FhgSs_genome_3318_c49b90.fa+YourSeq&c=chr17&l=22025675&r=22025811&db=hg19&hgsid=258719997) YourSeq 94 9 144 230 84.6% 17 + 22025676 22025811 136

[browser](http://genome.ucsc.edu/cgi-bin/hgTracks?position=chr1:81546496-81546563&db=hg19&ss=../trash/hgSs/hgSs_genome_3318_c49b90.pslx+../trash/hgSs/hgSs_genome_3318_c49b90.fa&hgsid=258719997) [details](http://genome.ucsc.edu/cgi-bin/hgc?o=81546495&g=htcUserAli&i=../trash/hgSs/hgSs_genome_3318_c49b90.pslx+..%2Ftrash%2FhgSs%2FhgSs_genome_3318_c49b90.fa+YourSeq&c=chr1&l=81546495&r=81546563&db=hg19&hgsid=258719997) YourSeq 54 62 129 230 89.8% 1 + 81546496 81546563 68

[browser](http://genome.ucsc.edu/cgi-bin/hgTracks?position=chr17:19507799-19507897&db=hg19&ss=../trash/hgSs/hgSs_genome_3318_c49b90.pslx+../trash/hgSs/hgSs_genome_3318_c49b90.fa&hgsid=258719997) [details](http://genome.ucsc.edu/cgi-bin/hgc?o=19507798&g=htcUserAli&i=../trash/hgSs/hgSs_genome_3318_c49b90.pslx+..%2Ftrash%2FhgSs%2FhgSs_genome_3318_c49b90.fa+YourSeq&c=chr17&l=19507798&r=19507897&db=hg19&hgsid=258719997) YourSeq 51 24 129 230 81.0% 17 + 19507799 19507897 99

[browser](http://genome.ucsc.edu/cgi-bin/hgTracks?position=chr13:56545850-56545890&db=hg19&ss=../trash/hgSs/hgSs_genome_3318_c49b90.pslx+../trash/hgSs/hgSs_genome_3318_c49b90.fa&hgsid=258719997) [details](http://genome.ucsc.edu/cgi-bin/hgc?o=56545849&g=htcUserAli&i=../trash/hgSs/hgSs_genome_3318_c49b90.pslx+..%2Ftrash%2FhgSs%2FhgSs_genome_3318_c49b90.fa+YourSeq&c=chr13&l=56545849&r=56545890&db=hg19&hgsid=258719997) YourSeq 35 190 230 230 92.7% 13 - 56545850 56545890 41

[browser](http://genome.ucsc.edu/cgi-bin/hgTracks?position=chr21:43826954-43826987&db=hg19&ss=../trash/hgSs/hgSs_genome_3318_c49b90.pslx+../trash/hgSs/hgSs_genome_3318_c49b90.fa&hgsid=258719997) [details](http://genome.ucsc.edu/cgi-bin/hgc?o=43826953&g=htcUserAli&i=../trash/hgSs/hgSs_genome_3318_c49b90.pslx+..%2Ftrash%2FhgSs%2FhgSs_genome_3318_c49b90.fa+YourSeq&c=chr21&l=43826953&r=43826987&db=hg19&hgsid=258719997) YourSeq 30 21 54 230 94.2% 21 - 43826954 43826987 34

[browser](http://genome.ucsc.edu/cgi-bin/hgTracks?position=chr5:132391361-132391660&db=hg19&ss=../trash/hgSs/hgSs_genome_3318_c49b90.pslx+../trash/hgSs/hgSs_genome_3318_c49b90.fa&hgsid=258719997) [details](http://genome.ucsc.edu/cgi-bin/hgc?o=132391360&g=htcUserAli&i=../trash/hgSs/hgSs_genome_3318_c49b90.pslx+..%2Ftrash%2FhgSs%2FhgSs_genome_3318_c49b90.fa+YourSeq&c=chr5&l=132391360&r=132391660&db=hg19&hgsid=258719997) YourSeq 24 53 77 230 100.0% 5 + 132391361 132391660 300

[browser](http://genome.ucsc.edu/cgi-bin/hgTracks?position=chr6_ssto_hap7:762545-762565&db=hg19&ss=../trash/hgSs/hgSs_genome_3318_c49b90.pslx+../trash/hgSs/hgSs_genome_3318_c49b90.fa&hgsid=258719997) [details](http://genome.ucsc.edu/cgi-bin/hgc?o=762544&g=htcUserAli&i=../trash/hgSs/hgSs_genome_3318_c49b90.pslx+..%2Ftrash%2FhgSs%2FhgSs_genome_3318_c49b90.fa+YourSeq&c=chr6_ssto_hap7&l=762544&r=762565&db=hg19&hgsid=258719997) YourSeq 21 159 179 230 100.0% 6_ssto_hap7 - 762545 762565 21

[browser](http://genome.ucsc.edu/cgi-bin/hgTracks?position=chr6_qbl_hap6:725301-725321&db=hg19&ss=../trash/hgSs/hgSs_genome_3318_c49b90.pslx+../trash/hgSs/hgSs_genome_3318_c49b90.fa&hgsid=258719997) [details](http://genome.ucsc.edu/cgi-bin/hgc?o=725300&g=htcUserAli&i=../trash/hgSs/hgSs_genome_3318_c49b90.pslx+..%2Ftrash%2FhgSs%2FhgSs_genome_3318_c49b90.fa+YourSeq&c=chr6_qbl_hap6&l=725300&r=725321&db=hg19&hgsid=258719997) YourSeq 21 159 179 230 100.0% 6_qbl_hap6 - 725301 725321 21

[browser](http://genome.ucsc.edu/cgi-bin/hgTracks?position=chr6_mcf_hap5:725191-725211&db=hg19&ss=../trash/hgSs/hgSs_genome_3318_c49b90.pslx+../trash/hgSs/hgSs_genome_3318_c49b90.fa&hgsid=258719997) [details](http://genome.ucsc.edu/cgi-bin/hgc?o=725190&g=htcUserAli&i=../trash/hgSs/hgSs_genome_3318_c49b90.pslx+..%2Ftrash%2FhgSs%2FhgSs_genome_3318_c49b90.fa+YourSeq&c=chr6_mcf_hap5&l=725190&r=725211&db=hg19&hgsid=258719997) YourSeq 21 159 179 230 100.0% 6_mcf_hap5 - 725191 725211 21

[browser](http://genome.ucsc.edu/cgi-bin/hgTracks?position=chr6_mann_hap4:724984-725004&db=hg19&ss=../trash/hgSs/hgSs_genome_3318_c49b90.pslx+../trash/hgSs/hgSs_genome_3318_c49b90.fa&hgsid=258719997) [details](http://genome.ucsc.edu/cgi-bin/hgc?o=724983&g=htcUserAli&i=../trash/hgSs/hgSs_genome_3318_c49b90.pslx+..%2Ftrash%2FhgSs%2FhgSs_genome_3318_c49b90.fa+YourSeq&c=chr6_mann_hap4&l=724983&r=725004&db=hg19&hgsid=258719997) YourSeq 21 159 179 230 100.0% 6_mann_hap4 - 724984 725004 21

[browser](http://genome.ucsc.edu/cgi-bin/hgTracks?position=chr6_dbb_hap3:725342-725362&db=hg19&ss=../trash/hgSs/hgSs_genome_3318_c49b90.pslx+../trash/hgSs/hgSs_genome_3318_c49b90.fa&hgsid=258719997) [details](http://genome.ucsc.edu/cgi-bin/hgc?o=725341&g=htcUserAli&i=../trash/hgSs/hgSs_genome_3318_c49b90.pslx+..%2Ftrash%2FhgSs%2FhgSs_genome_3318_c49b90.fa+YourSeq&c=chr6_dbb_hap3&l=725341&r=725362&db=hg19&hgsid=258719997) YourSeq 21 159 179 230 100.0% 6_dbb_hap3 - 725342 725362 21

[browser](http://genome.ucsc.edu/cgi-bin/hgTracks?position=chr6:29422012-29422032&db=hg19&ss=../trash/hgSs/hgSs_genome_3318_c49b90.pslx+../trash/hgSs/hgSs_genome_3318_c49b90.fa&hgsid=258719997) [details](http://genome.ucsc.edu/cgi-bin/hgc?o=29422011&g=htcUserAli&i=../trash/hgSs/hgSs_genome_3318_c49b90.pslx+..%2Ftrash%2FhgSs%2FhgSs_genome_3318_c49b90.fa+YourSeq&c=chr6&l=29422011&r=29422032&db=hg19&hgsid=258719997) YourSeq 21 159 179 230 100.0% 6 - 29422012 29422032 21

[browser](http://genome.ucsc.edu/cgi-bin/hgTracks?position=chr1:158842085-158842104&db=hg19&ss=../trash/hgSs/hgSs_genome_3318_c49b90.pslx+../trash/hgSs/hgSs_genome_3318_c49b90.fa&hgsid=258719997) [details](http://genome.ucsc.edu/cgi-bin/hgc?o=158842084&g=htcUserAli&i=../trash/hgSs/hgSs_genome_3318_c49b90.pslx+..%2Ftrash%2FhgSs%2FhgSs_genome_3318_c49b90.fa+YourSeq&c=chr1&l=158842084&r=158842104&db=hg19&hgsid=258719997) YourSeq 20 159 178 230 100.0% 1 - 158842085 158842104 20

[browser](http://genome.ucsc.edu/cgi-bin/hgTracks?position=chr1:87486196-87486217&db=hg19&ss=../trash/hgSs/hgSs_genome_3318_c49b90.pslx+../trash/hgSs/hgSs_genome_3318_c49b90.fa&hgsid=258719997) [details](http://genome.ucsc.edu/cgi-bin/hgc?o=87486195&g=htcUserAli&i=../trash/hgSs/hgSs_genome_3318_c49b90.pslx+..%2Ftrash%2FhgSs%2FhgSs_genome_3318_c49b90.fa+YourSeq&c=chr1&l=87486195&r=87486217&db=hg19&hgsid=258719997) YourSeq 20 152 173 230 95.5% 1 + 87486196 87486217 22

**Reads BLAT results**

7: chrM:4990-5090 (+)

CAGCTACGCAAAATCTTAGCATACT**A**CTCAATTATCCACATAGGATGAATAATAGCAGTTCTACCGTACAACCCTAACATAACCATTCTTAATTTAACTAT

## chr1:565539-565639

CAaCTACGCAAAATCTTAGCATACTcCTCAATTAcCCACATAGGATGAATAAcAGCAGTTCTACCGTACAACCCTAACATAACCATTCTTAATTTAACTAT

8: chrM:4949-5049 (-)

TATCCATCATAGCAGGCAGTTGAGGTGGATTAAACCAAACCCAGCTACGCAAAATCTTAGCATACT**G**CTCAATTACCCACATAGGATGAATAATAGCAGTT

## chr1:565498-565598

TATCCATCATgGCAGGCAGTTGAGGTGGATTAAACCAAACCCAaCTACGCAAAATCTTAGCATACTcCTCAATTACCCACATAGGATGAATAAcAGCAGTT

**Numts aligned to the variant on the UCSC genome browser**


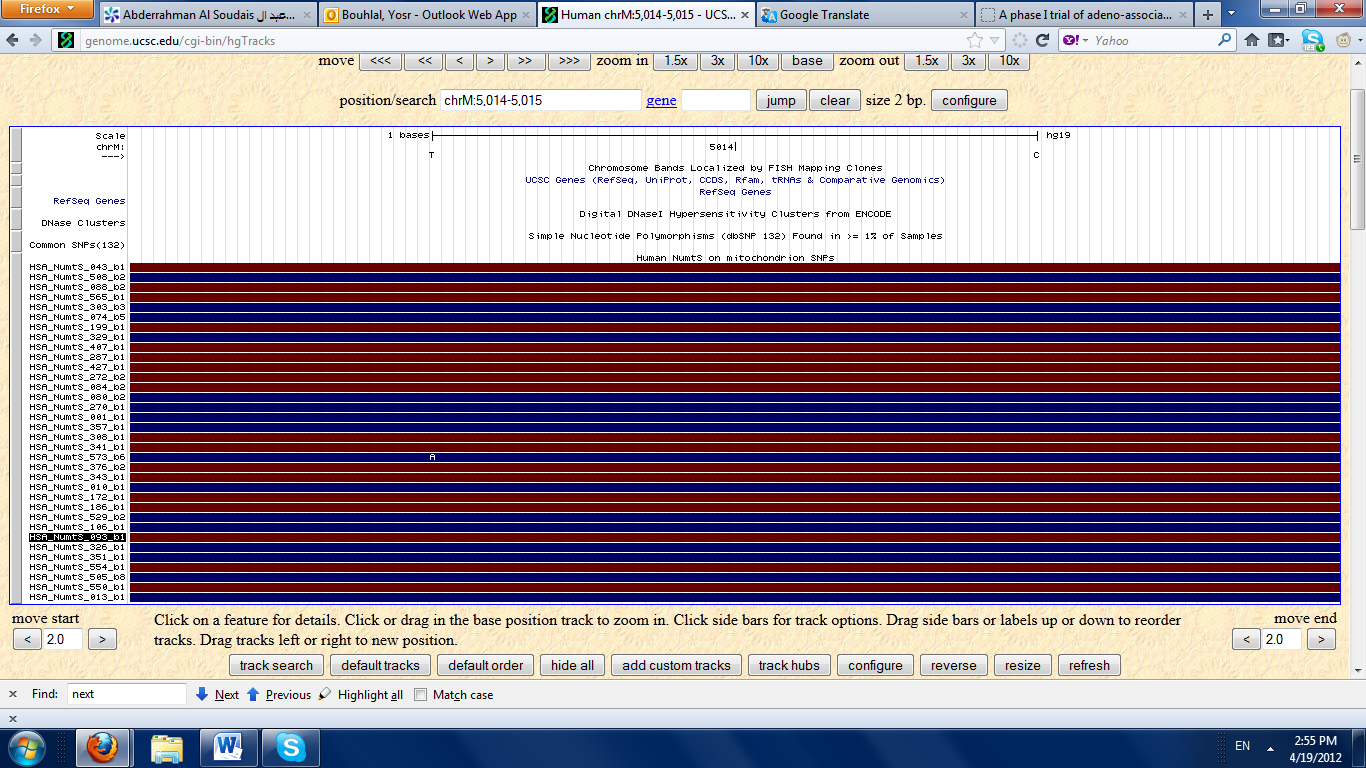


Numts 34 all ref base C

**Variant5: m.5906 G>A/T**

**Template Fragment**

**CACCTCGGAGCTGGTAAAAA**GAGGCCTAACCCCTGTCTTTAGATTTACAGTCCAATGCTTCACTCAGCCATTTTACCTCACCCCCACTGAT**G**TTCGCCGACCGTTGACTATTCTCTACAAACCACAAAGACATTGGAACACTATACCTATTATTCGGC**GCATGAGCTGGAGTCCTAGG**

**Template BLAT results**

ACTIONS QUERY SCORE START END QSIZE IDENTITY CHRO STRAND START END SPAN

---------------------------------------------------------------------------------------------------

[browser](http://genome.ucsc.edu/cgi-bin/hgTracks?position=chrM:5816-5993&db=hg19&ss=../trash/hgSs/hgSs_genome_1239_d9e300.pslx+../trash/hgSs/hgSs_genome_1239_d9e300.fa&hgsid=259405959) [details](http://genome.ucsc.edu/cgi-bin/hgc?o=5815&g=htcUserAli&i=../trash/hgSs/hgSs_genome_1239_d9e300.pslx+..%2Ftrash%2FhgSs%2FhgSs_genome_1239_d9e300.fa+YourSeq&c=chrM&l=5815&r=5993&db=hg19&hgsid=259405959) YourSeq 178 1 178 178 100.0% M + 5816 5993 178

[browser](http://genome.ucsc.edu/cgi-bin/hgTracks?position=chr1:566365-566542&db=hg19&ss=../trash/hgSs/hgSs_genome_1239_d9e300.pslx+../trash/hgSs/hgSs_genome_1239_d9e300.fa&hgsid=259405959) [details](http://genome.ucsc.edu/cgi-bin/hgc?o=566364&g=htcUserAli&i=../trash/hgSs/hgSs_genome_1239_d9e300.pslx+..%2Ftrash%2FhgSs%2FhgSs_genome_1239_d9e300.fa+YourSeq&c=chr1&l=566364&r=566542&db=hg19&hgsid=259405959) YourSeq 174 1 178 178 98.9% 1 + 566365 566542 178

[browser](http://genome.ucsc.edu/cgi-bin/hgTracks?position=chr14:32953918-32954094&db=hg19&ss=../trash/hgSs/hgSs_genome_1239_d9e300.pslx+../trash/hgSs/hgSs_genome_1239_d9e300.fa&hgsid=259405959) [details](http://genome.ucsc.edu/cgi-bin/hgc?o=32953917&g=htcUserAli&i=../trash/hgSs/hgSs_genome_1239_d9e300.pslx+..%2Ftrash%2FhgSs%2FhgSs_genome_1239_d9e300.fa+YourSeq&c=chr14&l=32953917&r=32954094&db=hg19&hgsid=259405959) YourSeq 156 1 178 178 93.7% 14 - 32953918 32954094 177

[browser](http://genome.ucsc.edu/cgi-bin/hgTracks?position=chr17:19508678-19508833&db=hg19&ss=../trash/hgSs/hgSs_genome_1239_d9e300.pslx+../trash/hgSs/hgSs_genome_1239_d9e300.fa&hgsid=259405959) [details](http://genome.ucsc.edu/cgi-bin/hgc?o=19508677&g=htcUserAli&i=../trash/hgSs/hgSs_genome_1239_d9e300.pslx+..%2Ftrash%2FhgSs%2FhgSs_genome_1239_d9e300.fa+YourSeq&c=chr17&l=19508677&r=19508833&db=hg19&hgsid=259405959) YourSeq 102 10 165 178 84.4% 17 + 19508678 19508833 156

[browser](http://genome.ucsc.edu/cgi-bin/hgTracks?position=chr4:156382331-156382478&db=hg19&ss=../trash/hgSs/hgSs_genome_1239_d9e300.pslx+../trash/hgSs/hgSs_genome_1239_d9e300.fa&hgsid=259405959) [details](http://genome.ucsc.edu/cgi-bin/hgc?o=156382330&g=htcUserAli&i=../trash/hgSs/hgSs_genome_1239_d9e300.pslx+..%2Ftrash%2FhgSs%2FhgSs_genome_1239_d9e300.fa+YourSeq&c=chr4&l=156382330&r=156382478&db=hg19&hgsid=259405959) YourSeq 99 1 148 178 84.6% 4 - 156382331 156382478 148

[browser](http://genome.ucsc.edu/cgi-bin/hgTracks?position=chr2:203484539-203484701&db=hg19&ss=../trash/hgSs/hgSs_genome_1239_d9e300.pslx+../trash/hgSs/hgSs_genome_1239_d9e300.fa&hgsid=259405959) [details](http://genome.ucsc.edu/cgi-bin/hgc?o=203484538&g=htcUserAli&i=../trash/hgSs/hgSs_genome_1239_d9e300.pslx+..%2Ftrash%2FhgSs%2FhgSs_genome_1239_d9e300.fa+YourSeq&c=chr2&l=203484538&r=203484701&db=hg19&hgsid=259405959) YourSeq 95 10 171 178 90.6% 2 - 203484539 203484701 163

[browser](http://genome.ucsc.edu/cgi-bin/hgTracks?position=chr9:83179488-83179635&db=hg19&ss=../trash/hgSs/hgSs_genome_1239_d9e300.pslx+../trash/hgSs/hgSs_genome_1239_d9e300.fa&hgsid=259405959) [details](http://genome.ucsc.edu/cgi-bin/hgc?o=83179487&g=htcUserAli&i=../trash/hgSs/hgSs_genome_1239_d9e300.pslx+..%2Ftrash%2FhgSs%2FhgSs_genome_1239_d9e300.fa+YourSeq&c=chr9&l=83179487&r=83179635&db=hg19&hgsid=259405959) YourSeq 94 1 148 178 85.0% 9 - 83179488 83179635 148

[browser](http://genome.ucsc.edu/cgi-bin/hgTracks?position=chr9:5096585-5096741&db=hg19&ss=../trash/hgSs/hgSs_genome_1239_d9e300.pslx+../trash/hgSs/hgSs_genome_1239_d9e300.fa&hgsid=259405959) [details](http://genome.ucsc.edu/cgi-bin/hgc?o=5096584&g=htcUserAli&i=../trash/hgSs/hgSs_genome_1239_d9e300.pslx+..%2Ftrash%2FhgSs%2FhgSs_genome_1239_d9e300.fa+YourSeq&c=chr9&l=5096584&r=5096741&db=hg19&hgsid=259405959) YourSeq 85 10 165 178 91.2% 9 + 5096585 5096741 157

[browser](http://genome.ucsc.edu/cgi-bin/hgTracks?position=chr2:140974739-140974897&db=hg19&ss=../trash/hgSs/hgSs_genome_1239_d9e300.pslx+../trash/hgSs/hgSs_genome_1239_d9e300.fa&hgsid=259405959) [details](http://genome.ucsc.edu/cgi-bin/hgc?o=140974738&g=htcUserAli&i=../trash/hgSs/hgSs_genome_1239_d9e300.pslx+..%2Ftrash%2FhgSs%2FhgSs_genome_1239_d9e300.fa+YourSeq&c=chr2&l=140974738&r=140974897&db=hg19&hgsid=259405959) YourSeq 81 1 162 178 81.6% 2 - 140974739 140974897 159

[browser](http://genome.ucsc.edu/cgi-bin/hgTracks?position=chr21:10492961-10493042&db=hg19&ss=../trash/hgSs/hgSs_genome_1239_d9e300.pslx+../trash/hgSs/hgSs_genome_1239_d9e300.fa&hgsid=259405959) [details](http://genome.ucsc.edu/cgi-bin/hgc?o=10492960&g=htcUserAli&i=../trash/hgSs/hgSs_genome_1239_d9e300.pslx+..%2Ftrash%2FhgSs%2FhgSs_genome_1239_d9e300.fa+YourSeq&c=chr21&l=10492960&r=10493042&db=hg19&hgsid=259405959) YourSeq 78 1 82 178 97.6% 21 + 10492961 10493042 82

[browser](http://genome.ucsc.edu/cgi-bin/hgTracks?position=chrX:55205112-55205229&db=hg19&ss=../trash/hgSs/hgSs_genome_1239_d9e300.pslx+../trash/hgSs/hgSs_genome_1239_d9e300.fa&hgsid=259405959) [details](http://genome.ucsc.edu/cgi-bin/hgc?o=55205111&g=htcUserAli&i=../trash/hgSs/hgSs_genome_1239_d9e300.pslx+..%2Ftrash%2FhgSs%2FhgSs_genome_1239_d9e300.fa+YourSeq&c=chrX&l=55205111&r=55205229&db=hg19&hgsid=259405959) YourSeq 77 33 148 178 84.0% X - 55205112 55205229 118

[browser](http://genome.ucsc.edu/cgi-bin/hgTracks?position=chr9:95301438-95301556&db=hg19&ss=../trash/hgSs/hgSs_genome_1239_d9e300.pslx+../trash/hgSs/hgSs_genome_1239_d9e300.fa&hgsid=259405959) [details](http://genome.ucsc.edu/cgi-bin/hgc?o=95301437&g=htcUserAli&i=../trash/hgSs/hgSs_genome_1239_d9e300.pslx+..%2Ftrash%2FhgSs%2FhgSs_genome_1239_d9e300.fa+YourSeq&c=chr9&l=95301437&r=95301556&db=hg19&hgsid=259405959) YourSeq 75 1 115 178 89.5% 9 - 95301438 95301556 119

[browser](http://genome.ucsc.edu/cgi-bin/hgTracks?position=chr8:111946707-111946854&db=hg19&ss=../trash/hgSs/hgSs_genome_1239_d9e300.pslx+../trash/hgSs/hgSs_genome_1239_d9e300.fa&hgsid=259405959) [details](http://genome.ucsc.edu/cgi-bin/hgc?o=111946706&g=htcUserAli&i=../trash/hgSs/hgSs_genome_1239_d9e300.pslx+..%2Ftrash%2FhgSs%2FhgSs_genome_1239_d9e300.fa+YourSeq&c=chr8&l=111946706&r=111946854&db=hg19&hgsid=259405959) YourSeq 69 1 145 178 87.3% 8 - 111946707 111946854 148

[browser](http://genome.ucsc.edu/cgi-bin/hgTracks?position=chr1:107348605-107348712&db=hg19&ss=../trash/hgSs/hgSs_genome_1239_d9e300.pslx+../trash/hgSs/hgSs_genome_1239_d9e300.fa&hgsid=259405959) [details](http://genome.ucsc.edu/cgi-bin/hgc?o=107348604&g=htcUserAli&i=../trash/hgSs/hgSs_genome_1239_d9e300.pslx+..%2Ftrash%2FhgSs%2FhgSs_genome_1239_d9e300.fa+YourSeq&c=chr1&l=107348604&r=107348712&db=hg19&hgsid=259405959) YourSeq 67 41 148 178 81.8% 1 - 107348605 107348712 108

[browser](http://genome.ucsc.edu/cgi-bin/hgTracks?position=chr8:134768008-134768160&db=hg19&ss=../trash/hgSs/hgSs_genome_1239_d9e300.pslx+../trash/hgSs/hgSs_genome_1239_d9e300.fa&hgsid=259405959) [details](http://genome.ucsc.edu/cgi-bin/hgc?o=134768007&g=htcUserAli&i=../trash/hgSs/hgSs_genome_1239_d9e300.pslx+..%2Ftrash%2FhgSs%2FhgSs_genome_1239_d9e300.fa+YourSeq&c=chr8&l=134768007&r=134768160&db=hg19&hgsid=259405959) YourSeq 67 10 162 178 91.4% 8 + 134768008 134768160 153

[browser](http://genome.ucsc.edu/cgi-bin/hgTracks?position=chr7:141501872-141501949&db=hg19&ss=../trash/hgSs/hgSs_genome_1239_d9e300.pslx+../trash/hgSs/hgSs_genome_1239_d9e300.fa&hgsid=259405959) [details](http://genome.ucsc.edu/cgi-bin/hgc?o=141501871&g=htcUserAli&i=../trash/hgSs/hgSs_genome_1239_d9e300.pslx+..%2Ftrash%2FhgSs%2FhgSs_genome_1239_d9e300.fa+YourSeq&c=chr7&l=141501871&r=141501949&db=hg19&hgsid=259405959) YourSeq 63 1 78 178 93.1% 7 - 141501872 141501949 78

[browser](http://genome.ucsc.edu/cgi-bin/hgTracks?position=chr11:103276576-103276653&db=hg19&ss=../trash/hgSs/hgSs_genome_1239_d9e300.pslx+../trash/hgSs/hgSs_genome_1239_d9e300.fa&hgsid=259405959) [details](http://genome.ucsc.edu/cgi-bin/hgc?o=103276575&g=htcUserAli&i=../trash/hgSs/hgSs_genome_1239_d9e300.pslx+..%2Ftrash%2FhgSs%2FhgSs_genome_1239_d9e300.fa+YourSeq&c=chr11&l=103276575&r=103276653&db=hg19&hgsid=259405959) YourSeq 61 1 78 178 91.7% 11 - 103276576 103276653 78

[browser](http://genome.ucsc.edu/cgi-bin/hgTracks?position=chr7:63570541-63570618&db=hg19&ss=../trash/hgSs/hgSs_genome_1239_d9e300.pslx+../trash/hgSs/hgSs_genome_1239_d9e300.fa&hgsid=259405959) [details](http://genome.ucsc.edu/cgi-bin/hgc?o=63570540&g=htcUserAli&i=../trash/hgSs/hgSs_genome_1239_d9e300.pslx+..%2Ftrash%2FhgSs%2FhgSs_genome_1239_d9e300.fa+YourSeq&c=chr7&l=63570540&r=63570618&db=hg19&hgsid=259405959) YourSeq 59 1 78 178 90.3% 7 - 63570541 63570618 78

[browser](http://genome.ucsc.edu/cgi-bin/hgTracks?position=chr2:156120290-156120365&db=hg19&ss=../trash/hgSs/hgSs_genome_1239_d9e300.pslx+../trash/hgSs/hgSs_genome_1239_d9e300.fa&hgsid=259405959) [details](http://genome.ucsc.edu/cgi-bin/hgc?o=156120289&g=htcUserAli&i=../trash/hgSs/hgSs_genome_1239_d9e300.pslx+..%2Ftrash%2FhgSs%2FhgSs_genome_1239_d9e300.fa+YourSeq&c=chr2&l=156120289&r=156120365&db=hg19&hgsid=259405959) YourSeq 58 1 76 178 88.2% 2 - 156120290 156120365 76

[browser](http://genome.ucsc.edu/cgi-bin/hgTracks?position=chr2:131031388-131031464&db=hg19&ss=../trash/hgSs/hgSs_genome_1239_d9e300.pslx+../trash/hgSs/hgSs_genome_1239_d9e300.fa&hgsid=259405959) [details](http://genome.ucsc.edu/cgi-bin/hgc?o=131031387&g=htcUserAli&i=../trash/hgSs/hgSs_genome_1239_d9e300.pslx+..%2Ftrash%2FhgSs%2FhgSs_genome_1239_d9e300.fa+YourSeq&c=chr2&l=131031387&r=131031464&db=hg19&hgsid=259405959) YourSeq 58 1 78 178 88.3% 2 + 131031388 131031464 77

[browser](http://genome.ucsc.edu/cgi-bin/hgTracks?position=chr10:71352697-71352773&db=hg19&ss=../trash/hgSs/hgSs_genome_1239_d9e300.pslx+../trash/hgSs/hgSs_genome_1239_d9e300.fa&hgsid=259405959) [details](http://genome.ucsc.edu/cgi-bin/hgc?o=71352696&g=htcUserAli&i=../trash/hgSs/hgSs_genome_1239_d9e300.pslx+..%2Ftrash%2FhgSs%2FhgSs_genome_1239_d9e300.fa+YourSeq&c=chr10&l=71352696&r=71352773&db=hg19&hgsid=259405959) YourSeq 55 1 77 178 92.4% 10 - 71352697 71352773 77

[browser](http://genome.ucsc.edu/cgi-bin/hgTracks?position=chr1:238104374-238104450&db=hg19&ss=../trash/hgSs/hgSs_genome_1239_d9e300.pslx+../trash/hgSs/hgSs_genome_1239_d9e300.fa&hgsid=259405959) [details](http://genome.ucsc.edu/cgi-bin/hgc?o=238104373&g=htcUserAli&i=../trash/hgSs/hgSs_genome_1239_d9e300.pslx+..%2Ftrash%2FhgSs%2FhgSs_genome_1239_d9e300.fa+YourSeq&c=chr1&l=238104373&r=238104450&db=hg19&hgsid=259405959) YourSeq 55 1 77 178 89.8% 1 - 238104374 238104450 77

[browser](http://genome.ucsc.edu/cgi-bin/hgTracks?position=chr10:127508432-127508506&db=hg19&ss=../trash/hgSs/hgSs_genome_1239_d9e300.pslx+../trash/hgSs/hgSs_genome_1239_d9e300.fa&hgsid=259405959) [details](http://genome.ucsc.edu/cgi-bin/hgc?o=127508431&g=htcUserAli&i=../trash/hgSs/hgSs_genome_1239_d9e300.pslx+..%2Ftrash%2FhgSs%2FhgSs_genome_1239_d9e300.fa+YourSeq&c=chr10&l=127508431&r=127508506&db=hg19&hgsid=259405959) YourSeq 52 1 77 178 86.0% 10 + 127508432 127508506 75

[browser](http://genome.ucsc.edu/cgi-bin/hgTracks?position=chr10:81170992-81171049&db=hg19&ss=../trash/hgSs/hgSs_genome_1239_d9e300.pslx+../trash/hgSs/hgSs_genome_1239_d9e300.fa&hgsid=259405959) [details](http://genome.ucsc.edu/cgi-bin/hgc?o=81170991&g=htcUserAli&i=../trash/hgSs/hgSs_genome_1239_d9e300.pslx+..%2Ftrash%2FhgSs%2FhgSs_genome_1239_d9e300.fa+YourSeq&c=chr10&l=81170991&r=81171049&db=hg19&hgsid=259405959) YourSeq 46 1 57 178 94.3% 10 + 81170992 81171049 58

[browser](http://genome.ucsc.edu/cgi-bin/hgTracks?position=chr7:57255477-57255541&db=hg19&ss=../trash/hgSs/hgSs_genome_1239_d9e300.pslx+../trash/hgSs/hgSs_genome_1239_d9e300.fa&hgsid=259405959) [details](http://genome.ucsc.edu/cgi-bin/hgc?o=57255476&g=htcUserAli&i=../trash/hgSs/hgSs_genome_1239_d9e300.pslx+..%2Ftrash%2FhgSs%2FhgSs_genome_1239_d9e300.fa+YourSeq&c=chr7&l=57255476&r=57255541&db=hg19&hgsid=259405959) YourSeq 44 15 79 178 94.0% 7 + 57255477 57255541 65

[browser](http://genome.ucsc.edu/cgi-bin/hgTracks?position=chr2:95566381-95566522&db=hg19&ss=../trash/hgSs/hgSs_genome_1239_d9e300.pslx+../trash/hgSs/hgSs_genome_1239_d9e300.fa&hgsid=259405959) [details](http://genome.ucsc.edu/cgi-bin/hgc?o=95566380&g=htcUserAli&i=../trash/hgSs/hgSs_genome_1239_d9e300.pslx+..%2Ftrash%2FhgSs%2FhgSs_genome_1239_d9e300.fa+YourSeq&c=chr2&l=95566380&r=95566522&db=hg19&hgsid=259405959) YourSeq 36 11 115 178 95.0% 2 - 95566381 95566522 142

[browser](http://genome.ucsc.edu/cgi-bin/hgTracks?position=chr16:3419262-3419297&db=hg19&ss=../trash/hgSs/hgSs_genome_1239_d9e300.pslx+../trash/hgSs/hgSs_genome_1239_d9e300.fa&hgsid=259405959) [details](http://genome.ucsc.edu/cgi-bin/hgc?o=3419261&g=htcUserAli&i=../trash/hgSs/hgSs_genome_1239_d9e300.pslx+..%2Ftrash%2FhgSs%2FhgSs_genome_1239_d9e300.fa+YourSeq&c=chr16&l=3419261&r=3419297&db=hg19&hgsid=259405959) YourSeq 33 41 77 178 88.9% 16 - 3419262 3419297 36

[browser](http://genome.ucsc.edu/cgi-bin/hgTracks?position=chr2:132141704-132141727&db=hg19&ss=../trash/hgSs/hgSs_genome_1239_d9e300.pslx+../trash/hgSs/hgSs_genome_1239_d9e300.fa&hgsid=259405959) [details](http://genome.ucsc.edu/cgi-bin/hgc?o=132141703&g=htcUserAli&i=../trash/hgSs/hgSs_genome_1239_d9e300.pslx+..%2Ftrash%2FhgSs%2FhgSs_genome_1239_d9e300.fa+YourSeq&c=chr2&l=132141703&r=132141727&db=hg19&hgsid=259405959) YourSeq 22 1 24 178 95.9% 2 - 132141704 132141727 24

[browser](http://genome.ucsc.edu/cgi-bin/hgTracks?position=chr3:192598557-192598576&db=hg19&ss=../trash/hgSs/hgSs_genome_1239_d9e300.pslx+../trash/hgSs/hgSs_genome_1239_d9e300.fa&hgsid=259405959) [details](http://genome.ucsc.edu/cgi-bin/hgc?o=192598556&g=htcUserAli&i=../trash/hgSs/hgSs_genome_1239_d9e300.pslx+..%2Ftrash%2FhgSs%2FhgSs_genome_1239_d9e300.fa+YourSeq&c=chr3&l=192598556&r=192598576&db=hg19&hgsid=259405959) YourSeq 20 32 51 178 100.0% 3 + 192598557 192598576 20

**Reads BLAT results**

7:chrM:5864-5964(-)

AGTCCAATGCTTCACTCAGCCATTTTACCTCACCCCCACTGAT**T**TTCGCCGACCGTTGACTATTCTCTACAAACCACAAAGACATTGGAACACTATACCTA

## chr1:566413-566513

AGTCCAATGCTTCACTCAGCCATTTTACCTCACCCCCACTGATgTTCGCCGACCGTTGACTATTCTCTACAAACCACAAAGACATTGGAACACTATACCTA

## chr14:32953790-32954046

AGTCCAATGCTTCACTCAGCCATTTTACCTtatCCCACTGATgTTCGCCGACCGTTGAtTgTTCTCTACAAACCACAAAGACATTGGAACACT

8: chrM:5838-5938(-)

GGCCTAACCCCTGTCTTTAGATTTACAGTCCAATGCTTCACTCAGCCATTTTACCTCACCCCCACTGAT**A**TTCGCCGACCGTTGACTATTCTCTACAAACC

## chr1:566387-566487

GGCtTAACCCCTGTCTTTAGATTTACAGTCCAATGCTTCACTCAGCCATTTTACCTCACCCCCACTGATgTTCGCCGACCGTTGACTATTCTCTACAAACC

## chr14:32953973-32954065

CCCCTGTCTTTAGATTTACAGTCCAATGCTTCACTCAGCCATTTTACCTtatCCCACTGATgTTCGCCGACCGTTGAtTgTTCTCTACAAACC

**Primers BLAST results**

>[NT_004350.19](http://www.ncbi.nlm.nih.gov/entrez/viewer.fcgi?db=nucleotide&id=224514624) Homo sapiens chromosome 1 genomic contig, GRCh37.p5 Primary Assembly

product length = 178

Forward primer 1 CACCTCGGAGCTGGTAAAAA 20

Template 44997 ......A............. 45016

Reverse primer 1 CCTAGGACTCCAGCTCATGC 20

Template 45174 .................... 45155

>[NT_026437.12](http://www.ncbi.nlm.nih.gov/entrez/viewer.fcgi?db=nucleotide&id=224514933) Homo sapiens chromosome 14 genomic contig, GRCh37.p5 Primary Assembly

product length = 177

Forward primer 1 CACCTCGGAGCTGGTAAAAA 20

Template 13954094 ......A............. 13954075

Reverse primer 1 CCTAGGACTCCAGCTCATGC 20

Template 13953918 ..C................. 13953937

>[NT_024862.14](http://www.ncbi.nlm.nih.gov/entrez/viewer.fcgi?db=nucleotide&id=224514947) Homo sapiens chromosome 17 genomic contig, GRCh37.p5 Primary Assembly

product length = 161

Forward primer 1 CACCTCGGAGCTGGTAAAAA 20

Template 359958 ......A.G........... 359977

Reverse primer 1 CCTAGGACTCCAGCTCATGC 20

Template 360118 ..C.A............... 360099

>[NT_05403.17](http://www.ncbi.nlm.nih.gov/entrez/viewer.fcgi?db=nucleotide&id=224514625) Homo sapiens chromosome 2 genomic contig, GRCh37.p5 Primary Assembly

product length = 185

Forward primer 1 CACCTCGGAGCTGGTAAAAA 20

Template 6329783 ......A.G........... 6329764

Reverse primer 1 CCTAGGACTCCAGCTCATGC 20

Template 6329599 ........C........G.. 6329618

>[NT_021937.19](http://www.ncbi.nlm.nih.gov/entrez/viewer.fcgi?db=nucleotide&id=224514681) Homo sapiens chromosome 1 genomic contig, GRCh37.p5 Primary Assembly

product length = 4690

Reverse primer 1 CCTAGGACTCCAGCTCATGC 20

Template 1571435 ........G...C...C... 1571416

Reverse primer 1 CCTAGGACTCCAGCTCATGC 20

Template 1566746 AA.C...A............ 1566765

**Numts aligned to the variant on the UCSC genome browser**

37 Numts: 2A


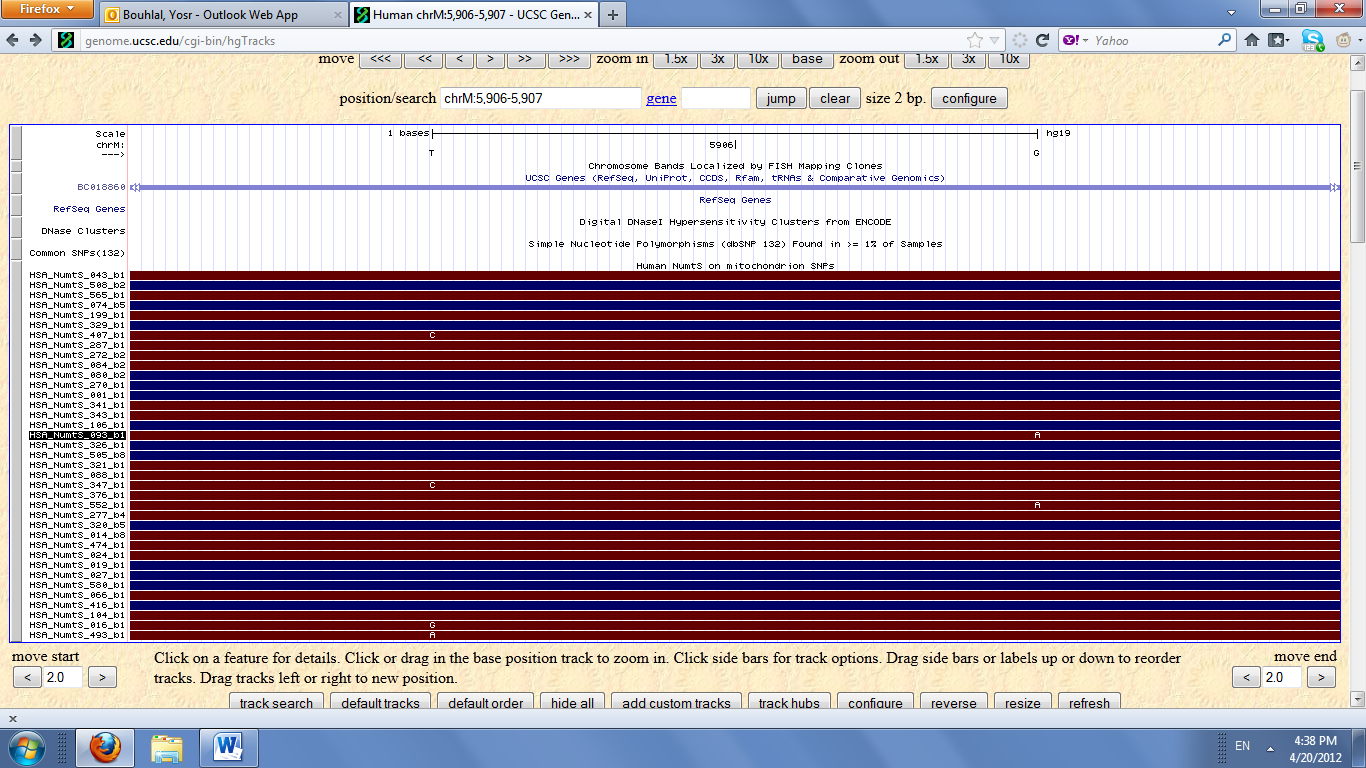


**Variant6: m.6569C>A**

**Template Fragment**

**CTTAGGGGCCATCAATTTCA**TCACAACAATTATCAATATAAAACCCCCTGCCATAACCCAATACCAAACGCCCCTCTTCGTCTGATCCGTCCTAATCACAGCAGTCCTACTTCTCCTATCTCTCCCAGTCCTAGCTGCTGGCATCACTATACTACTAACAGACCGCAACCTCAACACCACCTTCTTCGACCC**C**GCCGGAGGAGGAGACCCCATTCTATACCAACACCTA**TTCTGATTTTTCGGTCACCC**

**Template BLAT results**

ACTIONS QUERY SCORE START END QSIZE IDENTITY CHRO STRAND START END SPAN

---------------------------------------------------------------------------------------------------

[browser](http://genome.ucsc.edu/cgi-bin/hgTracks?position=chrM:6378-6626&db=hg19&ss=../trash/hgSs/hgSs_genome_5370_c53520.pslx+../trash/hgSs/hgSs_genome_5370_c53520.fa&hgsid=258719997) [details](http://genome.ucsc.edu/cgi-bin/hgc?o=6377&g=htcUserAli&i=../trash/hgSs/hgSs_genome_5370_c53520.pslx+..%2Ftrash%2FhgSs%2FhgSs_genome_5370_c53520.fa+YourSeq&c=chrM&l=6377&r=6626&db=hg19&hgsid=258719997) YourSeq 249 1 249 249 100.0% M + 6378 6626 249

[browser](http://genome.ucsc.edu/cgi-bin/hgTracks?position=chr1:566927-567175&db=hg19&ss=../trash/hgSs/hgSs_genome_5370_c53520.pslx+../trash/hgSs/hgSs_genome_5370_c53520.fa&hgsid=258719997) [details](http://genome.ucsc.edu/cgi-bin/hgc?o=566926&g=htcUserAli&i=../trash/hgSs/hgSs_genome_5370_c53520.pslx+..%2Ftrash%2FhgSs%2FhgSs_genome_5370_c53520.fa+YourSeq&c=chr1&l=566926&r=567175&db=hg19&hgsid=258719997) YourSeq 235 1 249 249 97.2% 1 + 566927 567175 249

[browser](http://genome.ucsc.edu/cgi-bin/hgTracks?position=chr5:99390214-99390437&db=hg19&ss=../trash/hgSs/hgSs_genome_5370_c53520.pslx+../trash/hgSs/hgSs_genome_5370_c53520.fa&hgsid=258719997) [details](http://genome.ucsc.edu/cgi-bin/hgc?o=99390213&g=htcUserAli&i=../trash/hgSs/hgSs_genome_5370_c53520.pslx+..%2Ftrash%2FhgSs%2FhgSs_genome_5370_c53520.fa+YourSeq&c=chr5&l=99390213&r=99390437&db=hg19&hgsid=258719997) YourSeq 186 17 240 249 91.6% 5 - 99390214 99390437 224

[browser](http://genome.ucsc.edu/cgi-bin/hgTracks?position=chr14:32953304-32953517&db=hg19&ss=../trash/hgSs/hgSs_genome_5370_c53520.pslx+../trash/hgSs/hgSs_genome_5370_c53520.fa&hgsid=258719997) [details](http://genome.ucsc.edu/cgi-bin/hgc?o=32953303&g=htcUserAli&i=../trash/hgSs/hgSs_genome_5370_c53520.pslx+..%2Ftrash%2FhgSs%2FhgSs_genome_5370_c53520.fa+YourSeq&c=chr14&l=32953303&r=32953517&db=hg19&hgsid=258719997) YourSeq 178 17 230 249 91.6% 14 - 32953304 32953517 214

[browser](http://genome.ucsc.edu/cgi-bin/hgTracks?position=chr2:50816199-50816421&db=hg19&ss=../trash/hgSs/hgSs_genome_5370_c53520.pslx+../trash/hgSs/hgSs_genome_5370_c53520.fa&hgsid=258719997) [details](http://genome.ucsc.edu/cgi-bin/hgc?o=50816198&g=htcUserAli&i=../trash/hgSs/hgSs_genome_5370_c53520.pslx+..%2Ftrash%2FhgSs%2FhgSs_genome_5370_c53520.fa+YourSeq&c=chr2&l=50816198&r=50816421&db=hg19&hgsid=258719997) YourSeq 161 26 249 249 86.2% 2 - 50816199 50816421 223

[browser](http://genome.ucsc.edu/cgi-bin/hgTracks?position=chr17:22027124-22027358&db=hg19&ss=../trash/hgSs/hgSs_genome_5370_c53520.pslx+../trash/hgSs/hgSs_genome_5370_c53520.fa&hgsid=258719997) [details](http://genome.ucsc.edu/cgi-bin/hgc?o=22027123&g=htcUserAli&i=../trash/hgSs/hgSs_genome_5370_c53520.pslx+..%2Ftrash%2FhgSs%2FhgSs_genome_5370_c53520.fa+YourSeq&c=chr17&l=22027123&r=22027358&db=hg19&hgsid=258719997) YourSeq 151 14 249 249 87.7% 17 + 22027124 22027358 235

[browser](http://genome.ucsc.edu/cgi-bin/hgTracks?position=chr2:132141112-132141253&db=hg19&ss=../trash/hgSs/hgSs_genome_5370_c53520.pslx+../trash/hgSs/hgSs_genome_5370_c53520.fa&hgsid=258719997) [details](http://genome.ucsc.edu/cgi-bin/hgc?o=132141111&g=htcUserAli&i=../trash/hgSs/hgSs_genome_5370_c53520.pslx+..%2Ftrash%2FhgSs%2FhgSs_genome_5370_c53520.fa+YourSeq&c=chr2&l=132141111&r=132141253&db=hg19&hgsid=258719997) YourSeq 99 26 168 249 83.1% 2 - 132141112 132141253 142

[browser](http://genome.ucsc.edu/cgi-bin/hgTracks?position=chr7:63569935-63570065&db=hg19&ss=../trash/hgSs/hgSs_genome_5370_c53520.pslx+../trash/hgSs/hgSs_genome_5370_c53520.fa&hgsid=258719997) [details](http://genome.ucsc.edu/cgi-bin/hgc?o=63569934&g=htcUserAli&i=../trash/hgSs/hgSs_genome_5370_c53520.pslx+..%2Ftrash%2FhgSs%2FhgSs_genome_5370_c53520.fa+YourSeq&c=chr7&l=63569934&r=63570065&db=hg19&hgsid=258719997) YourSeq 88 2 135 249 80.2% 7 - 63569935 63570065 131

[browser](http://genome.ucsc.edu/cgi-bin/hgTracks?position=chrY:13289501-13289626&db=hg19&ss=../trash/hgSs/hgSs_genome_5370_c53520.pslx+../trash/hgSs/hgSs_genome_5370_c53520.fa&hgsid=258719997) [details](http://genome.ucsc.edu/cgi-bin/hgc?o=13289500&g=htcUserAli&i=../trash/hgSs/hgSs_genome_5370_c53520.pslx+..%2Ftrash%2FhgSs%2FhgSs_genome_5370_c53520.fa+YourSeq&c=chrY&l=13289500&r=13289626&db=hg19&hgsid=258719997) YourSeq 84 26 151 249 83.4% Y + 13289501 13289626 126

[browser](http://genome.ucsc.edu/cgi-bin/hgTracks?position=chr2:131032034-131032129&db=hg19&ss=../trash/hgSs/hgSs_genome_5370_c53520.pslx+../trash/hgSs/hgSs_genome_5370_c53520.fa&hgsid=258719997) [details](http://genome.ucsc.edu/cgi-bin/hgc?o=131032033&g=htcUserAli&i=../trash/hgSs/hgSs_genome_5370_c53520.pslx+..%2Ftrash%2FhgSs%2FhgSs_genome_5370_c53520.fa+YourSeq&c=chr2&l=131032033&r=131032129&db=hg19&hgsid=258719997) YourSeq 70 73 168 249 86.5% 2 + 131032034 131032129 96

[browser](http://genome.ucsc.edu/cgi-bin/hgTracks?position=chr3:96484038-96484128&db=hg19&ss=../trash/hgSs/hgSs_genome_5370_c53520.pslx+../trash/hgSs/hgSs_genome_5370_c53520.fa&hgsid=258719997) [details](http://genome.ucsc.edu/cgi-bin/hgc?o=96484037&g=htcUserAli&i=../trash/hgSs/hgSs_genome_5370_c53520.pslx+..%2Ftrash%2FhgSs%2FhgSs_genome_5370_c53520.fa+YourSeq&c=chr3&l=96484037&r=96484128&db=hg19&hgsid=258719997) YourSeq 67 159 249 249 86.9% 3 - 96484038 96484128 91

[browser](http://genome.ucsc.edu/cgi-bin/hgTracks?position=chrY:8979505-8979570&db=hg19&ss=../trash/hgSs/hgSs_genome_5370_c53520.pslx+../trash/hgSs/hgSs_genome_5370_c53520.fa&hgsid=258719997) [details](http://genome.ucsc.edu/cgi-bin/hgc?o=8979504&g=htcUserAli&i=../trash/hgSs/hgSs_genome_5370_c53520.pslx+..%2Ftrash%2FhgSs%2FhgSs_genome_5370_c53520.fa+YourSeq&c=chrY&l=8979504&r=8979570&db=hg19&hgsid=258719997) YourSeq 66 123 188 249 100.0% Y + 8979505 8979570 66

[browser](http://genome.ucsc.edu/cgi-bin/hgTracks?position=chr7:57256145-57256218&db=hg19&ss=../trash/hgSs/hgSs_genome_5370_c53520.pslx+../trash/hgSs/hgSs_genome_5370_c53520.fa&hgsid=258719997) [details](http://genome.ucsc.edu/cgi-bin/hgc?o=57256144&g=htcUserAli&i=../trash/hgSs/hgSs_genome_5370_c53520.pslx+..%2Ftrash%2FhgSs%2FhgSs_genome_5370_c53520.fa+YourSeq&c=chr7&l=57256144&r=57256218&db=hg19&hgsid=258719997) YourSeq 57 71 144 249 90.2% 7 + 57256145 57256218 74

[browser](http://genome.ucsc.edu/cgi-bin/hgTracks?position=chrX:125605687-125605750&db=hg19&ss=../trash/hgSs/hgSs_genome_5370_c53520.pslx+../trash/hgSs/hgSs_genome_5370_c53520.fa&hgsid=258719997) [details](http://genome.ucsc.edu/cgi-bin/hgc?o=125605686&g=htcUserAli&i=../trash/hgSs/hgSs_genome_5370_c53520.pslx+..%2Ftrash%2FhgSs%2FhgSs_genome_5370_c53520.fa+YourSeq&c=chrX&l=125605686&r=125605750&db=hg19&hgsid=258719997) YourSeq 56 177 240 249 93.8% X + 125605687 125605750 64

[browser](http://genome.ucsc.edu/cgi-bin/hgTracks?position=chr1:142791956-142792035&db=hg19&ss=../trash/hgSs/hgSs_genome_5370_c53520.pslx+../trash/hgSs/hgSs_genome_5370_c53520.fa&hgsid=258719997) [details](http://genome.ucsc.edu/cgi-bin/hgc?o=142791955&g=htcUserAli&i=../trash/hgSs/hgSs_genome_5370_c53520.pslx+..%2Ftrash%2FhgSs%2FhgSs_genome_5370_c53520.fa+YourSeq&c=chr1&l=142791955&r=142792035&db=hg19&hgsid=258719997) YourSeq 56 26 105 249 85.0% 1 + 142791956 142792035 80

[browser](http://genome.ucsc.edu/cgi-bin/hgTracks?position=chr2:167271014-167271039&db=hg19&ss=../trash/hgSs/hgSs_genome_5370_c53520.pslx+../trash/hgSs/hgSs_genome_5370_c53520.fa&hgsid=258719997) [details](http://genome.ucsc.edu/cgi-bin/hgc?o=167271013&g=htcUserAli&i=../trash/hgSs/hgSs_genome_5370_c53520.pslx+..%2Ftrash%2FhgSs%2FhgSs_genome_5370_c53520.fa+YourSeq&c=chr2&l=167271013&r=167271039&db=hg19&hgsid=258719997) YourSeq 26 224 249 249 100.0% 2 + 167271014 167271039 26

[browser](http://genome.ucsc.edu/cgi-bin/hgTracks?position=chr7:141501250-141501272&db=hg19&ss=../trash/hgSs/hgSs_genome_5370_c53520.pslx+../trash/hgSs/hgSs_genome_5370_c53520.fa&hgsid=258719997) [details](http://genome.ucsc.edu/cgi-bin/hgc?o=141501249&g=htcUserAli&i=../trash/hgSs/hgSs_genome_5370_c53520.pslx+..%2Ftrash%2FhgSs%2FhgSs_genome_5370_c53520.fa+YourSeq&c=chr7&l=141501249&r=141501272&db=hg19&hgsid=258719997) YourSeq 21 113 135 249 95.7% 7 - 141501250 141501272 23

[browser](http://genome.ucsc.edu/cgi-bin/hgTracks?position=chr4:162370810-162370829&db=hg19&ss=../trash/hgSs/hgSs_genome_5370_c53520.pslx+../trash/hgSs/hgSs_genome_5370_c53520.fa&hgsid=258719997) [details](http://genome.ucsc.edu/cgi-bin/hgc?o=162370809&g=htcUserAli&i=../trash/hgSs/hgSs_genome_5370_c53520.pslx+..%2Ftrash%2FhgSs%2FhgSs_genome_5370_c53520.fa+YourSeq&c=chr4&l=162370809&r=162370829&db=hg19&hgsid=258719997) YourSeq 20 110 129 249 100.0% 4 - 162370810 162370829 20

**Reads BLAT results**

7: chrM:6562-6662 (+)

TTCGACCC**A**GCCGGAGGAGGAGACCCCATTCTATACCAACACCTATTCTGATTTTTCGGTCACCCTGAAGTTTATATTCTCATCCTACCAGGCTTCGGAAT

## chr1:567111-567211

TTCGACCCAGCCGGAGGAGGAGACCCCATTCTATACCAACACCTATTCTGATTTTTCGGTCACCCTGAAGTTTATATTCTCATCCTACCAGGCTTCGGAAT

## chrX:125605695-125605795

TTCGACCCAGCCGGgGGAGGgGACCCtATTCTATACCAACACCTATTCTGATTTTTtGGcCACCCcGAAGTTTATATTCTtATCCTgCCAGGCTTCGGAAT

8: chrM:6528-6628 (+)

ACTACTAACAGACCGTAACCTCAACACCACCTTCTTCGACCC**A**GCCGGAGGAGGAGACCCCATTCTATACCAACACCTATTCTGATTTTTCGGTCACCCTG

## chr1:567077-567177

ACTACTAACAGACCGTAACCTCAACACCACCTTCTTCGACCCAGCCGGAGGAGGAGACCCCATTCTATACCAACACCTATTCTGATTTTTCGGTCACCCTG

**Primers BLAST results**

>[NT_004350.19](http://www.ncbi.nlm.nih.gov/entrez/viewer.fcgi?db=nucleotide&id=224514624) Homo sapiens chromosome 1 genomic contig, GRCh37.p5 Primary Assembly

product length = 249

Forward primer 1 CTTAGGGGCCATCAATTTCA 20

Template 45559 ......A............. 45578

Reverse primer 1 GGGTGACCGAAAAATCAGAA 20

Template 45807 .................... 45788

>[NT_008470.19](http://www.ncbi.nlm.nih.gov/entrez/viewer.fcgi?db=nucleotide&id=224514751) Homo sapiens chromosome 9 genomic contig, GRCh37.p5 Primary Assembly

product length = 246

Forward primer 1 CTTAGGGGCCATCAATTTCA 20

Template 12343605 T...........T..C.... 12343586

Reverse primer 1 GGGTGACCGAAAAATCAGAA 20

Template 12343360 ........A..G........ 12343379

>[NT_016354.19](http://www.ncbi.nlm.nih.gov/entrez/viewer.fcgi?db=nucleotide&id=224514665) Homo sapiens chromosome 4 genomic contig, GRCh37.p5 Primary Assembly

product length = 246

Forward primer 1 CTTAGGGGCCATCAATTTCA 20

Template 80929637 T...........T.....T. 80929618

Reverse primer 1 GGGTGACCGAAAAATCAGAA 20

Template 80929392 ...C....A..G........ 80929411

>[NT_007914.15](http://www.ncbi.nlm.nih.gov/entrez/viewer.fcgi?db=nucleotide&id=224514812) Homo sapiens chromosome 7 genomic contig, GRCh37.p5 Primary Assembly

product length = 251

Forward primer 1 CTTAGGGGCCATCAATTTCA 20

Template 2097008 T...........T.....T. 2096989

Reverse primer 1 GGGTGACCGAAAAATCAGAA 20

Template 2096758 TT......A..G........ 2096777

**Numts aligned to the variant on the UCSC genome browser**


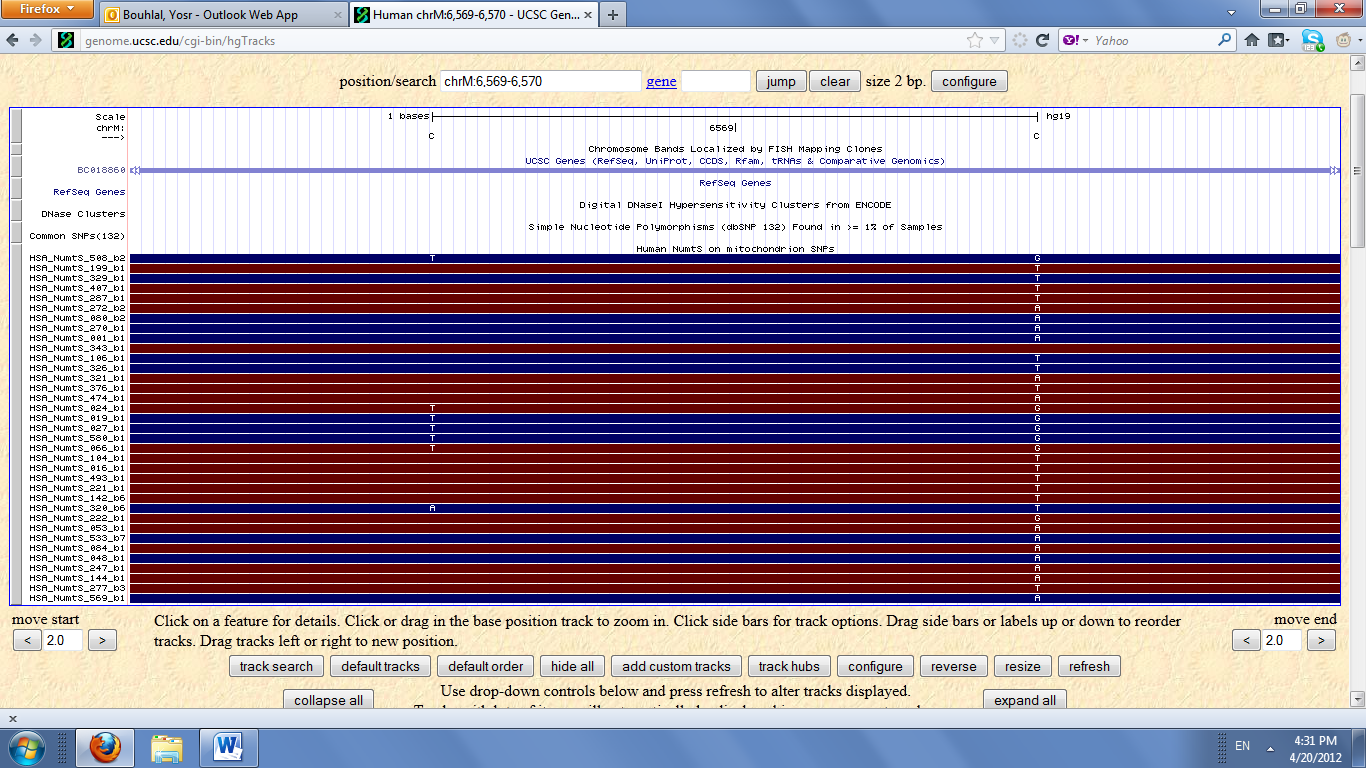


Numts: 35 : 7G, 14T,13A

**Variant7: m.6998C>A**

**Template Fragment**

**CCGGCGTCAAAGTATTTAGC**TGACTCGCCACACTCCACGGAAGCAATATGAAATGATCTGCTGCAGTGCTCTGAGCCCTAGGATTCATCTTTCTTTTCACCGTAGGTGGCCTGACTGGCATTGTATTAGCAAACTCATCACTAGACAT**C**GTACTACACGACACGTACTACGTTGTAGCCCACTTCCACTATGTCCTATCAATAGGAGCTGTA**TTTGCCATCATAGGAGGCT**

**Template BLAT results**

ACTIONS QUERY SCORE START END QSIZE IDENTITY CHRO STRAND START END SPAN

---------------------------------------------------------------------------------------------------

[browser](http://genome.ucsc.edu/cgi-bin/hgTracks?position=chrM:6851-7082&db=hg19&ss=../trash/hgSs/hgSs_genome_7981_ca98f0.pslx+../trash/hgSs/hgSs_genome_7981_ca98f0.fa&hgsid=258719997) [details](http://genome.ucsc.edu/cgi-bin/hgc?o=6850&g=htcUserAli&i=../trash/hgSs/hgSs_genome_7981_ca98f0.pslx+..%2Ftrash%2FhgSs%2FhgSs_genome_7981_ca98f0.fa+YourSeq&c=chrM&l=6850&r=7082&db=hg19&hgsid=258719997) YourSeq 230 1 232 232 99.6% M + 6851 7082 232

[browser](http://genome.ucsc.edu/cgi-bin/hgTracks?position=chr1:567401-567632&db=hg19&ss=../trash/hgSs/hgSs_genome_7981_ca98f0.pslx+../trash/hgSs/hgSs_genome_7981_ca98f0.fa&hgsid=258719997) [details](http://genome.ucsc.edu/cgi-bin/hgc?o=567400&g=htcUserAli&i=../trash/hgSs/hgSs_genome_7981_ca98f0.pslx+..%2Ftrash%2FhgSs%2FhgSs_genome_7981_ca98f0.fa+YourSeq&c=chr1&l=567400&r=567632&db=hg19&hgsid=258719997) YourSeq 228 1 232 232 99.2% 1 + 567401 567632 232

[browser](http://genome.ucsc.edu/cgi-bin/hgTracks?position=chr17:51183126-51183357&db=hg19&ss=../trash/hgSs/hgSs_genome_7981_ca98f0.pslx+../trash/hgSs/hgSs_genome_7981_ca98f0.fa&hgsid=258719997) [details](http://genome.ucsc.edu/cgi-bin/hgc?o=51183125&g=htcUserAli&i=../trash/hgSs/hgSs_genome_7981_ca98f0.pslx+..%2Ftrash%2FhgSs%2FhgSs_genome_7981_ca98f0.fa+YourSeq&c=chr17&l=51183125&r=51183357&db=hg19&hgsid=258719997) YourSeq 206 1 232 232 94.4% 17 + 51183126 51183357 232

[browser](http://genome.ucsc.edu/cgi-bin/hgTracks?position=chrX:125605984-125606215&db=hg19&ss=../trash/hgSs/hgSs_genome_7981_ca98f0.pslx+../trash/hgSs/hgSs_genome_7981_ca98f0.fa&hgsid=258719997) [details](http://genome.ucsc.edu/cgi-bin/hgc?o=125605983&g=htcUserAli&i=../trash/hgSs/hgSs_genome_7981_ca98f0.pslx+..%2Ftrash%2FhgSs%2FhgSs_genome_7981_ca98f0.fa+YourSeq&c=chrX&l=125605983&r=125606215&db=hg19&hgsid=258719997) YourSeq 190 1 232 232 91.0% X + 125605984 125606215 232

[browser](http://genome.ucsc.edu/cgi-bin/hgTracks?position=chr5:99389754-99389979&db=hg19&ss=../trash/hgSs/hgSs_genome_7981_ca98f0.pslx+../trash/hgSs/hgSs_genome_7981_ca98f0.fa&hgsid=258719997) [details](http://genome.ucsc.edu/cgi-bin/hgc?o=99389753&g=htcUserAli&i=../trash/hgSs/hgSs_genome_7981_ca98f0.pslx+..%2Ftrash%2FhgSs%2FhgSs_genome_7981_ca98f0.fa+YourSeq&c=chr5&l=99389753&r=99389979&db=hg19&hgsid=258719997) YourSeq 178 1 226 232 89.4% 5 - 99389754 99389979 226

[browser](http://genome.ucsc.edu/cgi-bin/hgTracks?position=chr17:22027583-22027887&db=hg19&ss=../trash/hgSs/hgSs_genome_7981_ca98f0.pslx+../trash/hgSs/hgSs_genome_7981_ca98f0.fa&hgsid=258719997) [details](http://genome.ucsc.edu/cgi-bin/hgc?o=22027582&g=htcUserAli&i=../trash/hgSs/hgSs_genome_7981_ca98f0.pslx+..%2Ftrash%2FhgSs%2FhgSs_genome_7981_ca98f0.fa+YourSeq&c=chr17&l=22027582&r=22027887&db=hg19&hgsid=258719997) YourSeq 153 1 224 232 84.4% 17 + 22027583 22027887 305

[browser](http://genome.ucsc.edu/cgi-bin/hgTracks?position=chr2:49456767-49456929&db=hg19&ss=../trash/hgSs/hgSs_genome_7981_ca98f0.pslx+../trash/hgSs/hgSs_genome_7981_ca98f0.fa&hgsid=258719997) [details](http://genome.ucsc.edu/cgi-bin/hgc?o=49456766&g=htcUserAli&i=../trash/hgSs/hgSs_genome_7981_ca98f0.pslx+..%2Ftrash%2FhgSs%2FhgSs_genome_7981_ca98f0.fa+YourSeq&c=chr2&l=49456766&r=49456929&db=hg19&hgsid=258719997) YourSeq 143 1 163 232 93.9% 2 - 49456767 49456929 163

[browser](http://genome.ucsc.edu/cgi-bin/hgTracks?position=chr3:89638202-89638432&db=hg19&ss=../trash/hgSs/hgSs_genome_7981_ca98f0.pslx+../trash/hgSs/hgSs_genome_7981_ca98f0.fa&hgsid=258719997) [details](http://genome.ucsc.edu/cgi-bin/hgc?o=89638201&g=htcUserAli&i=../trash/hgSs/hgSs_genome_7981_ca98f0.pslx+..%2Ftrash%2FhgSs%2FhgSs_genome_7981_ca98f0.fa+YourSeq&c=chr3&l=89638201&r=89638432&db=hg19&hgsid=258719997) YourSeq 140 1 232 232 79.3% 3 - 89638202 89638432 231

[browser](http://genome.ucsc.edu/cgi-bin/hgTracks?position=chr7:68797185-68797410&db=hg19&ss=../trash/hgSs/hgSs_genome_7981_ca98f0.pslx+../trash/hgSs/hgSs_genome_7981_ca98f0.fa&hgsid=258719997) [details](http://genome.ucsc.edu/cgi-bin/hgc?o=68797184&g=htcUserAli&i=../trash/hgSs/hgSs_genome_7981_ca98f0.pslx+..%2Ftrash%2FhgSs%2FhgSs_genome_7981_ca98f0.fa+YourSeq&c=chr7&l=68797184&r=68797410&db=hg19&hgsid=258719997) YourSeq 136 1 226 232 80.1% 7 - 68797185 68797410 226

[browser](http://genome.ucsc.edu/cgi-bin/hgTracks?position=chr9:5097657-5097838&db=hg19&ss=../trash/hgSs/hgSs_genome_7981_ca98f0.pslx+../trash/hgSs/hgSs_genome_7981_ca98f0.fa&hgsid=258719997) [details](http://genome.ucsc.edu/cgi-bin/hgc?o=5097656&g=htcUserAli&i=../trash/hgSs/hgSs_genome_7981_ca98f0.pslx+..%2Ftrash%2FhgSs%2FhgSs_genome_7981_ca98f0.fa+YourSeq&c=chr9&l=5097656&r=5097838&db=hg19&hgsid=258719997) YourSeq 124 51 232 232 84.1% 9 + 5097657 5097838 182

[browser](http://genome.ucsc.edu/cgi-bin/hgTracks?position=chr1:107347475-107347637&db=hg19&ss=../trash/hgSs/hgSs_genome_7981_ca98f0.pslx+../trash/hgSs/hgSs_genome_7981_ca98f0.fa&hgsid=258719997) [details](http://genome.ucsc.edu/cgi-bin/hgc?o=107347474&g=htcUserAli&i=../trash/hgSs/hgSs_genome_7981_ca98f0.pslx+..%2Ftrash%2FhgSs%2FhgSs_genome_7981_ca98f0.fa+YourSeq&c=chr1&l=107347474&r=107347637&db=hg19&hgsid=258719997) YourSeq 109 70 232 232 83.5% 1 - 107347475 107347637 163

[browser](http://genome.ucsc.edu/cgi-bin/hgTracks?position=chr4:156381215-156381377&db=hg19&ss=../trash/hgSs/hgSs_genome_7981_ca98f0.pslx+../trash/hgSs/hgSs_genome_7981_ca98f0.fa&hgsid=258719997) [details](http://genome.ucsc.edu/cgi-bin/hgc?o=156381214&g=htcUserAli&i=../trash/hgSs/hgSs_genome_7981_ca98f0.pslx+..%2Ftrash%2FhgSs%2FhgSs_genome_7981_ca98f0.fa+YourSeq&c=chr4&l=156381214&r=156381377&db=hg19&hgsid=258719997) YourSeq 104 70 232 232 82.1% 4 - 156381215 156381377 163

[browser](http://genome.ucsc.edu/cgi-bin/hgTracks?position=chr11:103275405-103275580&db=hg19&ss=../trash/hgSs/hgSs_genome_7981_ca98f0.pslx+../trash/hgSs/hgSs_genome_7981_ca98f0.fa&hgsid=258719997) [details](http://genome.ucsc.edu/cgi-bin/hgc?o=103275404&g=htcUserAli&i=../trash/hgSs/hgSs_genome_7981_ca98f0.pslx+..%2Ftrash%2FhgSs%2FhgSs_genome_7981_ca98f0.fa+YourSeq&c=chr11&l=103275404&r=103275580&db=hg19&hgsid=258719997) YourSeq 93 51 226 232 84.4% 11 - 103275405 103275580 176

[browser](http://genome.ucsc.edu/cgi-bin/hgTracks?position=chr8:111945587-111945708&db=hg19&ss=../trash/hgSs/hgSs_genome_7981_ca98f0.pslx+../trash/hgSs/hgSs_genome_7981_ca98f0.fa&hgsid=258719997) [details](http://genome.ucsc.edu/cgi-bin/hgc?o=111945586&g=htcUserAli&i=../trash/hgSs/hgSs_genome_7981_ca98f0.pslx+..%2Ftrash%2FhgSs%2FhgSs_genome_7981_ca98f0.fa+YourSeq&c=chr8&l=111945586&r=111945708&db=hg19&hgsid=258719997) YourSeq 80 111 232 232 82.8% 8 - 111945587 111945708 122

[browser](http://genome.ucsc.edu/cgi-bin/hgTracks?position=chr2:203478973-203479070&db=hg19&ss=../trash/hgSs/hgSs_genome_7981_ca98f0.pslx+../trash/hgSs/hgSs_genome_7981_ca98f0.fa&hgsid=258719997) [details](http://genome.ucsc.edu/cgi-bin/hgc?o=203478972&g=htcUserAli&i=../trash/hgSs/hgSs_genome_7981_ca98f0.pslx+..%2Ftrash%2FhgSs%2FhgSs_genome_7981_ca98f0.fa+YourSeq&c=chr2&l=203478972&r=203479070&db=hg19&hgsid=258719997) YourSeq 76 135 232 232 88.8% 2 + 203478973 203479070 98

[browser](http://genome.ucsc.edu/cgi-bin/hgTracks?position=chr8:121236576-121236661&db=hg19&ss=../trash/hgSs/hgSs_genome_7981_ca98f0.pslx+../trash/hgSs/hgSs_genome_7981_ca98f0.fa&hgsid=258719997) [details](http://genome.ucsc.edu/cgi-bin/hgc?o=121236575&g=htcUserAli&i=../trash/hgSs/hgSs_genome_7981_ca98f0.pslx+..%2Ftrash%2FhgSs%2FhgSs_genome_7981_ca98f0.fa+YourSeq&c=chr8&l=121236575&r=121236661&db=hg19&hgsid=258719997) YourSeq 63 51 136 232 87.1% 8 - 121236576 121236661 86

[browser](http://genome.ucsc.edu/cgi-bin/hgTracks?position=chr5:5395721-5395797&db=hg19&ss=../trash/hgSs/hgSs_genome_7981_ca98f0.pslx+../trash/hgSs/hgSs_genome_7981_ca98f0.fa&hgsid=258719997) [details](http://genome.ucsc.edu/cgi-bin/hgc?o=5395720&g=htcUserAli&i=../trash/hgSs/hgSs_genome_7981_ca98f0.pslx+..%2Ftrash%2FhgSs%2FhgSs_genome_7981_ca98f0.fa+YourSeq&c=chr5&l=5395720&r=5395797&db=hg19&hgsid=258719997) YourSeq 51 1 77 232 83.2% 5 - 5395721 5395797 77

[browser](http://genome.ucsc.edu/cgi-bin/hgTracks?position=chr16:3418079-3418134&db=hg19&ss=../trash/hgSs/hgSs_genome_7981_ca98f0.pslx+../trash/hgSs/hgSs_genome_7981_ca98f0.fa&hgsid=258719997) [details](http://genome.ucsc.edu/cgi-bin/hgc?o=3418078&g=htcUserAli&i=../trash/hgSs/hgSs_genome_7981_ca98f0.pslx+..%2Ftrash%2FhgSs%2FhgSs_genome_7981_ca98f0.fa+YourSeq&c=chr16&l=3418078&r=3418134&db=hg19&hgsid=258719997) YourSeq 48 177 232 232 92.9% 16 - 3418079 3418134 56

[browser](http://genome.ucsc.edu/cgi-bin/hgTracks?position=chr10:71351521-71351582&db=hg19&ss=../trash/hgSs/hgSs_genome_7981_ca98f0.pslx+../trash/hgSs/hgSs_genome_7981_ca98f0.fa&hgsid=258719997) [details](http://genome.ucsc.edu/cgi-bin/hgc?o=71351520&g=htcUserAli&i=../trash/hgSs/hgSs_genome_7981_ca98f0.pslx+..%2Ftrash%2FhgSs%2FhgSs_genome_7981_ca98f0.fa+YourSeq&c=chr10&l=71351520&r=71351582&db=hg19&hgsid=258719997) YourSeq 46 171 232 232 87.1% 10 - 71351521 71351582 62

[browser](http://genome.ucsc.edu/cgi-bin/hgTracks?position=chr5:5396969-5397015&db=hg19&ss=../trash/hgSs/hgSs_genome_7981_ca98f0.pslx+../trash/hgSs/hgSs_genome_7981_ca98f0.fa&hgsid=258719997) [details](http://genome.ucsc.edu/cgi-bin/hgc?o=5396968&g=htcUserAli&i=../trash/hgSs/hgSs_genome_7981_ca98f0.pslx+..%2Ftrash%2FhgSs%2FhgSs_genome_7981_ca98f0.fa+YourSeq&c=chr5&l=5396968&r=5397015&db=hg19&hgsid=258719997) YourSeq 39 171 217 232 91.5% 5 - 5396969 5397015 47

[browser](http://genome.ucsc.edu/cgi-bin/hgTracks?position=chr17:78591382-78591422&db=hg19&ss=../trash/hgSs/hgSs_genome_7981_ca98f0.pslx+../trash/hgSs/hgSs_genome_7981_ca98f0.fa&hgsid=258719997) [details](http://genome.ucsc.edu/cgi-bin/hgc?o=78591381&g=htcUserAli&i=../trash/hgSs/hgSs_genome_7981_ca98f0.pslx+..%2Ftrash%2FhgSs%2FhgSs_genome_7981_ca98f0.fa+YourSeq&c=chr17&l=78591381&r=78591422&db=hg19&hgsid=258719997) YourSeq 39 54 94 232 97.6% 17 - 78591382 78591422 41

[browser](http://genome.ucsc.edu/cgi-bin/hgTracks?position=chr3:171252389-171252437&db=hg19&ss=../trash/hgSs/hgSs_genome_7981_ca98f0.pslx+../trash/hgSs/hgSs_genome_7981_ca98f0.fa&hgsid=258719997) [details](http://genome.ucsc.edu/cgi-bin/hgc?o=171252388&g=htcUserAli&i=../trash/hgSs/hgSs_genome_7981_ca98f0.pslx+..%2Ftrash%2FhgSs%2FhgSs_genome_7981_ca98f0.fa+YourSeq&c=chr3&l=171252388&r=171252437&db=hg19&hgsid=258719997) YourSeq 38 33 82 232 90.5% 3 + 171252389 171252437 49

[browser](http://genome.ucsc.edu/cgi-bin/hgTracks?position=chr9:83178495-83178537&db=hg19&ss=../trash/hgSs/hgSs_genome_7981_ca98f0.pslx+../trash/hgSs/hgSs_genome_7981_ca98f0.fa&hgsid=258719997) [details](http://genome.ucsc.edu/cgi-bin/hgc?o=83178494&g=htcUserAli&i=../trash/hgSs/hgSs_genome_7981_ca98f0.pslx+..%2Ftrash%2FhgSs%2FhgSs_genome_7981_ca98f0.fa+YourSeq&c=chr9&l=83178494&r=83178537&db=hg19&hgsid=258719997) YourSeq 37 70 232 232 59.6% 9 - 83178495 83178537 43

[browser](http://genome.ucsc.edu/cgi-bin/hgTracks?position=chr2:85295952-85295995&db=hg19&ss=../trash/hgSs/hgSs_genome_7981_ca98f0.pslx+../trash/hgSs/hgSs_genome_7981_ca98f0.fa&hgsid=258719997) [details](http://genome.ucsc.edu/cgi-bin/hgc?o=85295951&g=htcUserAli&i=../trash/hgSs/hgSs_genome_7981_ca98f0.pslx+..%2Ftrash%2FhgSs%2FhgSs_genome_7981_ca98f0.fa+YourSeq&c=chr2&l=85295951&r=85295995&db=hg19&hgsid=258719997) YourSeq 36 165 208 232 91.0% 2 - 85295952 85295995 44

[browser](http://genome.ucsc.edu/cgi-bin/hgTracks?position=chr2:212644070-212644107&db=hg19&ss=../trash/hgSs/hgSs_genome_7981_ca98f0.pslx+../trash/hgSs/hgSs_genome_7981_ca98f0.fa&hgsid=258719997) [details](http://genome.ucsc.edu/cgi-bin/hgc?o=212644069&g=htcUserAli&i=../trash/hgSs/hgSs_genome_7981_ca98f0.pslx+..%2Ftrash%2FhgSs%2FhgSs_genome_7981_ca98f0.fa+YourSeq&c=chr2&l=212644069&r=212644107&db=hg19&hgsid=258719997) YourSeq 36 171 208 232 97.4% 2 + 212644070 212644107 38

[browser](http://genome.ucsc.edu/cgi-bin/hgTracks?position=chr11:39787886-39787932&db=hg19&ss=../trash/hgSs/hgSs_genome_7981_ca98f0.pslx+../trash/hgSs/hgSs_genome_7981_ca98f0.fa&hgsid=258719997) [details](http://genome.ucsc.edu/cgi-bin/hgc?o=39787885&g=htcUserAli&i=../trash/hgSs/hgSs_genome_7981_ca98f0.pslx+..%2Ftrash%2FhgSs%2FhgSs_genome_7981_ca98f0.fa+YourSeq&c=chr11&l=39787885&r=39787932&db=hg19&hgsid=258719997) YourSeq 34 51 98 232 80.9% 11 + 39787886 39787932 47

[browser](http://genome.ucsc.edu/cgi-bin/hgTracks?position=chr18:50410178-50410197&db=hg19&ss=../trash/hgSs/hgSs_genome_7981_ca98f0.pslx+../trash/hgSs/hgSs_genome_7981_ca98f0.fa&hgsid=258719997) [details](http://genome.ucsc.edu/cgi-bin/hgc?o=50410177&g=htcUserAli&i=../trash/hgSs/hgSs_genome_7981_ca98f0.pslx+..%2Ftrash%2FhgSs%2FhgSs_genome_7981_ca98f0.fa+YourSeq&c=chr18&l=50410177&r=50410197&db=hg19&hgsid=258719997) YourSeq 20 41 60 232 100.0% 18 - 50410178 50410197 20

**Reads BLAT results**

7: chrM:6910-7010 (-)

GCTGCAGTGCTCTGAGCCCTAGGATTCATCTTTCTTTTCACCGTAGGTGGCCTGACTGGCATTGTATTAGCAAACTCATCACTAGACAT**A**GTACTACACGA

## chr1:567460-567560

GCTGCAGTGCTCTGAGCCCTAGGATTtATtTTTCTTTTCACCGTAGGTGGCCTGACTGGCATTGTATTAGCAAACTCATCACTAGACATcGTACTACACGA

## chr17:51183185-51183285

GCTGCgGTGCTCTGAGCCCTgGGATTCATtTTTCTcTTCACtGTAGGcGGCCTaACTGGCATTGTATTAGCAAACTCATCACTAGACATcGTACTACACGA

## chr2:49456770-49456870

GCTGCAGTGCTCTGAGCCCTgGGATTCATtTTTCTcTTCACtGTgGGTGGCCTaACcGGCATTGTATTAGCAAACTCATCACTAGACATcGTACTACACtA

8:chrM:6917-7017(-)

TGCTCTGAGCCCTAGGATTCATCTTTCTTTTCACCGTAGGTGGCCTGACTGGCATTGTATTAGCAAACTCATCACTAGACAT**A**GTACTACACGACACGTAC

## chr1:567467-567567

TGCTCTGAGCCCTAGGATTtATtTTTCTTTTCACCGTAGGTGGCCTGACTGGCATTGTATTAGCAAACTCATCACTAGACATcGTACTACACGACACGTAC

## chr17:51183192-51183292

TGCTCTGAGCCCTgGGATTCATtTTTCTcTTCACtGTAGGcGGCCTaACTGGCATTGTATTAGCAAACTCATCACTAGACATcGTACTACACGACACaTAC

**Primers BLAST results**

>[NT_004350.19](http://www.ncbi.nlm.nih.gov/entrez/viewer.fcgi?db=nucleotide&id=224514624) Homo sapiens chromosome 1 genomic contig, GRCh37.p5 Primary Assembly

product length = 232

Forward primer 1 CCGGCGTCAAAGTATTTAGC 20

Template 46033 .................... 46052

Reverse primer 1 AAGCCTCCTATGATGGCAAA 20

Template 46264 .................... 46245

>[NT_010783.15](http://www.ncbi.nlm.nih.gov/entrez/viewer.fcgi?db=nucleotide&id=224514953) Homo sapiens chromosome 17 genomic contig, GRCh37.p5 Primary Assembly

product length = 232

Forward primer 1 CCGGCGTCAAAGTATTTAGC 20

Template 16457278 .................... 16457297

Reverse primer 1 AAGCCTCCTATGATGGCAAA 20

Template 16457509 .....C...........G.. 16457490

>[NT_011786.16](http://www.ncbi.nlm.nih.gov/entrez/viewer.fcgi?db=nucleotide&id=224514722) Homo sapiens chromosome X genomic contig, GRCh37.p5 Primary Assembly

product length = 232

Forward primer 1 CCGGCGTCAAAGTATTTAGC 20

Template 9873694 ..A................. 9873713

Reverse primer 1 AAGCCTCCTATGATGGCAAA 20

Template 9873925 ..A......G.......G.. 9873906

>[NT_034772.6](http://www.ncbi.nlm.nih.gov/entrez/viewer.fcgi?db=nucleotide&id=224514970) Homo sapiens chromosome 5 genomic contig, GRCh37.p5 Primary Assembly

product length = 232

Forward primer 1 CCGGCGTCAAAGTATTTAGC 20

Template 7703851 .T.................T 7703832

Reverse primer 1 AAGCCTCCTATGATGGCAAA 20

Template 7703620 ..A..C...........G.. 7703639

>[NT_030059.13](http://www.ncbi.nlm.nih.gov/entrez/viewer.fcgi?db=nucleotide&id=224514917) Homo sapiens chromosome 10 genomic contig, GRCh37.p5 Primary Assembly

product length = 230

Forward primer 1 CCGGCGTCAAAGTATTTAGC 20

Template 22156214 .T..T........C...... 22156195

Reverse primer 1 AAGCCTCCTATGATGGCAAA 20

Template 22155985 ...........A.....G.. 22156004

>[NT_007933.15](http://www.ncbi.nlm.nih.gov/entrez/viewer.fcgi?db=nucleotide&id=224514692) Homo sapiens chromosome 7 genomic contig, GRCh37.p5 Primary Assembly

product length = 232

Forward primer 1 CCGGCGTCAAAGTATTTAGC 20

Template 6830253 .T.....T............ 6830234

Reverse primer 1 AAGCCTCCTATGATGGCAAA 20

Template 6830022 ....TG.....A........ 6830041

>[NT_022135.16](http://www.ncbi.nlm.nih.gov/entrez/viewer.fcgi?db=nucleotide&id=224514673) Homo sapiens chromosome 2 genomic contig, GRCh37.p5 Primary Assembly

product length = 232

Forward primer 1 CCGGCGTCAAAGTATTTAGC 20

Template 21889469 .T..T........C...... 21889450

Reverse primer 1 AAGCCTCCTATGATGGCAAA 20

Template 21889238 ........C..A........ 21889257

>[NT_008046.16](http://www.ncbi.nlm.nih.gov/entrez/viewer.fcgi?db=nucleotide&id=224514806) Homo sapiens chromosome 8 genomic contig, GRCh37.p5 Primary Assembly

product length = 232

Forward primer 1 CCGGCGTCAAAGTATTTAGC 20

Template 25219367 .T...A.......C.....T 25219348

Reverse primer 1 AAGCCTCCTATGATGGCAAA 20

Template 25219136 ...........A..A..... 25219155

**Numts aligned to the variant on the UCSC genome browser**


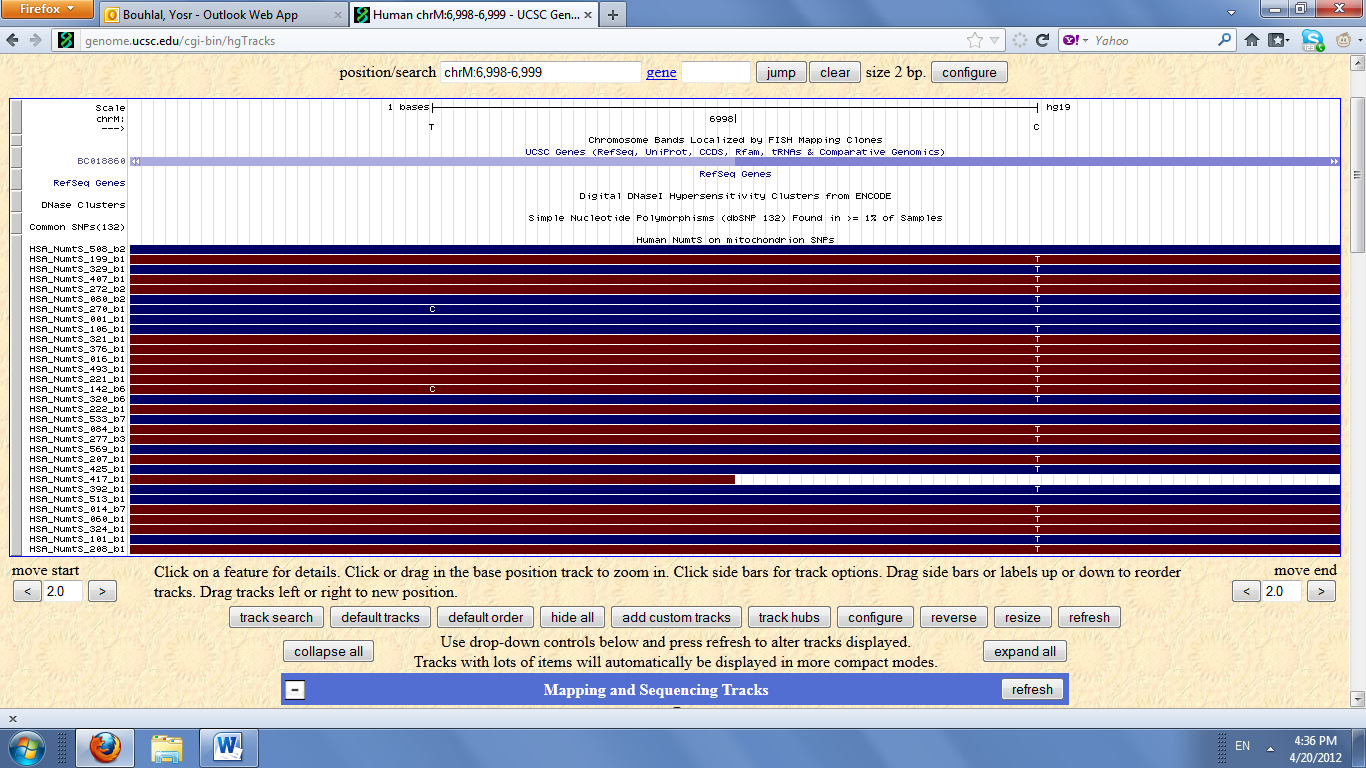


30 Numts: 23T

**Variant8: m.12258C>A**

**Template Fragment**

**AGAGGCTTACGACCCCTTA**TTTACCGAGAAAGCTCACAAGAACTGCTAACTCATGCCCCCATGTCTAACAACATGG**C**TTTCTCAACTTTTAAAGGATAACAGCTATCCATTGGTCTTAGGCCCCAAAAATTT**TGGTGCAACTCCAAATAAAA**

**Template BLAT results**

ACTIONS QUERY SCORE START END QSIZE IDENTITY CHRO STRAND START END SPAN

---------------------------------------------------------------------------------------------------

[browser](http://genome.ucsc.edu/cgi-bin/hgTracks?position=chrM:12183-12334&db=hg19&ss=../trash/hgSs/hgSs_genome_2cc3_da4900.pslx+../trash/hgSs/hgSs_genome_2cc3_da4900.fa&hgsid=259405959) [details](http://genome.ucsc.edu/cgi-bin/hgc?o=12182&g=htcUserAli&i=../trash/hgSs/hgSs_genome_2cc3_da4900.pslx+..%2Ftrash%2FhgSs%2FhgSs_genome_2cc3_da4900.fa+YourSeq&c=chrM&l=12182&r=12334&db=hg19&hgsid=259405959) YourSeq 152 1 152 152 100.0% M + 12183 12334 152

[browser](http://genome.ucsc.edu/cgi-bin/hgTracks?position=chr5:134262153-134262304&db=hg19&ss=../trash/hgSs/hgSs_genome_2cc3_da4900.pslx+../trash/hgSs/hgSs_genome_2cc3_da4900.fa&hgsid=259405959) [details](http://genome.ucsc.edu/cgi-bin/hgc?o=134262152&g=htcUserAli&i=../trash/hgSs/hgSs_genome_2cc3_da4900.pslx+..%2Ftrash%2FhgSs%2FhgSs_genome_2cc3_da4900.fa+YourSeq&c=chr5&l=134262152&r=134262304&db=hg19&hgsid=259405959) YourSeq 138 1 152 152 95.4% 5 - 134262153 134262304 152

[browser](http://genome.ucsc.edu/cgi-bin/hgTracks?position=chr5:99384493-99384644&db=hg19&ss=../trash/hgSs/hgSs_genome_2cc3_da4900.pslx+../trash/hgSs/hgSs_genome_2cc3_da4900.fa&hgsid=259405959) [details](http://genome.ucsc.edu/cgi-bin/hgc?o=99384492&g=htcUserAli&i=../trash/hgSs/hgSs_genome_2cc3_da4900.pslx+..%2Ftrash%2FhgSs%2FhgSs_genome_2cc3_da4900.fa+YourSeq&c=chr5&l=99384492&r=99384644&db=hg19&hgsid=259405959) YourSeq 134 1 152 152 94.1% 5 - 99384493 99384644 152

[browser](http://genome.ucsc.edu/cgi-bin/hgTracks?position=chr10:36723416-36723552&db=hg19&ss=../trash/hgSs/hgSs_genome_2cc3_da4900.pslx+../trash/hgSs/hgSs_genome_2cc3_da4900.fa&hgsid=259405959) [details](http://genome.ucsc.edu/cgi-bin/hgc?o=36723415&g=htcUserAli&i=../trash/hgSs/hgSs_genome_2cc3_da4900.pslx+..%2Ftrash%2FhgSs%2FhgSs_genome_2cc3_da4900.fa+YourSeq&c=chr10&l=36723415&r=36723552&db=hg19&hgsid=259405959) YourSeq 109 16 152 152 89.8% 10 - 36723416 36723552 137

[browser](http://genome.ucsc.edu/cgi-bin/hgTracks?position=chr2:202077814-202077950&db=hg19&ss=../trash/hgSs/hgSs_genome_2cc3_da4900.pslx+../trash/hgSs/hgSs_genome_2cc3_da4900.fa&hgsid=259405959) [details](http://genome.ucsc.edu/cgi-bin/hgc?o=202077813&g=htcUserAli&i=../trash/hgSs/hgSs_genome_2cc3_da4900.pslx+..%2Ftrash%2FhgSs%2FhgSs_genome_2cc3_da4900.fa+YourSeq&c=chr2&l=202077813&r=202077950&db=hg19&hgsid=259405959) YourSeq 107 16 152 152 89.1% 2 - 202077814 202077950 137

[browser](http://genome.ucsc.edu/cgi-bin/hgTracks?position=chr2:132129670-132129806&db=hg19&ss=../trash/hgSs/hgSs_genome_2cc3_da4900.pslx+../trash/hgSs/hgSs_genome_2cc3_da4900.fa&hgsid=259405959) [details](http://genome.ucsc.edu/cgi-bin/hgc?o=132129669&g=htcUserAli&i=../trash/hgSs/hgSs_genome_2cc3_da4900.pslx+..%2Ftrash%2FhgSs%2FhgSs_genome_2cc3_da4900.fa+YourSeq&c=chr2&l=132129669&r=132129806&db=hg19&hgsid=259405959) YourSeq 107 16 152 152 89.1% 2 - 132129670 132129806 137

[browser](http://genome.ucsc.edu/cgi-bin/hgTracks?position=chr7:57261841-57261977&db=hg19&ss=../trash/hgSs/hgSs_genome_2cc3_da4900.pslx+../trash/hgSs/hgSs_genome_2cc3_da4900.fa&hgsid=259405959) [details](http://genome.ucsc.edu/cgi-bin/hgc?o=57261840&g=htcUserAli&i=../trash/hgSs/hgSs_genome_2cc3_da4900.pslx+..%2Ftrash%2FhgSs%2FhgSs_genome_2cc3_da4900.fa+YourSeq&c=chr7&l=57261840&r=57261977&db=hg19&hgsid=259405959) YourSeq 107 16 152 152 89.1% 7 + 57261841 57261977 137

[browser](http://genome.ucsc.edu/cgi-bin/hgTracks?position=chr2:131037728-131037864&db=hg19&ss=../trash/hgSs/hgSs_genome_2cc3_da4900.pslx+../trash/hgSs/hgSs_genome_2cc3_da4900.fa&hgsid=259405959) [details](http://genome.ucsc.edu/cgi-bin/hgc?o=131037727&g=htcUserAli&i=../trash/hgSs/hgSs_genome_2cc3_da4900.pslx+..%2Ftrash%2FhgSs%2FhgSs_genome_2cc3_da4900.fa+YourSeq&c=chr2&l=131037727&r=131037864&db=hg19&hgsid=259405959) YourSeq 105 16 152 152 88.4% 2 + 131037728 131037864 137

[browser](http://genome.ucsc.edu/cgi-bin/hgTracks?position=chr12:130800155-130800291&db=hg19&ss=../trash/hgSs/hgSs_genome_2cc3_da4900.pslx+../trash/hgSs/hgSs_genome_2cc3_da4900.fa&hgsid=259405959) [details](http://genome.ucsc.edu/cgi-bin/hgc?o=130800154&g=htcUserAli&i=../trash/hgSs/hgSs_genome_2cc3_da4900.pslx+..%2Ftrash%2FhgSs%2FhgSs_genome_2cc3_da4900.fa+YourSeq&c=chr12&l=130800154&r=130800291&db=hg19&hgsid=259405959) YourSeq 103 16 152 152 87.6% 12 - 130800155 130800291 137

[browser](http://genome.ucsc.edu/cgi-bin/hgTracks?position=chr8:68497636-68497772&db=hg19&ss=../trash/hgSs/hgSs_genome_2cc3_da4900.pslx+../trash/hgSs/hgSs_genome_2cc3_da4900.fa&hgsid=259405959) [details](http://genome.ucsc.edu/cgi-bin/hgc?o=68497635&g=htcUserAli&i=../trash/hgSs/hgSs_genome_2cc3_da4900.pslx+..%2Ftrash%2FhgSs%2FhgSs_genome_2cc3_da4900.fa+YourSeq&c=chr8&l=68497635&r=68497772&db=hg19&hgsid=259405959) YourSeq 101 16 152 152 86.9% 8 - 68497636 68497772 137

[browser](http://genome.ucsc.edu/cgi-bin/hgTracks?position=chr2:143854131-143854267&db=hg19&ss=../trash/hgSs/hgSs_genome_2cc3_da4900.pslx+../trash/hgSs/hgSs_genome_2cc3_da4900.fa&hgsid=259405959) [details](http://genome.ucsc.edu/cgi-bin/hgc?o=143854130&g=htcUserAli&i=../trash/hgSs/hgSs_genome_2cc3_da4900.pslx+..%2Ftrash%2FhgSs%2FhgSs_genome_2cc3_da4900.fa+YourSeq&c=chr2&l=143854130&r=143854267&db=hg19&hgsid=259405959) YourSeq 99 16 152 152 86.2% 2 - 143854131 143854267 137

[browser](http://genome.ucsc.edu/cgi-bin/hgTracks?position=chr7:57238542-57238678&db=hg19&ss=../trash/hgSs/hgSs_genome_2cc3_da4900.pslx+../trash/hgSs/hgSs_genome_2cc3_da4900.fa&hgsid=259405959) [details](http://genome.ucsc.edu/cgi-bin/hgc?o=57238541&g=htcUserAli&i=../trash/hgSs/hgSs_genome_2cc3_da4900.pslx+..%2Ftrash%2FhgSs%2FhgSs_genome_2cc3_da4900.fa+YourSeq&c=chr7&l=57238541&r=57238678&db=hg19&hgsid=259405959) YourSeq 99 16 152 152 86.2% 7 + 57238542 57238678 137

[browser](http://genome.ucsc.edu/cgi-bin/hgTracks?position=chr1:181391981-181392111&db=hg19&ss=../trash/hgSs/hgSs_genome_2cc3_da4900.pslx+../trash/hgSs/hgSs_genome_2cc3_da4900.fa&hgsid=259405959) [details](http://genome.ucsc.edu/cgi-bin/hgc?o=181391980&g=htcUserAli&i=../trash/hgSs/hgSs_genome_2cc3_da4900.pslx+..%2Ftrash%2FhgSs%2FhgSs_genome_2cc3_da4900.fa+YourSeq&c=chr1&l=181391980&r=181392111&db=hg19&hgsid=259405959) YourSeq 99 22 152 152 87.8% 1 + 181391981 181392111 131

[browser](http://genome.ucsc.edu/cgi-bin/hgTracks?position=chr16:10815355-10815491&db=hg19&ss=../trash/hgSs/hgSs_genome_2cc3_da4900.pslx+../trash/hgSs/hgSs_genome_2cc3_da4900.fa&hgsid=259405959) [details](http://genome.ucsc.edu/cgi-bin/hgc?o=10815354&g=htcUserAli&i=../trash/hgSs/hgSs_genome_2cc3_da4900.pslx+..%2Ftrash%2FhgSs%2FhgSs_genome_2cc3_da4900.fa+YourSeq&c=chr16&l=10815354&r=10815491&db=hg19&hgsid=259405959) YourSeq 97 16 152 152 85.5% 16 - 10815355 10815491 137

[browser](http://genome.ucsc.edu/cgi-bin/hgTracks?position=chr19:57433649-57433785&db=hg19&ss=../trash/hgSs/hgSs_genome_2cc3_da4900.pslx+../trash/hgSs/hgSs_genome_2cc3_da4900.fa&hgsid=259405959) [details](http://genome.ucsc.edu/cgi-bin/hgc?o=57433648&g=htcUserAli&i=../trash/hgSs/hgSs_genome_2cc3_da4900.pslx+..%2Ftrash%2FhgSs%2FhgSs_genome_2cc3_da4900.fa+YourSeq&c=chr19&l=57433648&r=57433785&db=hg19&hgsid=259405959) YourSeq 95 16 152 152 84.7% 19 + 57433649 57433785 137

[browser](http://genome.ucsc.edu/cgi-bin/hgTracks?position=chrX:69347270-69347402&db=hg19&ss=../trash/hgSs/hgSs_genome_2cc3_da4900.pslx+../trash/hgSs/hgSs_genome_2cc3_da4900.fa&hgsid=259405959) [details](http://genome.ucsc.edu/cgi-bin/hgc?o=69347269&g=htcUserAli&i=../trash/hgSs/hgSs_genome_2cc3_da4900.pslx+..%2Ftrash%2FhgSs%2FhgSs_genome_2cc3_da4900.fa+YourSeq&c=chrX&l=69347269&r=69347402&db=hg19&hgsid=259405959) YourSeq 94 16 152 152 82.9% X - 69347270 69347402 133

[browser](http://genome.ucsc.edu/cgi-bin/hgTracks?position=chr4:156375999-156376114&db=hg19&ss=../trash/hgSs/hgSs_genome_2cc3_da4900.pslx+../trash/hgSs/hgSs_genome_2cc3_da4900.fa&hgsid=259405959) [details](http://genome.ucsc.edu/cgi-bin/hgc?o=156375998&g=htcUserAli&i=../trash/hgSs/hgSs_genome_2cc3_da4900.pslx+..%2Ftrash%2FhgSs%2FhgSs_genome_2cc3_da4900.fa+YourSeq&c=chr4&l=156375998&r=156376114&db=hg19&hgsid=259405959) YourSeq 94 37 152 152 90.6% 4 - 156375999 156376114 116

[browser](http://genome.ucsc.edu/cgi-bin/hgTracks?position=chr1:235703146-235703271&db=hg19&ss=../trash/hgSs/hgSs_genome_2cc3_da4900.pslx+../trash/hgSs/hgSs_genome_2cc3_da4900.fa&hgsid=259405959) [details](http://genome.ucsc.edu/cgi-bin/hgc?o=235703145&g=htcUserAli&i=../trash/hgSs/hgSs_genome_2cc3_da4900.pslx+..%2Ftrash%2FhgSs%2FhgSs_genome_2cc3_da4900.fa+YourSeq&c=chr1&l=235703145&r=235703271&db=hg19&hgsid=259405959) YourSeq 94 26 151 152 87.4% 1 - 235703146 235703271 126

[browser](http://genome.ucsc.edu/cgi-bin/hgTracks?position=chr11:81265030-81265145&db=hg19&ss=../trash/hgSs/hgSs_genome_2cc3_da4900.pslx+../trash/hgSs/hgSs_genome_2cc3_da4900.fa&hgsid=259405959) [details](http://genome.ucsc.edu/cgi-bin/hgc?o=81265029&g=htcUserAli&i=../trash/hgSs/hgSs_genome_2cc3_da4900.pslx+..%2Ftrash%2FhgSs%2FhgSs_genome_2cc3_da4900.fa+YourSeq&c=chr11&l=81265029&r=81265145&db=hg19&hgsid=259405959) YourSeq 92 37 152 152 89.7% 11 + 81265030 81265145 116

[browser](http://genome.ucsc.edu/cgi-bin/hgTracks?position=chr2:83042717-83042847&db=hg19&ss=../trash/hgSs/hgSs_genome_2cc3_da4900.pslx+../trash/hgSs/hgSs_genome_2cc3_da4900.fa&hgsid=259405959) [details](http://genome.ucsc.edu/cgi-bin/hgc?o=83042716&g=htcUserAli&i=../trash/hgSs/hgSs_genome_2cc3_da4900.pslx+..%2Ftrash%2FhgSs%2FhgSs_genome_2cc3_da4900.fa+YourSeq&c=chr2&l=83042716&r=83042847&db=hg19&hgsid=259405959) YourSeq 91 26 152 152 88.3% 2 + 83042717 83042847 131

[browser](http://genome.ucsc.edu/cgi-bin/hgTracks?position=chr14:84640033-84640144&db=hg19&ss=../trash/hgSs/hgSs_genome_2cc3_da4900.pslx+../trash/hgSs/hgSs_genome_2cc3_da4900.fa&hgsid=259405959) [details](http://genome.ucsc.edu/cgi-bin/hgc?o=84640032&g=htcUserAli&i=../trash/hgSs/hgSs_genome_2cc3_da4900.pslx+..%2Ftrash%2FhgSs%2FhgSs_genome_2cc3_da4900.fa+YourSeq&c=chr14&l=84640032&r=84640144&db=hg19&hgsid=259405959) YourSeq 91 38 152 152 86.7% 14 + 84640033 84640144 112

[browser](http://genome.ucsc.edu/cgi-bin/hgTracks?position=chr15:40426766-40426880&db=hg19&ss=../trash/hgSs/hgSs_genome_2cc3_da4900.pslx+../trash/hgSs/hgSs_genome_2cc3_da4900.fa&hgsid=259405959) [details](http://genome.ucsc.edu/cgi-bin/hgc?o=40426765&g=htcUserAli&i=../trash/hgSs/hgSs_genome_2cc3_da4900.pslx+..%2Ftrash%2FhgSs%2FhgSs_genome_2cc3_da4900.fa+YourSeq&c=chr15&l=40426765&r=40426880&db=hg19&hgsid=259405959) YourSeq 90 38 152 152 89.5% 15 - 40426766 40426880 115

[browser](http://genome.ucsc.edu/cgi-bin/hgTracks?position=chr9:5109374-5109601&db=hg19&ss=../trash/hgSs/hgSs_genome_2cc3_da4900.pslx+../trash/hgSs/hgSs_genome_2cc3_da4900.fa&hgsid=259405959) [details](http://genome.ucsc.edu/cgi-bin/hgc?o=5109373&g=htcUserAli&i=../trash/hgSs/hgSs_genome_2cc3_da4900.pslx+..%2Ftrash%2FhgSs%2FhgSs_genome_2cc3_da4900.fa+YourSeq&c=chr9&l=5109373&r=5109601&db=hg19&hgsid=259405959) YourSeq 89 37 152 152 88.8% 9 + 5109374 5109601 228

[browser](http://genome.ucsc.edu/cgi-bin/hgTracks?position=chr16:20733610-20733723&db=hg19&ss=../trash/hgSs/hgSs_genome_2cc3_da4900.pslx+../trash/hgSs/hgSs_genome_2cc3_da4900.fa&hgsid=259405959) [details](http://genome.ucsc.edu/cgi-bin/hgc?o=20733609&g=htcUserAli&i=../trash/hgSs/hgSs_genome_2cc3_da4900.pslx+..%2Ftrash%2FhgSs%2FhgSs_genome_2cc3_da4900.fa+YourSeq&c=chr16&l=20733609&r=20733723&db=hg19&hgsid=259405959) YourSeq 88 39 152 152 88.6% 16 - 20733610 20733723 114

[browser](http://genome.ucsc.edu/cgi-bin/hgTracks?position=chr16:69392598-69392709&db=hg19&ss=../trash/hgSs/hgSs_genome_2cc3_da4900.pslx+../trash/hgSs/hgSs_genome_2cc3_da4900.fa&hgsid=259405959) [details](http://genome.ucsc.edu/cgi-bin/hgc?o=69392597&g=htcUserAli&i=../trash/hgSs/hgSs_genome_2cc3_da4900.pslx+..%2Ftrash%2FhgSs%2FhgSs_genome_2cc3_da4900.fa+YourSeq&c=chr16&l=69392597&r=69392709&db=hg19&hgsid=259405959) YourSeq 88 41 152 152 89.3% 16 + 69392598 69392709 112

[browser](http://genome.ucsc.edu/cgi-bin/hgTracks?position=chr2:238432026-238432151&db=hg19&ss=../trash/hgSs/hgSs_genome_2cc3_da4900.pslx+../trash/hgSs/hgSs_genome_2cc3_da4900.fa&hgsid=259405959) [details](http://genome.ucsc.edu/cgi-bin/hgc?o=238432025&g=htcUserAli&i=../trash/hgSs/hgSs_genome_2cc3_da4900.pslx+..%2Ftrash%2FhgSs%2FhgSs_genome_2cc3_da4900.fa+YourSeq&c=chr2&l=238432025&r=238432151&db=hg19&hgsid=259405959) YourSeq 85 26 152 152 85.3% 2 - 238432026 238432151 126

[browser](http://genome.ucsc.edu/cgi-bin/hgTracks?position=chr1:238114846-238114959&db=hg19&ss=../trash/hgSs/hgSs_genome_2cc3_da4900.pslx+../trash/hgSs/hgSs_genome_2cc3_da4900.fa&hgsid=259405959) [details](http://genome.ucsc.edu/cgi-bin/hgc?o=238114845&g=htcUserAli&i=../trash/hgSs/hgSs_genome_2cc3_da4900.pslx+..%2Ftrash%2FhgSs%2FhgSs_genome_2cc3_da4900.fa+YourSeq&c=chr1&l=238114845&r=238114959&db=hg19&hgsid=259405959) YourSeq 83 37 152 152 85.4% 1 - 238114846 238114959 114

[browser](http://genome.ucsc.edu/cgi-bin/hgTracks?position=chr2:120972655-120972762&db=hg19&ss=../trash/hgSs/hgSs_genome_2cc3_da4900.pslx+../trash/hgSs/hgSs_genome_2cc3_da4900.fa&hgsid=259405959) [details](http://genome.ucsc.edu/cgi-bin/hgc?o=120972654&g=htcUserAli&i=../trash/hgSs/hgSs_genome_2cc3_da4900.pslx+..%2Ftrash%2FhgSs%2FhgSs_genome_2cc3_da4900.fa+YourSeq&c=chr2&l=120972654&r=120972762&db=hg19&hgsid=259405959) YourSeq 82 45 152 152 88.0% 2 + 120972655 120972762 108

[browser](http://genome.ucsc.edu/cgi-bin/hgTracks?position=chr4:163342610-163342693&db=hg19&ss=../trash/hgSs/hgSs_genome_2cc3_da4900.pslx+../trash/hgSs/hgSs_genome_2cc3_da4900.fa&hgsid=259405959) [details](http://genome.ucsc.edu/cgi-bin/hgc?o=163342609&g=htcUserAli&i=../trash/hgSs/hgSs_genome_2cc3_da4900.pslx+..%2Ftrash%2FhgSs%2FhgSs_genome_2cc3_da4900.fa+YourSeq&c=chr4&l=163342609&r=163342693&db=hg19&hgsid=259405959) YourSeq 80 69 152 152 97.7% 4 - 163342610 163342693 84

[browser](http://genome.ucsc.edu/cgi-bin/hgTracks?position=chr13:85095771-85095857&db=hg19&ss=../trash/hgSs/hgSs_genome_2cc3_da4900.pslx+../trash/hgSs/hgSs_genome_2cc3_da4900.fa&hgsid=259405959) [details](http://genome.ucsc.edu/cgi-bin/hgc?o=85095770&g=htcUserAli&i=../trash/hgSs/hgSs_genome_2cc3_da4900.pslx+..%2Ftrash%2FhgSs%2FhgSs_genome_2cc3_da4900.fa+YourSeq&c=chr13&l=85095770&r=85095857&db=hg19&hgsid=259405959) YourSeq 62 66 152 152 87.0% 13 + 85095771 85095857 87

[browser](http://genome.ucsc.edu/cgi-bin/hgTracks?position=chr2:125438503-125438579&db=hg19&ss=../trash/hgSs/hgSs_genome_2cc3_da4900.pslx+../trash/hgSs/hgSs_genome_2cc3_da4900.fa&hgsid=259405959) [details](http://genome.ucsc.edu/cgi-bin/hgc?o=125438502&g=htcUserAli&i=../trash/hgSs/hgSs_genome_2cc3_da4900.pslx+..%2Ftrash%2FhgSs%2FhgSs_genome_2cc3_da4900.fa+YourSeq&c=chr2&l=125438502&r=125438579&db=hg19&hgsid=259405959) YourSeq 59 76 152 152 88.4% 2 - 125438503 125438579 77

[browser](http://genome.ucsc.edu/cgi-bin/hgTracks?position=chr8:32663410-32663485&db=hg19&ss=../trash/hgSs/hgSs_genome_2cc3_da4900.pslx+../trash/hgSs/hgSs_genome_2cc3_da4900.fa&hgsid=259405959) [details](http://genome.ucsc.edu/cgi-bin/hgc?o=32663409&g=htcUserAli&i=../trash/hgSs/hgSs_genome_2cc3_da4900.pslx+..%2Ftrash%2FhgSs%2FhgSs_genome_2cc3_da4900.fa+YourSeq&c=chr8&l=32663409&r=32663485&db=hg19&hgsid=259405959) YourSeq 58 76 152 152 87.5% 8 - 32663410 32663485 76

[browser](http://genome.ucsc.edu/cgi-bin/hgTracks?position=chr4:25719536-25719619&db=hg19&ss=../trash/hgSs/hgSs_genome_2cc3_da4900.pslx+../trash/hgSs/hgSs_genome_2cc3_da4900.fa&hgsid=259405959) [details](http://genome.ucsc.edu/cgi-bin/hgc?o=25719535&g=htcUserAli&i=../trash/hgSs/hgSs_genome_2cc3_da4900.pslx+..%2Ftrash%2FhgSs%2FhgSs_genome_2cc3_da4900.fa+YourSeq&c=chr4&l=25719535&r=25719619&db=hg19&hgsid=259405959) YourSeq 58 37 120 152 84.6% 4 - 25719536 25719619 84

[browser](http://genome.ucsc.edu/cgi-bin/hgTracks?position=chr4:66931092-66931167&db=hg19&ss=../trash/hgSs/hgSs_genome_2cc3_da4900.pslx+../trash/hgSs/hgSs_genome_2cc3_da4900.fa&hgsid=259405959) [details](http://genome.ucsc.edu/cgi-bin/hgc?o=66931091&g=htcUserAli&i=../trash/hgSs/hgSs_genome_2cc3_da4900.pslx+..%2Ftrash%2FhgSs%2FhgSs_genome_2cc3_da4900.fa+YourSeq&c=chr4&l=66931091&r=66931167&db=hg19&hgsid=259405959) YourSeq 58 77 152 152 88.2% 4 + 66931092 66931167 76

[browser](http://genome.ucsc.edu/cgi-bin/hgTracks?position=chr3:106613047-106613108&db=hg19&ss=../trash/hgSs/hgSs_genome_2cc3_da4900.pslx+../trash/hgSs/hgSs_genome_2cc3_da4900.fa&hgsid=259405959) [details](http://genome.ucsc.edu/cgi-bin/hgc?o=106613046&g=htcUserAli&i=../trash/hgSs/hgSs_genome_2cc3_da4900.pslx+..%2Ftrash%2FhgSs%2FhgSs_genome_2cc3_da4900.fa+YourSeq&c=chr3&l=106613046&r=106613108&db=hg19&hgsid=259405959) YourSeq 56 37 98 152 95.2% 3 - 106613047 106613108 62

[browser](http://genome.ucsc.edu/cgi-bin/hgTracks?position=chr21:45894984-45895055&db=hg19&ss=../trash/hgSs/hgSs_genome_2cc3_da4900.pslx+../trash/hgSs/hgSs_genome_2cc3_da4900.fa&hgsid=259405959) [details](http://genome.ucsc.edu/cgi-bin/hgc?o=45894983&g=htcUserAli&i=../trash/hgSs/hgSs_genome_2cc3_da4900.pslx+..%2Ftrash%2FhgSs%2FhgSs_genome_2cc3_da4900.fa+YourSeq&c=chr21&l=45894983&r=45895055&db=hg19&hgsid=259405959) YourSeq 54 80 152 152 86.6% 21 - 45894984 45895055 72

[browser](http://genome.ucsc.edu/cgi-bin/hgTracks?position=chrX:102057852-102057909&db=hg19&ss=../trash/hgSs/hgSs_genome_2cc3_da4900.pslx+../trash/hgSs/hgSs_genome_2cc3_da4900.fa&hgsid=259405959) [details](http://genome.ucsc.edu/cgi-bin/hgc?o=102057851&g=htcUserAli&i=../trash/hgSs/hgSs_genome_2cc3_da4900.pslx+..%2Ftrash%2FhgSs%2FhgSs_genome_2cc3_da4900.fa+YourSeq&c=chrX&l=102057851&r=102057909&db=hg19&hgsid=259405959) YourSeq 52 38 95 152 94.9% X + 102057852 102057909 58

[browser](http://genome.ucsc.edu/cgi-bin/hgTracks?position=chr19:38063548-38063606&db=hg19&ss=../trash/hgSs/hgSs_genome_2cc3_da4900.pslx+../trash/hgSs/hgSs_genome_2cc3_da4900.fa&hgsid=259405959) [details](http://genome.ucsc.edu/cgi-bin/hgc?o=38063547&g=htcUserAli&i=../trash/hgSs/hgSs_genome_2cc3_da4900.pslx+..%2Ftrash%2FhgSs%2FhgSs_genome_2cc3_da4900.fa+YourSeq&c=chr19&l=38063547&r=38063606&db=hg19&hgsid=259405959) YourSeq 47 37 95 152 89.9% 19 + 38063548 38063606 59

[browser](http://genome.ucsc.edu/cgi-bin/hgTracks?position=chr2:103122246-103122288&db=hg19&ss=../trash/hgSs/hgSs_genome_2cc3_da4900.pslx+../trash/hgSs/hgSs_genome_2cc3_da4900.fa&hgsid=259405959) [details](http://genome.ucsc.edu/cgi-bin/hgc?o=103122245&g=htcUserAli&i=../trash/hgSs/hgSs_genome_2cc3_da4900.pslx+..%2Ftrash%2FhgSs%2FhgSs_genome_2cc3_da4900.fa+YourSeq&c=chr2&l=103122245&r=103122288&db=hg19&hgsid=259405959) YourSeq 35 110 152 152 90.7% 2 - 103122246 103122288 43

[browser](http://genome.ucsc.edu/cgi-bin/hgTracks?position=chr6:161982333-161982376&db=hg19&ss=../trash/hgSs/hgSs_genome_2cc3_da4900.pslx+../trash/hgSs/hgSs_genome_2cc3_da4900.fa&hgsid=259405959) [details](http://genome.ucsc.edu/cgi-bin/hgc?o=161982332&g=htcUserAli&i=../trash/hgSs/hgSs_genome_2cc3_da4900.pslx+..%2Ftrash%2FhgSs%2FhgSs_genome_2cc3_da4900.fa+YourSeq&c=chr6&l=161982332&r=161982376&db=hg19&hgsid=259405959) YourSeq 34 108 152 152 87.2% 6 - 161982333 161982376 44

[browser](http://genome.ucsc.edu/cgi-bin/hgTracks?position=chr4:41025262-41025285&db=hg19&ss=../trash/hgSs/hgSs_genome_2cc3_da4900.pslx+../trash/hgSs/hgSs_genome_2cc3_da4900.fa&hgsid=259405959) [details](http://genome.ucsc.edu/cgi-bin/hgc?o=41025261&g=htcUserAli&i=../trash/hgSs/hgSs_genome_2cc3_da4900.pslx+..%2Ftrash%2FhgSs%2FhgSs_genome_2cc3_da4900.fa+YourSeq&c=chr4&l=41025261&r=41025285&db=hg19&hgsid=259405959) YourSeq 24 37 60 152 100.0% 4 - 41025262 41025285 24

[browser](http://genome.ucsc.edu/cgi-bin/hgTracks?position=chr15:58444778-58444808&db=hg19&ss=../trash/hgSs/hgSs_genome_2cc3_da4900.pslx+../trash/hgSs/hgSs_genome_2cc3_da4900.fa&hgsid=259405959) [details](http://genome.ucsc.edu/cgi-bin/hgc?o=58444777&g=htcUserAli&i=../trash/hgSs/hgSs_genome_2cc3_da4900.pslx+..%2Ftrash%2FhgSs%2FhgSs_genome_2cc3_da4900.fa+YourSeq&c=chr15&l=58444777&r=58444808&db=hg19&hgsid=259405959) YourSeq 23 26 56 152 87.1% 15 + 58444778 58444808 31

[browser](http://genome.ucsc.edu/cgi-bin/hgTracks?position=chr3:165877695-165877716&db=hg19&ss=../trash/hgSs/hgSs_genome_2cc3_da4900.pslx+../trash/hgSs/hgSs_genome_2cc3_da4900.fa&hgsid=259405959) [details](http://genome.ucsc.edu/cgi-bin/hgc?o=165877694&g=htcUserAli&i=../trash/hgSs/hgSs_genome_2cc3_da4900.pslx+..%2Ftrash%2FhgSs%2FhgSs_genome_2cc3_da4900.fa+YourSeq&c=chr3&l=165877694&r=165877716&db=hg19&hgsid=259405959) YourSeq 22 38 59 152 100.0% 3 - 165877695 165877716 22

[browser](http://genome.ucsc.edu/cgi-bin/hgTracks?position=chr4:162709624-162709643&db=hg19&ss=../trash/hgSs/hgSs_genome_2cc3_da4900.pslx+../trash/hgSs/hgSs_genome_2cc3_da4900.fa&hgsid=259405959) [details](http://genome.ucsc.edu/cgi-bin/hgc?o=162709623&g=htcUserAli&i=../trash/hgSs/hgSs_genome_2cc3_da4900.pslx+..%2Ftrash%2FhgSs%2FhgSs_genome_2cc3_da4900.fa+YourSeq&c=chr4&l=162709623&r=162709643&db=hg19&hgsid=259405959) YourSeq 20 81 100 152 100.0% 4 + 162709624 162709643 20

**Reads BLAT results**

7:chrM:12251-12351(-)

CAACATGG**A**TTTCTCAACTTTTAAAGGATAACAGCTATCCATTGGTCTTAGGCCCCAAAAATTTTGGTGCAACTCCAAATAAAAGTAATAACAATGCACAC

## chr5:134262145-134262236

CAACATGGcTTTCTCAACTTTTAAAGGATAACAGCcATCCgTTGGTCTTAGGCCCCAAAAATTTTGGTGCAACTCCAAATAAAAGTAATAAC

## chr4:163342602-163342693

CAACATGGcTTTCTCAACTTTTAAAGGATAACAGCcATCCgTTGGTCTTAGGCCCCAAAAATTTTGGTGCAACTCCAAATAAAAGTAATAAC

## chr5:99384485-99384576

CAACATGGcTTTCTCgACTTTTAAAGGATAACAGCcATCCgTTGGTCTTAGGCCCCAAAAATTTTGGTGCAACTCCAAATAAAAGTAATAAC

## chr10:36723409-36723499

CAACATGGcTTTCTCAACTTTTAAAGtATAAgAGCgAcCCATTGGTCTTAGGaaCCAAAAATaTTGGTGCAACTCCAAATAAAAGTAATAA

**chr2:202077807-202077897**

CAACATaGtTTTCTCAACTTTTAgAGGATgAgAGtTATCCATTGGTCTTAGGaaCCAAAAATaTTGGTGCAACTCCAAATAAAAGTAATAA

8:chrM:12173-12273(+)

ATCTGACAACAGAGGCTTACGACCCCTTATTTACCGAGAAAGCTCACAAGAACTGCTAACTCATGCCCCCATGTCTAACAACATGG**A**TTTCTCAACTTTTA

## chr5:134262214-134262314

ATCTGACAACAGAGGCTcACGACCCCTTATTTACCGAGAAAGCgCAtAAGAACTGCTAACTCATatCCCCATGTCTAACAACATGGcTTTCTCAACTTTTA

## chr5:99384554-99384654

ATCTGAtAACAGAGGCTcACagCCCCTTATTTACCGAGAAAGCTCAtAAGAACTGCTAACTCATaCtCCCATGTCTAACAACATGGcTTTCTCgACTTTTA

## chr12:130800216-130800291

CTTATcTACCGAGAAAGCatggAAGAACTGCTAACTCATGCCCCCATGcCTAACAACATGGgTTTCTCAACTTTTA

**Primers BLAST results**

>[NT_034772.6](http://www.ncbi.nlm.nih.gov/entrez/viewer.fcgi?db=nucleotide&id=224514970) Homo sapiens chromosome 5 genomic contig, GRCh37.p5 Primary Assembly

product length = 152

Forward primer 1 AGAGGCTTACGACCCCTTAT 20

Template 42576176 .......C............ 42576157

Reverse primer 1 TTTTATTTGGAGTTGCACCA 20

Template 42576025 .................... 42576044

**Numts aligned to the variant on the UCSC genome browser**

39 Numts: 3T, 1G


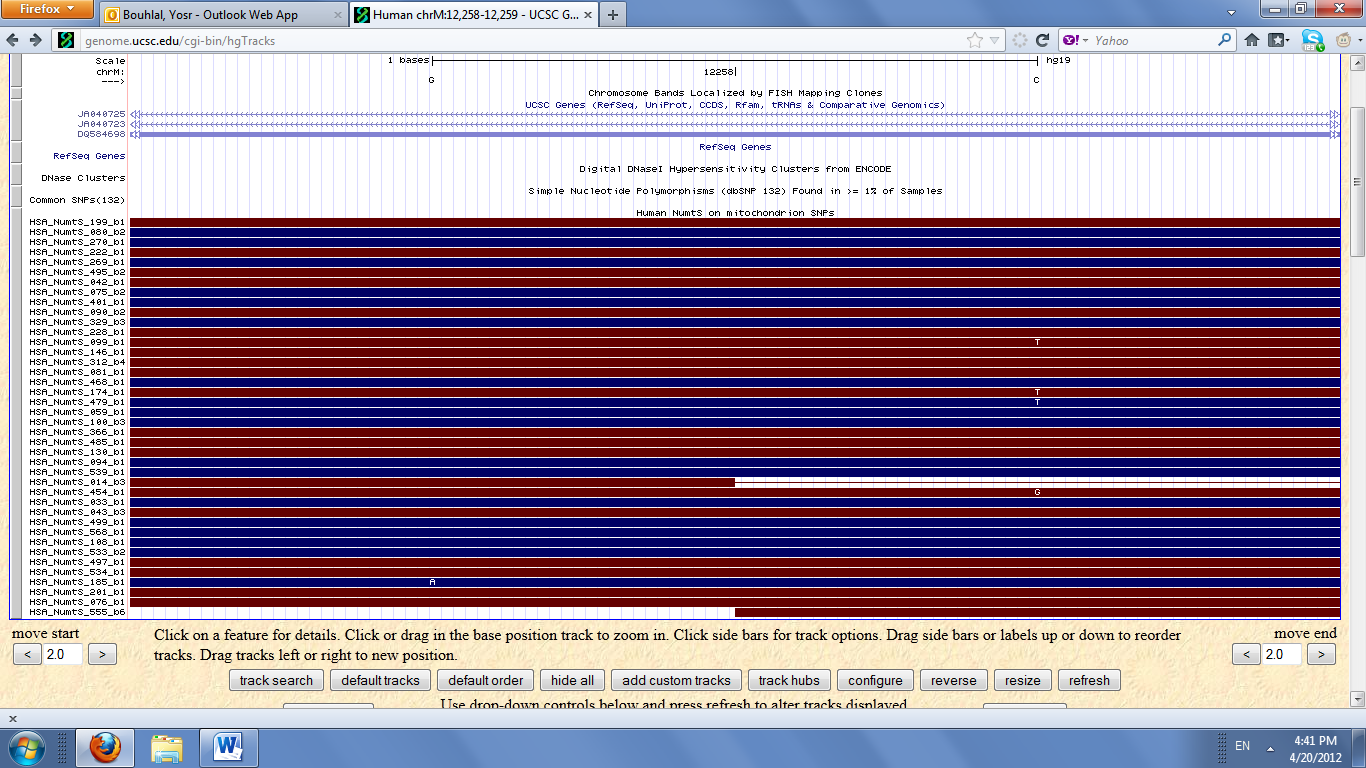

Supplement: Supplementary file 3 [file mgg30001-0174-SD3.docx]
