# Supplementary material for: Twin mitochondrial sequence analysis
Source: Mol Genet Genomic Med. 2013 Jun 26;1(3):174–86. doi: 10.1002/mgg3.20 (PMC3768015; doi:10.1002/mgg3.20)
Supplement: Supplementary file 4 [file mgg30001-0174-SD4.docx]

**Supplementary Table S3:** Sequencing SNP Calling By Phred Quality Score

|  | **Twin A** | **Twin B** |
| --- | --- | --- |
| Total number of reads mapped to the mitochondrial genome | 130026 | 158404 |
| All potential mitochondrial variants | 13374 | 11035 |
| Number of SNPs with Phred Quality Score Q≥10 | 3992 | 4006 |
| Number of SNPs with Phred Quality Score Q≥20 | 1509 | 1523 |
| **Number of SNPs with Phred Quality Score Q**≥**30** | **588** | **967** |
|  |  |  |
